# Limited Gut Microbiome Differences Between Relapsing–Remitting and Secondary Progressive Multiple Sclerosis: A Shotgun Metagenomic Study

**DOI:** 10.3390/ijms27209166

**Published:** 2026-10-16

**Authors:** Cristiana Vacaras, Vitalie Vacaras, Cristina Nistor, Stefan Cristian Vesa, Cristina Jucan, Ioana Monica Furdui, Stefan Strilciuc, Emanuel Stefanescu, Diana Chira, Alexandru Rafila, Dafin F. Muresanu

**Affiliations:** 1 Neurology Department, Cluj Emergency County Hospital, 400012 Cluj-Napoca, Romania; 2 Department of Neurosciences, Faculty of Medicine, Iuliu Hațieganu University of Medicine and Pharmacy, 400012 Cluj-Napoca, Romania; 3 Department of Pharmacology, Toxicology and Clinical Pharmacology, Faculty of Medicine, Iuliu Haţieganu University of Medicine and Pharmacy, 400349 Cluj-Napoca, Romania; 4 Department of Genomics, MEDFUTURE Institute for Biomedical Research, Iuliu Hațieganu University of Medicine and Pharmacy, 400337 Cluj-Napoca, Romania; 5 RoNeuro Institute for Neurological Research and Diagnostic, 400364 Cluj-Napoca, Romania; 6 Department of Microbiology, Carol Davila University of Medicine and Pharmacy, Dionisie Lupu Street, 050474 Bucharest, Romania

**Keywords:** multiple sclerosis, gastrointestinal microbiome, metagenomics, neurological biomarker, relapsing–remitting multiple sclerosis, secondary progressive multiple sclerosis

## Abstract

The gut microbiome may contribute to multiple sclerosis (MS) pathophysiology, but its relationship with disease phenotype and progression remains unclear. To characterize taxonomic and functional gut microbiome differences between relapsing–remitting MS (RRMS) and secondary progressive MS (SPMS) and their associations with neurological disability and circulating biomarkers. This cross-sectional study included 89 participants (30 healthy controls, 29 RRMS, and 30 SPMS). Shotgun metagenomic sequencing was used to assess microbial diversity, taxonomic profiles, and predefined short-chain fatty acid (SCFA)-related pathways, integrated with disability measures and serum GFAP, NfL, and S100B. SPMS participants were substantially older than those with RRMS (median 49.0 vs. 31.0 years), prompting age-adjusted analyses and an age-overlap sensitivity analysis. Alpha diversity and overall microbial community composition were similar across groups. SPMS showed greater interindividual microbial community dispersion than RRMS in the full cohort, with a similar direction and magnitude but attenuated statistical significance in the age-overlap sensitivity analysis. Exploratory taxonomic signals were not confirmed by FDR-controlled analyses. No robust phenotype-specific differences in predicted SCFA-related pathways or associations of taxonomic or functional features with disability or circulating biomarkers were identified. Greater microbial community dispersion in SPMS was observed in the full cohort, but this finding was attenuated in the age-overlap sensitivity analysis and should be considered preliminary. Overall, these findings do not support a distinct SPMS-associated gut microbiome signature, although potential phenotype-related differences cannot be excluded.

## 1. Introduction

Multiple sclerosis (MS) is a chronic demyelinating disease of the central nervous system and a major cause of neurological disability in young adults [1]. Approximately 120,000 new MS diagnoses are estimated to occur annually worldwide, although incidence varies substantially across regions [2]. Clinical manifestations are heterogeneous and commonly include sensory disturbances, motor weakness, visual impairment, gait and balance dysfunction, fatigue, cognitive impairment, and bladder dysfunction, with progressive accumulation of neurological disability occurring in a subset of patients [3].

Relapsing–remitting MS (RRMS) is characterized by clinical relapses and remissions, whereas secondary progressive MS (SPMS) involves progressive disability and increasing neurodegenerative mechanisms. The transition from RRMS to SPMS is gradual and heterogeneous, involving compartmentalized CNS inflammation, chronic glial activation, impaired remyelination, oxidative stress, and neuroaxonal loss [4].

The gut microbiome may contribute to MS pathophysiology through immune, endocrine, and neural pathways of the gut–brain axis [5]. Although microbial alterations have been reported in MS, taxonomic findings remain inconsistent, and most studies have focused on RRMS versus healthy controls (HCs), leaving progressive MS insufficiently characterized [6,7,8].

Previous studies have reported gut microbial alterations in MS, including changes in taxa involved in immune regulation and SCFA metabolism, although the reproducibility of individual taxonomic findings remains limited. Recent evidence further suggests that overall microbial diversity is not consistently associated with MS disease activity, course, or disability progression, whereas specific microbial functions may show more reproducible associations [9]. Similar microbiota–brain interactions have been investigated in other neurological disorders, including Parkinson’s and Alzheimer’s diseases, where microbial metabolites, immune signaling, intestinal barrier dysfunction, and neuroinflammation have been proposed as potential components of the gut–brain axis [10].

Microbial functional potential may provide additional insight into microbiome–host interactions. Short-chain fatty acids (SCFAs), particularly butyrate and propionate, have immunomodulatory effects relevant to MS, and alterations in SCFA-producing taxa have been associated with MS and neurological disability [8,9,11]. Previous studies have reported reduced fecal or circulating SCFAs in MS, including butyrate and propionate, while propionate has been linked to regulatory T-cell responses and other immunomodulatory effects. Moreover, metagenomic and metabolomic analyses across MS phenotypes have identified alterations in butyrate- and propionate-related microbial functions [12]. On this basis, butyrate- and propionate-related microbial functional potential was selected as the principal hypothesis-driven functional domain investigated in the present study.

To complement microbiome profiling with markers of central nervous system injury, we selected three circulating neurological biomarkers reflecting partly distinct biological processes. Neurofilament light chain (NfL) is a marker of neuroaxonal injury and is associated particularly with inflammatory disease activity and tissue damage in MS, whereas glial fibrillary acidic protein (GFAP) reflects astrocytic activation and has been increasingly associated with disability progression and progressive disease biology. S100B, predominantly expressed by astrocytes, has been investigated as a marker of glial injury and blood–brain barrier dysfunction [13,14]. Together, these biomarkers were selected to provide complementary measures of neuroaxonal and astroglial injury that could be integrated with microbial taxonomic and functional profiles.

Accordingly, the present study included healthy controls (HCs), patients with relapsing–remitting MS (RRMS), and patients with secondary progressive MS (SPMS), allowing microbial features associated with MS to be examined in relation to a healthy reference group while also investigating potential differences between relapsing and progressive disease phenotypes.

We hypothesized that RRMS and SPMS differ in gut microbial community structure, taxonomic composition, and predicted functional potential. Therefore, we aimed to characterize taxonomic and predicted functional gut microbiome profiles across HCs, RRMS, and SPMS, with a particular focus on RRMS–SPMS differences and their associations with neurological disability and markers of neuroaxonal and glial injury.

## 2. Results

### 2.1. Clinical Characteristics and Serum Neurological Biomarkers

The study included 89 participants: 30 HCs, 29 patients with RRMS, and 30 with SPMS. Participants with SPMS were older than those in the other groups (median age 49.0 vs. 31.0 years in RRMS and 29.0 years in HCs; *p* < 0.001), while sex distribution was similar (*p* = 0.963). Smoking, alcohol consumption, dietary restrictions, and REAP total scores did not differ significantly between groups (Supplementary Table S1). Among patients with SPMS, 15 had received HE treatment, 13 ME treatment, and two had no history of MS-specific treatment.

Neurological disability and functional performance differed significantly across groups (Table 1). SPMS patients had higher EDSS scores and greater impairment in T25FW, 9HPT, and SDMT performance than both RRMS patients and HCs (all *p* < 0.001). RRMS patients also had longer 9HPT times than HCs for both hands, whereas T25FW and SDMT did not differ significantly between RRMS and HCs.

Disease duration, defined as time since disease onset, was substantially longer in SPMS than in RRMS (204 (147–284) vs. 17 (6–57) months, *p* < 0.001, Wilcoxon/Mann–Whitney test). Annualized relapse rate was higher in RRMS than in SPMS (median [IQR], 1.00 [0.40–1.00] vs. 0.30 [0.19–0.49], respectively; *p* < 0.001, Mann–Whitney test).

Circulating GFAP, NfL, and S100B did not differ significantly among groups. Measurements below the detection limit were observed for S100B in 11/30 (36.7%) HCs, 16/29 (55.2%) RRMS, and 12/30 (40.0%) SPMS participants. Below-detection measurements were uncommon for GFAP (1/30 [3.3%], 1/29 [3.4%], and 0/30 [0%], respectively) and were not observed for NfL. In adjusted RRMS–SPMS analyses, GFAP was nominally higher in SPMS but did not remain significant after multiple-testing correction; NfL and S100B showed no significant differences (Supplementary Table S2a,b).

### 2.2. Gut Microbial Diversity and Heterogeneity

Sequencing yielded a median of 13.2 million raw read pairs per sample (IQR 10.9–16.6). After host-read removal and quality filtering, a median of 13.2 million read pairs (IQR 10.7–16.5; median retention 99.8%) was retained per sample. Sequencing depth did not differ among HCs, RRMS and SPMS (Kruskal–Wallis *p* = 0.73; Supplementary Table S3a,b).

Species-level alpha diversity did not differ among groups (Shannon *p* = 0.937; Simpson *p* = 0.825), with similarly non-significant RRMS–SPMS comparisons after FDR correction (Supplementary Figure S1).

Bray–Curtis beta diversity showed no significant separation among groups or between RRMS and SPMS, including after adjustment for age and sex (Supplementary Figure S2).

**Figure 1:**
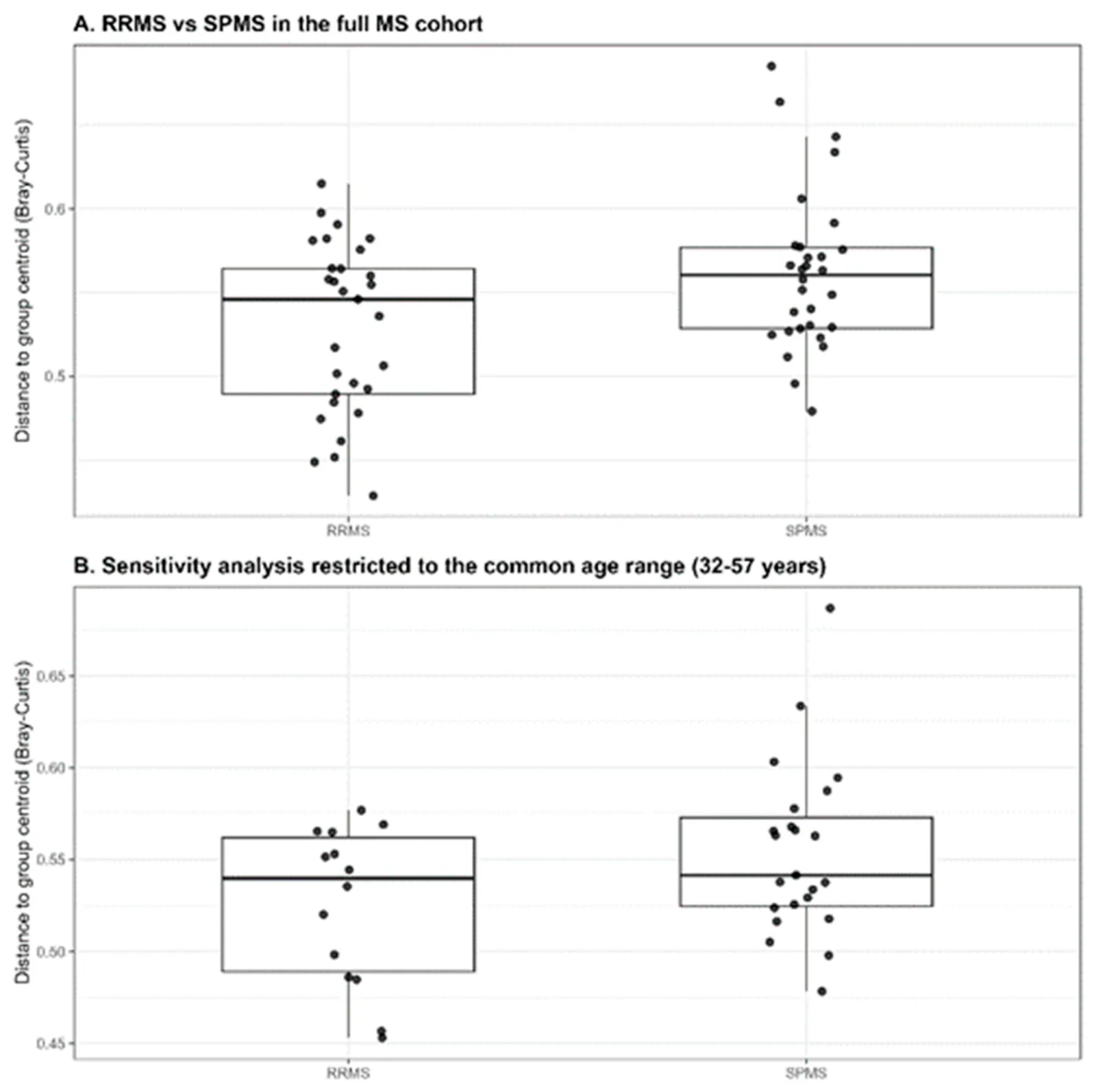
Interindividual gut microbial community dispersion in RRMS and SPMS. (**A**) Bray–Curtis distance to group centroid in the full RRMS–SPMS cohort (RRMS, n = 29; SPMS, n = 30). Dispersion was greater in SPMS than in RRMS (mean distance to centroid 0.562 vs. 0.529; mean difference 0.033, 95% CI 0.007–0.058; PERMDISP *p* = 0.013). (**B**) Sensitivity analysis restricted to participants within the common observed age range of 32–57 years (RRMS, n = 14; SPMS, n = 23). The difference remained similar in direction and magnitude (0.555 vs. 0.526; mean difference 0.029, 95% CI −0.002 to 0.060), but did not reach conventional statistical significance (PERMDISP *p* = 0.069). Individual points represent participants; boxplots show the median and interquartile range.

In the RRMS–SPMS-specific analysis, multivariate dispersion was greater in SPMS than in RRMS (mean distance to centroid 0.562 vs. 0.529; mean difference 0.033, 95% CI 0.007–0.058; PERMDISP *p* = 0.013) (Figure 1A). In the sensitivity analysis restricted to participants within the common observed RRMS–SPMS age range of 32–57 years (RRMS, n = 14; SPMS, n = 23), the direction and magnitude of the difference were largely preserved (0.555 vs. 0.526; mean difference 0.029, 95% CI −0.002 to 0.060), although the estimate was less precise and the difference did not reach conventional statistical significance (PERMDISP *p* = 0.069) (Figure 1B).

Among participants with MS, neither Shannon nor Simpson diversity showed robust associations with neurological disability or circulating neurological biomarkers after adjustment for phenotype, age, sex, and multiple testing. Two nominal associations, between Shannon diversity and non-dominant hand 9HPT performance and between Simpson diversity and EDSS, did not survive FDR correction (both *q* = 0.257; Supplementary Table S4).

### 2.3. Phenotype-Associated Bacterial Taxa

The predefined confirmatory taxonomic analysis included features at the phylum, family, and genus levels. Age- and sex-adjusted pairwise comparisons were additionally performed for RRMS versus HCs and SPMS versus HCs. No predefined taxonomic feature remained significant after FDR correction in either comparison. *Butyricicoccus* detection was nominally lower in RRMS than in HCs (OR 0.27, 95% CI 0.08–0.87; *p* = 0.029, *q* = 0.494), whereas *Faecalibacterium* abundance was nominally lower in SPMS than in HCs (LFC −0.83, 95% CI −1.53 to −0.12; *p* = 0.025, *q* = 0.418); neither association survived multiple-testing correction. No predefined taxon differed significantly among groups or between RRMS and SPMS after FDR correction (Supplementary Table S5).

As a secondary, hypothesis-generating analysis, LEfSe identified several nominal taxonomic signals between each MS phenotype and HCs (Supplementary Figure S3a,b). These exploratory findings were not corrected for multiple testing and were interpreted separately from the predefined FDR-controlled analyses.

**Figure 2:**
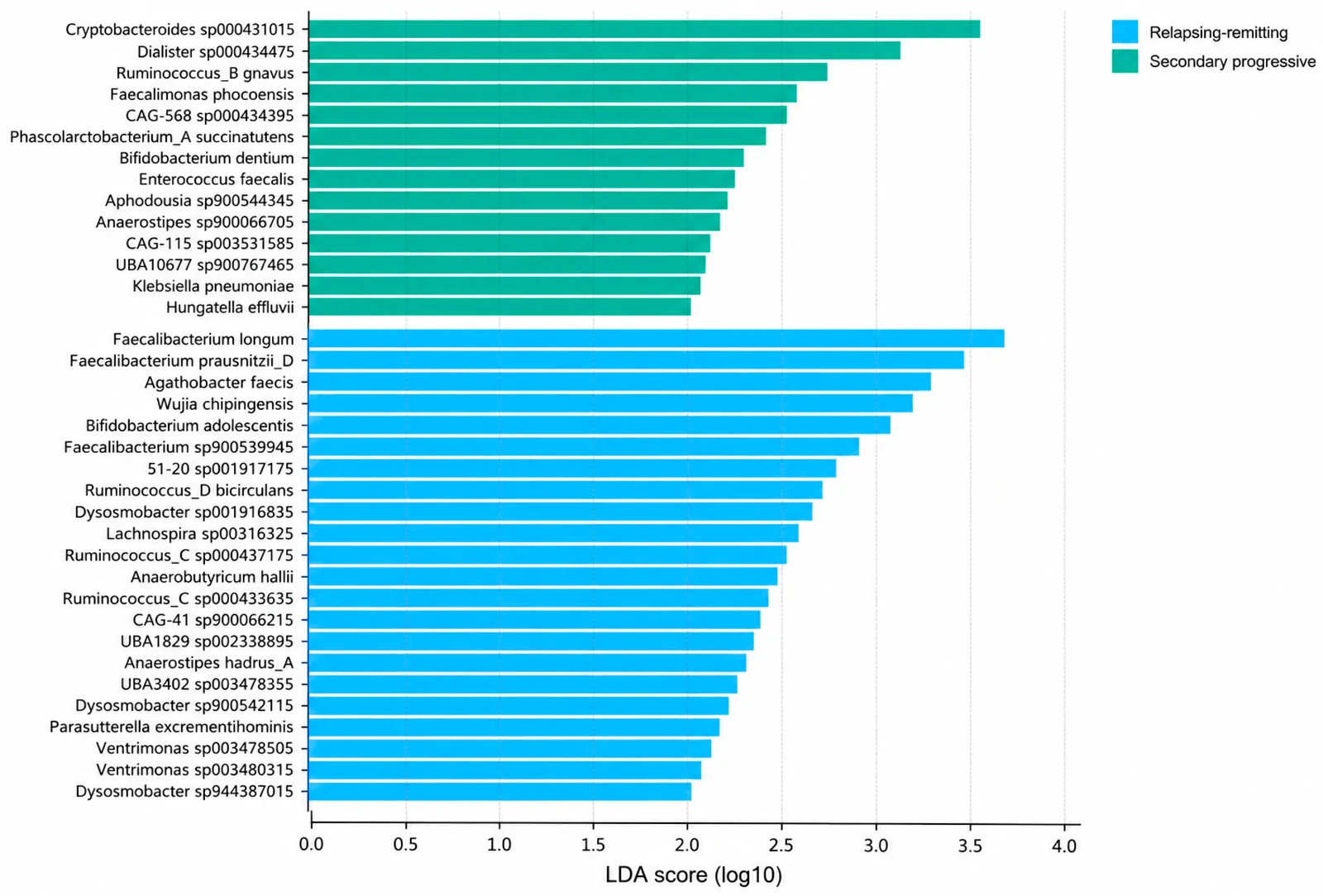
Exploratory species-level LEfSe analysis comparing RRMS and SPMS. Species meeting the prespecified exploratory criteria of nominal *p* < 0.05 and absolute LDA score ≥ 2.0 are shown. Blue bars indicate features enriched in RRMS and green bars features enriched in SPMS. Twenty-two features were enriched in RRMS and 14 in SPMS according to these exploratory criteria. These findings were not corrected for multiple testing, were not corroborated by the predefined FDR-controlled taxonomic analyses, and should therefore be interpreted as hypothesis-generating rather than as evidence of phenotype-specific taxonomic differences.

In the exploratory RRMS–SPMS LEfSe analysis, several taxa met the nominal *p* < 0.05 and absolute LDA score ≥ 2.0 criteria. These included higher relative abundance of *Faecalibacterium longum* (LDA 3.93), *F. prausnitzii_D* (LDA 3.76), *Agathobacter faecis* (LDA 3.71), *Wujia chipingensis* (LDA 3.61), and *Bifidobacterium adolescentis* (LDA 3.42) in RRMS, whereas *Cryptobacteroides* sp000431015 (LDA 3.56), *Dialister sp000434475* (LDA 3.06), and *Ruminococcus_B gnavus* (LDA 2.98) showed higher relative abundance in SPMS (Figure 2). These unadjusted LEfSe findings were considered hypothesis-generating and interpreted separately from the FDR-controlled ANCOM-BC2 analyses.

### 2.4. Predefined Taxonomic Features and Their Associations with Neurological Disability and Biomarkers

The predefined hypothesis-driven taxonomic analysis comprised 17 literature-selected features spanning the phylum, family, and genus levels. No predefined taxon was significantly associated with neurological disability or circulating biomarkers after FDR correction. Nominal associations and two FDR-significant *Akkermansia*-by-phenotype interactions with 9HPT failed pseudo-count sensitivity analyses and were considered non-robust. No robust taxon-by-phenotype interactions were identified (Supplementary Tables S6a,b and S7a,b).

### 2.5. Microbial Functional Profiles Across MS Phenotypes

#### 2.5.1. Prespecified SCFA-Related Functional Analysis

The prespecified functional analysis focused on six SCFA-related GMMs (Gut Metabolic Modules), comprising two butyrate-production pathways, three propionate-production pathways, and succinate conversion to propionate. None of these six GMMs differed significantly among HCs, RRMS, and SPMS after FDR correction. Predicted propionate production III showed a nominal difference in detection across groups (*p* = 0.032), with more frequent detection in SPMS, but this did not survive FDR correction (*q* = 0.188) (Table 2).

#### 2.5.2. Direct RRMS–SPMS Comparison

None of the six prespecified SCFA-related GMMs differed significantly between RRMS and SPMS after FDR correction (Table 3), including in age- and sex-adjusted and age-overlap sensitivity analyses (Supplementary Table S8).

#### 2.5.3. Exploratory Whole-Profile Functional Analysis

To assess whether phenotype-associated functional differences extended beyond the six predefined SCFA-related modules, we performed an exploratory whole-profile analysis of the complete set of available metagenomic functional modules. Of 103 GMMs and 49 GBMs (Gut–Brain Modules), 100 GMMs and 48 GBMs met the predefined prevalence criteria for adjusted analysis. No GMM or GBM differed significantly after FDR correction in RRMS versus HCs, SPMS versus HCs, or SPMS versus RRMS comparisons (Supplementary Table S9a,b).

### 2.6. Prespecified SCFA-Related Microbial Functions and Their Association with Neurological Disability and Biomarkers

No predicted SCFA-related function was significantly associated with neurological disability or circulating biomarkers after FDR correction, and no robust phenotype interactions were identified (Supplementary Tables S10a–c and S11a,b). A nominal phenotype interaction for propionate production I and NfL did not survive FDR correction and was considered exploratory.

### 2.7. Exploratory Nitric Oxide and Redox-Related Microbial Functions

No nitric oxide/redox-related microbial function differed significantly among groups or between RRMS and SPMS after FDR correction, including in adjusted analyses (Supplementary Table S12a–c).

### 2.8. Exploratory Sensitivity Analysis According to Current Treatment Status in SPMS

Among participants with SPMS, 17 were receiving disease-modifying therapy at the time of fecal sampling and 13 were not. Current treatment status was not associated with Shannon (*p* = 0.336) or Simpson (*p* = 0.167) alpha diversity. Overall Bray–Curtis community composition also did not differ according to treatment status (unadjusted PERMANOVA R^2^ = 0.027, *p* = 0.854; age- and sex-adjusted R^2^ = 0.027, *p* = 0.876). Multivariate dispersion was virtually identical between currently treated and untreated participants (mean distance to centroid 0.554 and 0.555, respectively; permutation *p* = 0.978). None of the six predefined SCFA-related functional modules differed according to current treatment status after FDR correction (all *q* ≥ 0.246; Supplementary Table S13).

## 3. Discussion

In this cross-sectional shotgun metagenomic study, we integrated microbiome profiles with neurological disability and circulating biomarkers across HCs, RRMS, and SPMS. Despite marked clinical differences between RRMS and SPMS, we found no robust phenotype-specific differences in overall microbial diversity, predefined taxonomic features, or predicted SCFA-related functional pathways. Greater interindividual microbial community dispersion was observed in SPMS in the full cohort, with a similar direction and magnitude in the age-overlap sensitivity analysis, although statistical significance was attenuated. Exploratory taxonomic differences were not corroborated by FDR-controlled analyses.

### 3.1. Clinical Phenotype and Serum Neurological Biomarkers

Despite marked differences in functional performance, circulating GFAP, NfL, and S100B did not distinguish RRMS from SPMS, consistent with a gradual rather than sharply demarcated transition to SPMS [4]. NfL predominantly reflects neuroaxonal injury and inflammatory activity, whereas GFAP may capture astroglial activation and progression-associated pathology. Although GFAP was numerically higher in SPMS, the association did not survive multiple-testing correction, supporting the complementary rather than phenotype-specific value of these biomarkers [13,15,16].

### 3.2. Microbial Diversity and Heterogeneity

Consistent with previous studies, we found no significant difference in overall beta diversity between RRMS and SPMS, suggesting that progressive MS may involve selective microbial remodeling rather than a global shift in community structure [17]. Global diversity measures may therefore be insufficiently sensitive to capture more subtle microbiome alterations associated with MS progression.

Despite the absence of centroid separation by PERMANOVA, interindividual microbial community dispersion was greater in SPMS in the full cohort. However, despite adjustment for age and sex, residual confounding by age cannot be excluded. Notably, the greater dispersion observed in SPMS was attenuated and no longer statistically significant in the age-overlap sensitivity analysis. Therefore, this finding should not be interpreted as a phenotype-specific characteristic of SPMS and requires confirmation in larger age-matched cohorts. The observed difference in dispersion may reflect multiple sources of interindividual variability rather than disease progression itself, including differences in age, treatment exposure, disease duration, diet, clinical characteristics, and pre-analytical factors such as sample storage duration. These potential biological and technical contributors cannot be fully disentangled in the present cross-sectional cohort.

Although overall REAP scores were comparable across groups, this does not exclude residual dietary confounding, as a global diet-quality score may not adequately capture microbiome-relevant exposures such as dietary fiber, resistant starch, fermented foods, or habitual dietary patterns.

Treatment exposure represents an additional potential source of microbiome variability. Previous studies have reported associations between DMT exposure and gut microbial composition in MS, although findings remain heterogeneous and treatment-specific effects are incompletely characterized [18,19]. In the present cohort, treatment exposure was strongly linked to phenotype, as all RRMS participants were treatment-naïve whereas previous or ongoing DMT exposure was common among participants with SPMS. Although the within-SPMS sensitivity analysis did not identify significant differences according to current treatment status, the cross-sectional design and heterogeneous treatment histories preclude reliable separation of treatment-associated effects from phenotype-associated differences.

Increased microbiome dispersion has previously been reported in MS, although substantial between-study heterogeneity remains [20,21,22].

### 3.3. Taxonomic Profiles

Exploratory LEfSe analyses suggested several phenotype-associated taxa. In the RRMS–SPMS comparison, *Faecalibacterium longum*, *F. prausnitzii*, *Bifidobacterium adolescentis*, and *Agathobacter faecis* showed higher relative abundance in RRMS, whereas *Ruminococcus_B gnavus* was higher in SPMS. However, these signals were not corroborated by the predefined FDR-controlled analyses and should be considered hypothesis-generating. Independent replication is required before any phenotype-specific biological significance can be inferred.

The limited differences between RRMS and SPMS suggest that the SPMS phenotype may not be characterized by a uniform restructuring of the gut microbiome as a distinct signature. Phenotype-associated microbiome differences may instead be subtle, patient-specific, or primarily functional. The greater community dispersion observed in SPMS, although attenuated in the age-overlap analysis, further suggests increased interindividual heterogeneity rather than a common SPMS-associated profile.

Importantly, the absence of FDR-significant differences should not be interpreted as evidence of biological equivalence between RRMS and SPMS, as the present sample size may have limited the detection of smaller phenotype-associated effects.

Inconsistent findings across MS microbiome studies may reflect heterogeneity, including differences in disease phenotype, treatment exposure, age, diet, sample processing, sequencing, and analytical approaches. Small cohorts and substantial interindividual microbiome variability may further limit reproducibility. Thus, previously reported taxonomic differences may represent cohort- or context-specific alterations rather than universally reproducible MS-associated signatures, highlighting the need for larger, standardized longitudinal studies.

### 3.4. SCFA and Nitric Oxide/Redox Pathways

Our prespecified functional analysis focused on six SCFA-related GMMs because of the established immunoregulatory relevance of butyrate and propionate and their proposed involvement in microbiome–host interactions in MS. We found no robust differences in predefined SCFA-related functional potential between RRMS and SPMS after correction for multiple testing. Previous studies have reported alterations in microbial metabolic profiles in MS, including changes in SCFA-related metabolism and enrichment of oxidative stress- and DNA mismatch-related genes in progressive disease [23].

The absence of robust differences in predicted SCFA-related pathways despite exploratory taxonomic variation may reflect functional redundancy, whereby different microbial configurations retain similar metabolic capabilities [24]. Importantly, metagenomic pathway abundance represents predicted microbial functional potential rather than pathway activity or actual metabolite production and therefore cannot substitute for direct fecal or circulating SCFA measurements [25]. Accordingly, the present findings do not exclude phenotype-associated differences in SCFA production, bioavailability, or host response that are not captured by metagenomic profiling alone.

The exploratory analysis of nitric oxide- and redox-related microbial functions similarly identified no robust phenotype-associated differences after multiple-testing correction. These pathways were examined because of the potential interface between microbial metabolism and oxidative and inflammatory processes; however, their metagenomic abundance cannot be interpreted as evidence of altered nitric oxide production, oxidative stress, or downstream host molecular responses. Experimental multi-omics studies have demonstrated that microbiome perturbations may coexist with changes in metabolic, transcriptional, and immune pathways, illustrating the additional information obtained by integrating microbial and host molecular measurements [26]. Such observations provide conceptual context but cannot be directly extrapolated to MS; therefore, the present NO/redox findings should be considered hypothesis-generating.

More broadly, emerging evidence suggests that microbiome involvement in MS may extend beyond broad differences in taxonomic composition or diversity and may involve microbial functions, metabolites, and host–microbiome interactions [27,28]. Similar complexity has been described in other neurodegenerative disorders, in which microbial metabolites, intestinal barrier function, immune signaling, and neuroinflammatory pathways have been implicated despite substantial between-cohort heterogeneity [29]. Thus, the absence of a robust phenotype-specific taxonomic or predicted functional signature in the present cohort does not establish biological equivalence between RRMS and SPMS.

An apparent discrepancy emerged between the marked clinical differences across MS phenotypes and the comparatively limited differences in taxonomic composition, predicted microbial functional potential, and circulating biomarkers. Several factors may account for this observation. First, metagenomically inferred pathways reflect the genetic potential of the microbial community rather than pathway activity or actual metabolite production. Functional redundancy may further allow taxonomically distinct microbial communities to maintain similar metabolic capabilities. Consequently, potentially relevant microbiome–host interactions mediated by microbial metabolites, intestinal barrier function, or immune signaling may not be captured by metagenomic functional prediction alone. Experimental and multi-omics studies support the importance of integrating microbial, metabolic, and host molecular measurements when investigating such mechanisms [30,31].

Second, circulating GFAP, NfL, and S100B reflect aspects of neuroaxonal or glial pathology rather than direct measures of intestinal microbial activity. Associations between microbial functional potential and these neurological biomarkers may therefore be indirect and mediated through microbial metabolites, intestinal barrier integrity, immune signaling, or host metabolic responses. Furthermore, the cross-sectional design captures the microbiome and circulating neurological biomarkers at a single time point, whereas neurological disability, particularly in SPMS, reflects cumulative disease processes developing over considerably longer periods. The limited cross-sectional associations observed in the present cohort therefore do not exclude microbiome–host interactions, but indicate that predicted microbial functional potential alone may be insufficient to explain contemporaneous clinical disability or circulating neurological biomarker variation.

More broadly, microbiome–host interactions extend beyond microbial composition and predicted metabolic potential and may involve intestinal barrier integrity, local and systemic immune responses, and microbial metabolites [32]. Longitudinal studies integrating metagenomic profiling with direct metabolomic measurements and repeated host biomarker assessments will be important for characterizing the temporal and mechanistic relationships between the gut microbiome and MS phenotype. Ultimately, robust functional and mechanistic validation will be required before microbiome-associated findings can inform rational microbiome-targeted therapeutic strategies [33].

### 3.5. Microbial Profiles Correlated with Neurological Disability or Serum Neurological Biomarkers

Neither predefined taxonomic features nor SCFA-related functional pathways showed robust associations with neurological disability or circulating GFAP, NfL, and S100B after multiple-testing correction. This argues against a simple cross-sectional relationship between predicted microbial functional potential and contemporaneous neurological disability or peripheral markers of neural injury.

These negative findings should also be interpreted in light of the limited statistical precision of the present cohort, particularly for sparse microbial features and phenotype interaction analyses, for which smaller or more heterogeneous associations may have remained undetected.

Previous evidence is similarly inconsistent: a recent systematic review highlighted heterogeneous associations between the microbiome and MS disease activity, course, and disability progression [10], while studies directly measuring circulating SCFAs reported associations with EDSS that did not consistently survive multiple-testing correction [34]. Evidence linking microbiome profiles to GFAP or NfL remains limited, although associations with specific microbial configurations and disability worsening have been reported [35].

The absence of significant between-group differences in circulating neurological biomarkers should also be considered. NfL primarily reflects ongoing neuroaxonal injury and is particularly sensitive to recent inflammatory disease activity; notably, the RRMS participants in our cohort were clinically stable and were not experiencing an acute relapse at the time of sampling. GFAP may more closely reflect astrocytic activation associated with progressive disease processes. GFAP levels were higher in SPMS than RRMS after adjustment for age and sex, although this association did not remain significant after correction for multiple testing. The lack of robust differences may also reflect the modest sample size, interindividual variability, and the cross-sectional assessment of biomarkers that capture contemporaneous tissue injury rather than cumulative neurological disability. S100B should be considered a complementary exploratory marker of glial injury rather than a specific marker of MS progression. Overall, these findings suggest that circulating biomarker concentrations and accumulated clinical disability may reflect partly distinct temporal and biological processes.

## 4. Materials and Methods

### 4.1. Subjects and Sample Collection

This observational cross-sectional study included 89 participants: 29 patients with RRMS, 30 with SPMS, and 30 healthy controls (HCs). All participants were adults, and MS was diagnosed according to the 2017 McDonald criteria [36]. Patients were recruited at the Neurology Department of Cluj-Napoca County Emergency Hospital, Romania, between February 2024 and August 2025.

Exclusion criteria were inflammatory or gastrointestinal disorders, pregnancy or breastfeeding, and treatment with probiotics, antivirals, antibiotics, or corticosteroids within the preceding 3 months.

All RRMS patients were treatment-naïve, whereas SPMS patients were enrolled regardless of treatment history. Treatment exposure and disease duration were also closely linked to phenotype, reflecting the intentional comparison of treatment-naïve patients with early RRMS and patients who had already transitioned to SPMS. Consequently, treatment-associated and phenotype-associated microbiome effects cannot be reliably separated in this cohort. An exploratory within-SPMS sensitivity analysis comparing currently treated and untreated participants did not identify differences in alpha diversity, overall community composition, multivariate dispersion, or predefined SCFA-related functional modules. However, this analysis does not eliminate the fundamental treatment–phenotype confounding, because all RRMS participants were treatment-naïve. Detailed information on treatment duration and exact treatment timing was not available in the analytical dataset, which further limited assessment of treatment-related effects.

Disease-modifying therapies were categorized as high-efficacy (HE) (ocrelizumab, natalizumab, siponimod, mitoxantrone) or moderate-efficacy (ME) (teriflunomide, interferon, dimethyl fumarate). The study design intentionally included treatment-naïve patients with early RRMS and patients who had transitioned to SPMS, thereby representing two clinically distinct stages of the MS disease spectrum. Because previous exposure to disease-modifying therapy is intrinsic to the clinical history of most patients reaching the secondary progressive stage, SPMS participants were enrolled irrespective of previous or ongoing MS-specific treatment.

At the time of fecal sampling, 17 of the 30 participants with SPMS were receiving disease-modifying therapy, whereas 13 were not. Among the 17 currently treated participants, the medication was recorded as siponimod in 14 and Betaferon in one, while the specific current medication was unavailable for two participants. Current treatment status was considered separately from previous DMT exposure. The relatively frequent use of siponimod in this cohort reflects current clinical practice in Romania for SPMS. Detailed information on the duration and timing of previous DMT exposure was not systematically available.

All patients were clinically stable, with no ongoing relapse at the time of sample collection. Disease duration was defined as the time from reported disease onset to study assessment and was expressed in months. Information on time since conversion to SPMS was not systematically available. Annualized relapse rate was calculated as the total number of relapses divided by the disease duration in years.

Healthy controls were volunteers recruited from the same geographic area, with no diagnosis of multiple sclerosis, and were required to meet the same study exclusion criteria regarding the medical conditions and exposures specified above. Eligibility was established based on the absence of the conditions listed in the exclusion criteria, as declared by participants. Socioeconomic matching was not specifically performed.

We collected clinical and demographic data, fecal samples, and blood samples. Neurological disability and functional performance were assessed using the Expanded Disability Status Scale (EDSS) [37], Timed 25-Foot Walk (T25FW) [38], 9-Hole Peg Test for the dominant and non-dominant hands (9HPT-D/ND) [38], and Symbol Digit Modalities Test (SDMT) [39]. One participant with SPMS did not complete the 9HPT-ND (n = 58 for this analysis).

Diet quality was assessed using the 16-item shortened Rapid Eating Assessment for Participants (REAP) questionnaire [40]. The questionnaire provided a global assessment of diet quality but did not include sufficiently detailed quantitative information on microbiome-relevant dietary exposures, such as habitual fiber or resistant starch intake and fermented food consumption.

Serum was separated from peripheral blood and stored at −20 °C until analysis. GFAP, NfL, and S100B were quantified in duplicate using commercially available ELISA kits according to the manufacturers’ instructions. Serum samples were thawed only once for biomarker analysis, with no repeated freeze–thaw cycles before measurement, analyzed in duplicate. Laboratory personnel performing the assays were blinded to participant phenotype. The analytical measurement ranges were 15.63–1000 pg/mL for GFAP (Human GFAP ELISA Kit, EEL079, Invitrogen, Thermo Fisher Scientific, Vienna, Austria), 1.6–1200 pg/mL for NfL (Human NF-L ELISA Kit, BMS2362, Invitrogen, Thermo Fisher Scientific, Vienna, Austria), and 31.25–2000 pg/mL for S100B (Human S100B ELISA Kit, EEL045, Invitrogen, Thermo Fisher Scientific, Vienna, Austria), with analytical sensitivities of 9.38, 0.8, and 18.75 pg/mL, respectively. According to the manufacturers’ specifications, the intra- and inter-assay coefficients of variation were <10% and <10%, respectively, for GFAP; 3.7% and 4.0%, respectively, for NfL; and <10% and <10%, respectively, for S100B. These analytical ranges represent assay-specific measurement intervals rather than clinical reference intervals. Values reported as zero in the analytical dataset were treated as measurements below the assay detection limit. Overall, 2/89 (2.2%) GFAP measurements, 0/89 (0%) NfL measurements, and 39/89 (43.8%) S100B measurements were below the detection limit. Because of the high proportion of non-detectable S100B measurements, S100B was analyzed as a binary detectable versus non-detectable outcome.

Fecal samples were self-collected using the CosmosID Stool Collection Kit (CosmosID Inc., Germantown, MD, USA; Cat. No. CP05407), in which stool is suspended in 2 mL of DNA/RNA Shield™ stabilization solution (Zymo Research Corp., Irvine, CA, USA). The same kit was used for all participants. Tubes were kept refrigerated at 4 °C, without freeze–thaw cycles, and shipped to the laboratory according to the manufacturer’s instructions, remaining below 25 °C throughout. DNA extraction was performed for all samples in a single batch in early February 2026. Storage duration ranged from 5.6 to 23.4 months and did not differ between groups (median 414 days, IQR 282–559 in HCs; 437 days, IQR 353–535 in RRMS; 403 days, IQR 268–533 in SPMS; Kruskal–Wallis *p* = 0.93), within the manufacturer-specified stability period for DNA in this buffer (≥2 years at ambient temperature), with independent data supporting stability for at least 18 months [41].

### 4.2. Shotgun Sequencing

Shotgun metagenomic sequencing was used for taxonomic profiling and assessment of the microbiome’s functional potential through the identification of genes and metabolic pathways, including those potentially involved in gut–brain interactions [42].

Microbial DNA was extracted using the QIAGEN DNeasy PowerSoil Pro Kit (QIAGEN, Hilden, Germany) and quantified using a Qubit Flex fluorometer (Thermo Fisher Scientific, Waltham, MA, USA). Libraries were prepared using the Watchmaker DNA Library Prep Kit (7K0019-1K; Watchmaker Genomics, Boulder, CO, USA), including DNA fragmentation, adapter ligation, seven PCR amplification cycles, and magnetic-bead purification. Libraries were quantified, circularized, and sequenced on the Element AVITI platform using 2 × 150 bp paired-end sequencing.

Raw sequencing data were processed using the CosmosID-HUB bioinformatics pipeline (https://app.cosmosid.com/, access date: 20 August 2026). Raw FASTQ files were filtered for human genomic contamination by mapping against the GRCh38.p14 human reference genome using Bowtie2 (v. 2.4.2), with read pairs discarded if either read mapped to the human genome. Adapter sequences and bases with Phred quality scores < 30 were removed using AdapterRemoval (v. 2.3.1). Following trimming and host-read removal, the remaining read pairs were defined as high-quality non-host (HQNH) reads. HQNH reads were subsequently mapped to the Clinical Microbiomics Human Microbiome Reference HMR05 gene catalog using BWA-MEM (v. 0.7.17). Reads were considered uniquely mapped when the mapping quality (MAPQ) was ≥20, sequence identity was ≥95%, and the alignment length was ≥100 bp. Species-level relative abundances were estimated from species-specific signature genes and normalized within each sample to sum to 100%. Species detection and abundance estimation followed the quality and detection criteria implemented in CosmosID-HUB, with no additional study-specific abundance threshold applied.

The HMR05 reference used in CosmosID-HUB comprises 6567 prokaryotic species clusters and 244 eukaryotic species, with a final gene catalog containing 25,761,278 genes. Taxonomic annotation of the underlying metagenome-assembled genomes was based on GTDB-Tk (v. 2.3.0) using GTDB release r214. For species quantification, up to 250 species-specific signature genes were selected, preferentially from core genes present in ≥60% of species MAGs, with lengths between 200 bp and 20 kbp and species-uniqueness criteria applied within the HMR05 catalog. Species abundance estimation required evidence from multiple signature genes; species abundances were set to zero when fewer than five genes with non-zero read counts fell within the expected 99% count quantile. Additional detection and abundance-estimation criteria followed the standard HMR05/CosmosID-HUB workflow. Taxonomic nomenclature follows the GTDB-based classification implemented in the HMR05 reference catalog. Accordingly, GTDB genus designations containing alphabetical suffixes (e.g., *Ruminococcus_B*) were retained throughout the manuscript to ensure consistency with the taxonomic output used for the analyses.

Gut Metabolic Modules (GMMs), defined as conserved metabolic pathways represented by Kyoto Encyclopedia of Genes and Genomes orthology (KO) identifiers [43], together with Gut–Brain Modules (GBMs), were used to characterize predicted microbial functional potential. The principal functional analysis was hypothesis-driven and focused on six prespecified SCFA-related GMMs, selected because of the biological relevance of butyrate and propionate to intestinal homeostasis and immune regulation and their previously reported involvement in MS. These comprised two butyrate-production pathways (MF0088 and MF0089), three propionate-production pathways (MF0093, MF0094, and MF0095), and succinate conversion to propionate (MF0084). These modules were prespecified before examination of the current dataset and were selected to provide a focused assessment of SCFA-related functional potential rather than a comprehensive evaluation of all potentially relevant microbiome-derived metabolites. Other microbial metabolic functions were not included in the principal hypothesis-driven analysis because they were outside the prespecified SCFA-focused hypothesis. To provide broader functional context without redefining the principal analysis after data inspection, the available GMM and GBM profiles were subsequently evaluated in separate exploratory, multiple-testing-controlled analyses.

In addition to the prespecified SCFA-focused analysis, six microbial functions related to nitric oxide metabolism and redox homeostasis were evaluated in a separate exploratory analysis: nitric oxide synthesis I (MGB025), nitric oxide synthesis II (MGB026), nitric oxide degradation I (MGB027), nitric oxide degradation II (MGB028), the oxidative phase of the pentose phosphate pathway (MF0070), and NADH:ferredoxin oxidoreductase (MF0069). These functions were selected to provide an exploratory assessment of microbial pathways potentially related to nitric oxide turnover and cellular redox metabolism, given the broader relevance of nitrosative and oxidative stress to inflammatory and neurodegenerative processes in MS. The nitric oxide synthesis and degradation modules were included to capture microbial functional potential related to nitric oxide turnover, whereas the oxidative pentose phosphate pathway and NADH:ferredoxin oxidoreductase were included as redox-related functions involved in cellular reducing-equivalent and electron-transfer processes. This analysis was exploratory rather than part of the prespecified primary functional analysis, and its findings were therefore interpreted as hypothesis-generating.

### 4.3. Statistical Analysis

All statistical analyses were performed in R 4.6.1. Continuous variables were summarized as median (interquartile range) and categorical variables as n (%). Overall comparisons across HCs, RRMS, and SPMS used Kruskal–Wallis or Fisher’s exact tests, with pairwise Wilcoxon rank-sum tests and Holm adjustment where appropriate. The age- and sex-adjusted RRMS–SPMS comparison was considered the principal disease phenotype analysis.

Circulating neurological biomarkers were analyzed using linear regression for GFAP, log-linear regression for NfL, and logistic regression for detectable versus non-detectable S100B. Continuous-outcome models were additionally evaluated using HC3 heteroscedasticity-consistent standard errors. Alpha diversity (Shannon and Simpson indices) was compared using non-parametric tests, while Bray–Curtis beta diversity and multivariate dispersion were assessed using PERMANOVA and PERMDISP, respectively. Adjusted PERMANOVA models included age and sex, and PERMDISP pairwise comparisons used Tukey adjustment. Principal RRMS–SPMS analyses were repeated within the common age range (32–57 years) as a sensitivity analysis. Among participants with MS, associations of Shannon and Simpson diversity with neurological disability and circulating biomarkers were additionally evaluated using models adjusted for phenotype, age, and sex. Diversity indices were standardized before modeling. Outcome-specific modeling followed the same framework used for functional associations. Spearman correlations were additionally calculated as descriptive unadjusted measures of association. FDR correction was applied across the 14 adjusted diversity–outcome associations, with descriptive Spearman correlations corrected separately.

To explore potential treatment-associated microbiome differences within the SPMS group, an exploratory sensitivity analysis compared participants receiving disease-modifying therapy at the time of fecal sampling with those not receiving treatment. Shannon and Simpson alpha diversity were compared using Wilcoxon rank-sum tests. Bray–Curtis community composition was evaluated using PERMANOVA, including an age- and sex-adjusted model, and multivariate dispersion was assessed using PERMDISP. The six predefined SCFA-related functional modules were compared using Wilcoxon rank-sum tests for continuous modules and Fisher’s exact tests for detection outcomes, with Benjamini–Hochberg correction across the six functional comparisons. A high- versus moderate-efficacy treatment comparison was not performed because only one participant with a documented current medication was receiving a moderate-efficacy therapy.

Six predefined SCFA-related GMMs were analyzed as continuous variables (butyrate production I and II and propionate production II) or detection outcomes (propionate production I and III and succinate conversion to propionate), according to their distribution. Continuous and binary pathways were compared using Kruskal–Wallis/Wilcoxon and Fisher’s exact tests, respectively, with Hodges–Lehmann location shifts or odds ratios and 95% CIs reported as effect estimates. Adjusted analyses used age- and sex-adjusted log-linear or logistic regression, respectively.

As an exploratory whole-profile functional analysis, all available GMM and GBM profiles were additionally evaluated. Modules detected in ≥80% of participants were analyzed as abundance measures after log1p transformation using age- and sex-adjusted linear regression with HC3 heteroscedasticity-consistent standard errors. Modules detected in 20% to <80% of participants were analyzed as binary detection outcomes using age- and sex-adjusted logistic regression, whereas modules detected in <20% of participants were excluded from adjusted inference because of sparse detection. Benjamini–Hochberg FDR correction was applied separately to GMMs and GBMs within each pairwise contrast (RRMS vs. HCs, SPMS vs. HCs, and SPMS vs. RRMS).

A predefined panel of 17 taxonomic features was selected independently of the results of the present dataset, based on previously reported associations with MS and/or biological relevance to microbiome–host interactions, including intestinal homeostasis, immune regulation, and SCFA-related microbial functions. The panel comprised two phyla (Bacillota and Bacteroidota), two families (Lachnospiraceae and Enterobacteriaceae), and 13 genera (*Bifidobacterium*, *Roseburia*, *Coprococcus*, *Butyricicoccus*, *Lachnospira*, *Dorea*, *Faecalibacterium*, *Prevotella*, *Akkermansia*, *Blautia*, *Ruminococcus_B*, *Agathobaculum*, and *Alistipes*). Selection of these features preceded examination of the between-group taxonomic results in the present cohort and was therefore considered hypothesis-driven. This predefined panel was analyzed separately from the subsequent exploratory species-level LEfSe analysis. The literature-based rationale and supporting references for the predefined panel are provided [8,23,44].

Seventeen predefined taxonomic features were analyzed using age- and sex-adjusted ANCOM-BC2 models on count data in a prespecified hierarchical analysis at the phylum, family, or genus level, including aggregation of GTDB suffix variants belonging to the same genus. *Butyricicoccus* was primarily analyzed as a detection outcome because of low prevalence, while *Akkermansia* detection was assessed as a sensitivity analysis. Pseudo-count sensitivity analyses assessed robustness to zero handling.

Associations of SCFA-related functions and predefined taxa with disability and circulating biomarkers were evaluated among patients with MS using models adjusted for age, sex, and phenotype. Continuous GMMs were log-transformed and standardized, whereas sparse modules were analyzed as detection variables. EDSS was analyzed using cumulative logit regression, with linear regression and HC3 standard errors as a sensitivity analysis; T25FW and 9HPT were log-transformed and analyzed using linear regression. Taxonomic associations were evaluated using ANCOM-BC2, with pseudo-count sensitivity analyses. Predictor-by-phenotype interactions assessed differences between RRMS and SPMS and were FDR-corrected separately.

Six additional nitric oxide/redox-related functions were explored using the same framework as the GMM analyses. Five had sufficient prevalence for adjusted analyses, whereas nitric oxide degradation II was analyzed only by detection because it was detected in four participants. Species-level LEfSe was performed as an exploratory analysis for pairwise group comparisons using nominal *p* < 0.05 and an LDA score threshold of 2.0.

All tests were two-sided. Benjamini–Hochberg FDR correction was applied separately within prespecified hypothesis families. Given the exploratory design and fixed sample size, inference emphasized effect estimates, 95% CIs, and multiplicity-adjusted results rather than post hoc power calculations.

Throughout the manuscript, *p* values denote nominal (unadjusted) probabilities, whereas *q*-values denote FDR-adjusted probabilities. Statistical significance for analyses subject to multiple-testing correction was defined as *q* < 0.05; findings with nominal *p* < 0.05 but *q* ≥ 0.05 were considered exploratory and were not interpreted as statistically significant after multiple-testing correction.

## 5. Conclusions

We identified no robust RRMS–SPMS differences in predefined taxonomic features or SCFA-related functional potential after multiple-testing correction. Greater microbial community dispersion was observed in SPMS in the full cohort, although this difference was attenuated in the age-overlap sensitivity analysis, while exploratory taxonomic findings require independent validation. Overall, our findings do not support a distinct SPMS microbiome signature and indicate limited robust differences in gut microbial composition and predicted functional potential between RRMS and SPMS. Given the cross-sectional design and the substantial differences in age and disease-modifying treatment exposure between RRMS and SPMS, these observations should be interpreted cautiously as phenotype-associated differences rather than within-patient changes occurring during disease progression.

Longitudinal studies following individuals across the RRMS-to-SPMS transition and integrating metagenomics with direct metabolomics and host molecular profiling are needed to determine whether and how the gut microbiome changes during MS progression.

### Strengths and Limitations

Strengths of this study include high-resolution taxonomic and functional profiling and a rigorous analytical framework incorporating multiple-testing correction, adjustment for age and sex, and sensitivity analyses.

Several limitations should also be acknowledged.

First, the substantial age difference between RRMS and SPMS represents an important potential confounder; although age was considered in adjusted analyses and an age-overlap sensitivity analysis was performed, residual age-related effects cannot be excluded, particularly given the reduced sample size of the sensitivity analysis. Second, treatment exposure was strongly linked to phenotype, as all RRMS participants were treatment-naïve whereas SPMS participants had heterogeneous current and previous DMT exposure, precluding reliable separation of treatment-related effects from phenotype-associated differences. Disease duration was also substantially longer in SPMS and closely related to phenotype, age, accumulated disability, and treatment exposure. Dietary assessment relied on the shortened REAP questionnaire, which provides a global measure of diet quality but may not capture specific microbiome-relevant dietary exposures; therefore, residual dietary confounding cannot be excluded. Third, the cross-sectional design does not allow the observed RRMS–SPMS differences to be interpreted as within-patient microbiome changes occurring during disease progression. Fourth, although all fecal samples were collected using the same stabilization protocol and storage duration did not differ significantly among groups, samples were stored for variable periods before DNA extraction, and a residual pre-analytical effect cannot be completely excluded. Fifth, the overall cohort and particularly subgroup and sensitivity analyses were of modest size, limiting statistical precision and the ability to detect smaller phenotype-associated effects, particularly for sparse microbial features and interaction analyses. Finally, the functional analyses reflect predicted microbial metabolic potential derived from metagenomic data rather than direct measurement of microbial activity or metabolite concentrations. The absence of direct metabolomic measurements therefore prevents conclusions regarding actual SCFA production, nitric oxide/redox activity, or their downstream host effects. Collectively, these limitations constrain causal and phenotype-specific interpretation and may limit the generalizability of our findings. Larger, age-matched longitudinal cohorts integrating metagenomics with direct metabolomics and host molecular profiling will be required to determine whether microbiome alterations accompany the transition from RRMS to SPMS and to clarify their potential biological significance.

## Figures and Tables

**Table 1:** Clinical performance test results in the study population.

Characteristic	HC (*n* = 30)	RRMS (*n* = 29)	SPMS (*n* = 30)	*p*	HC vs. RRMS	HC vs. SPMS	RRMS vs. SPMS
EDSS	0.0 (0.0–0.0)	1.5 (1.0–2.0)	6.0 (4.5–6.5)	<0.001	<0.001	<0.001	<0.001
Disease duration, months, median (IQR)	NA	17 (6–57)	204 (147–284)	<0.001	NA	NA	<0.001
Annualized relapse rate, median (IQR)	NA	1.00 (0.40–1.00)	0.30 (0.19–0.49)	<0.001	NA	NA	<0.001
T25FW, s	5.26 (4.37–6.23)	5.91 (4.86–6.95)	11.72 (8.76–17.84)	<0.001	0.156	<0.001	<0.001
9HPT, dominant hand, s	17.87 (16.95–19.22)	22.10 (17.85–24.26)	33.17 (25.50–43.60)	<0.001	0.004	<0.001	<0.001
9HPT, non-dominant hand, s	19.80 (17.55–20.59)	22.60 (19.40–24.90)	32.90 (28.43–39.40)	<0.001	0.001	<0.001	<0.001
SDMT	74.0 (52.2–92.2)	58.0 (51.0–76.0)	30.5 (20.0–44.8)	<0.001	0.120	<0.001	<0.001

Values are presented as median (IQR). Overall comparisons were performed using the Kruskal–Wallis test. Pairwise comparisons were performed using Wilcoxon rank-sum tests with Holm adjustment for the three pairwise comparisons within each outcome. NA, not applicable.

**Table 2:** Predefined SCFA-related Gut Metabolic Modules across the study population.

Predicted GMM Pathway	HC (*n* = 30)	RRMS (*n* = 29)	SPMS (*n* = 30)	Overall *p*	FDR-Adjusted *q*	HC vs. RRMS, Holm-Adjusted *p*	HC vs. SPMS, Holm-Adjusted *p*	RRMS vs. SPMS, Holm-Adjusted *p*
**Butyrate production I**	6.68 (5.22)	7.41 (4.60)	8.78 (4.05)	0.147	0.220	0.767	0.247	0.247
**Butyrate production II**	3.82 (4.72)	4.05 (5.33)	6.24 (3.98)	0.256	0.263	0.622	0.326	0.557
**Propionate production II**	2.40 (1.08)	3.38 (1.59)	3.36 (2.57)	0.094	0.188	0.295	0.135	0.336
**Propionate production I, detected**	6/30 (20.0%)	10/29 (34.5%)	14/30 (46.7%)	0.094	0.188	0.503	0.162	0.503
**Propionate production III, detected**	9/30 (30.0%)	12/29 (41.4%)	19/30 (63.3%)	0.032	0.188	0.422	0.057	0.241
**Succinate conversion to propionate, detected**	5/30 (16.7%)	10/29 (34.5%)	9/30 (30.0%)	0.263	0.263	0.429	0.721	0.785

Overall comparisons were performed using the Kruskal–Wallis test for continuously distributed modules and Fisher’s exact test for sparsely detected modules. Pairwise comparisons were performed using Wilcoxon rank-sum tests for continuous modules and Fisher’s exact tests for detection outcomes, with Holm adjustment across the three pairwise comparisons within each module.

**Table 3:** Direct comparison of predefined SCFA-related pathways between RRMS and SPMS.

Predicted GMM Pathway	RRMS	SPMS	Effect Estimate, SPMS vs. RRMS	95% CI	Nominal *p*	FDR-Adjusted *q*
**Butyrate production I**	7.41 (4.60)	8.78 (4.05)	HL shift +1.57	−0.47 to 3.72	0.110	0.361
**Butyrate production II**	4.05 (5.33)	6.24 (3.98)	HL shift +1.04	−0.67 to 2.99	0.278	0.503
**Propionate production II**	3.38 (1.59)	3.36 (2.57)	HL shift +0.40	−0.39 to 1.28	0.336	0.503
**Propionate production I, detected**	10/29 (34.5%)	14/30 (46.7%)	OR 1.65	0.52–5.43	0.430	0.515
**Propionate production III, detected**	12/29 (41.4%)	19/30 (63.3%)	OR 2.41	0.76–7.96	0.120	0.361
**Succinate conversion to propionate, detected**	10/29 (34.5%)	9/30 (30.0%)	OR 0.82	0.24–2.79	0.785	0.785

Continuous pathways are presented as median (IQR). Direct RRMS–SPMS comparisons were performed using the Wilcoxon rank-sum test, and effect estimates are Hodges–Lehmann location shifts for SPMS relative to RRMS with 95% confidence intervals. Pathways with frequent absence of detectable signal are presented as n/N (%) detected and were compared using Fisher’s exact test; effect estimates are odds ratios for pathway detection in SPMS relative to RRMS. *q*-values were obtained using the Benjamini–Hochberg procedure across the six predefined RRMS–SPMS comparisons.

## Data Availability

The original contributions presented in this study are included in the article/Supplementary Materials. Further inquiries can be directed to the corresponding author.

## Supplementary Materials

The following supporting information can be downloaded at: https://www.mdpi.com/article/10.3390/ijms27209166/s1.
