# Supplementary material for: Limited Gut Microbiome Differences Between Relapsing–Remitting and Secondary Progressive Multiple Sclerosis: A Shotgun Metagenomic Study
Source: Int J Mol Sci. 2026 Oct 16;27(20):9166. doi: 10.3390/ijms27209166 (PMC13705247; doi:10.3390/ijms27209166)
Supplement: Supplementary file 1 [file ijms-27-09166-s001.zip › ijms-4613320-supplementary.pdf]

SUPPLEMENTARY MATERIAL

Notes: Values are reproduced from the supplied statistical workbook and rounded for display. p- and q-values <0.001 are shown as <0.001. An em dash indicates that a statistic was not applicable or not available for that model.

Abbreviations: EDSS, Expanded Disability Status Scale; FDR, false discovery rate; GBM, gut-brain module; GFAP, glial fibrillary acidic protein; GMM, gut metabolic module; HC3, heteroscedasticity-consistent type 3 standard errors; LFC, log fold change; NfL, neurofilament light chain; OR, odds ratio; RRMS, relapsing-remitting multiple sclerosis; SPMS, secondary progressive multiple sclerosis; T25FW, Timed 25-Foot Walk; 9HPT, 9-Hole Peg Test.

Table S1. Demographic and lifestyle characteristics of the study population

Characteristic	HC (n=30)	RRMS (n=29)	SPMS (n=30)	Overall p	HC vs RRMS	HC vs SPMS	RRMS vs SPMS
Age, years	29.0 (27.2–45.8)	31.0 (27.0–48.0)	49.0 (41.0–56.8)	**<0.001**	0.796	<0.001	<0.001
Female sex	17 (56.7%)	18 (62.1%)	18 (60.0%)	0.963	1.000	1.000	1.000
Smoking, yes	3 (10.0%)	7 (24.1%)	11 (36.7%)	0.052	0.361	0.091	0.399
Alcohol consumption, yes	14 (46.7%)	12 (41.4%)	11 (36.7%)	0.742	1.000	1.000	1.000
Dietary restrictions, yes	2 (6.7%)	5 (17.2%)	2 (6.7%)	0.415	0.763	1.000	0.763
REAP total score	26.0 (22.2–28.8)	27.0 (25.0–29.0)	25.5 (23.0–29.0)	0.668	1.000	1.000	1.000

**Continuous variables are presented as median (IQR) and categorical variables as n (%). Overall comparisons were performed using the Kruskal–Wallis test for continuous variables and Fisher's exact test for categorical variables. Pairwise comparisons were performed using Wilcoxon rank-sum tests for continuous variables and Fisher's exact tests for categorical variables, with Holm adjustment across the three pairwise comparisons within each characteristic. Abbreviations: HC, healthy controls; RRMS, relapsing-remitting multiple sclerosis; SPMS, secondary progressive multiple sclerosis; REAP, Rapid Eating Assessment for Participants.*

Table S2a. Serum biomarkers in study population

Biomarker (pg/mL)	HC (n=30)	RRMS (n=29)	SPMS (n=30)	Overall p	HC vs RRMS	HC vs SPMS	RRMS vs SPMS
GFAP	102.90 (55.32–138.35)	79.74 (29.44–124.80)	110.00 (72.37–152.88)	0.146	0.308	0.679	0.183
NfL	25.00 (22.30–33.41)	26.12 (21.78–30.05)	27.83 (23.64–33.45)	0.464	0.888	0.783	0.888
S100B	16.72 (0.00–24.26)	0.00 (0.00–21.72)	7.95 (0.00–21.81)	0.359	0.489	0.687	0.656

**Values are presented as median (IQR). Overall comparisons were performed using the Kruskal–Wallis test. Pairwise comparisons were performed using Wilcoxon rank-sum tests with Holm adjustment across the three pairwise comparisons within each biomarker.*
Measurements below the detection limit were observed for GFAP in 1/30(3.3%) HC, 1/29 (3.4%) RRMS, and 0/30 (0%) SPMS participants; for NfL in 0/30, 0/29, and 0/30 participants, respectively; and for S100B in 11/30 (36.7%), 16/29 (55.2%), and 12/30 (40.0%), respectively.

Table S2b. Age- and sex-adjusted comparison of circulating biomarkers between RRMS and SPMS

Biomarker	Adjusted effect, SPMS vs RRMS	95% CI	*p*	*q*
GFAP	Mean difference +37.66	0.45 to 74.88	0.047	0.124
NfL	Ratio 1.00	0.73–1.37	0.995	0.995
S100B detection	OR 3.20	0.89–12.79	0.083	0.124

**Models were adjusted for age and sex, with RRMS as the reference group. GFAP was analysed using linear regression on the original scale; NfL was natural-log transformed and the exponentiated coefficient is reported as the SPMS/RRMS ratio. Because of the high frequency of zero values, S100B was analysed as detectable versus non-detectable using logistic regression. q-values were obtained using the Benjamini–Hochberg procedure across the three predefined biomarkers.*

Table S3a. Raw read pairs from the CHAMP (CosmosID) sample reports

Sample ID	Group	Raw read pairs (millions)	Read pairs after host removal and QC (millions)	Retained (%)
200175	HC	18.026	18.011	99.9
200184	HC	7.889	7.885	99.9
200192	HC	13.621	13.606	99.9
200198	HC	17.96	17.756	98.9
200208	HC	10.803	10.506	97.2
200218	HC	10.107	9.998	98.9
200221	HC	21.974	21.908	99.7
200225	HC	11.005	10.641	96.7
200229	HC	29.735	29.249	98.4
200230	HC	14.482	14.451	99.8
200232	HC	20.331	20.306	99.9
200234	HC	11.084	11.004	99.3
200236	HC	15.296	15.243	99.7
200240	HC	13.087	13.034	99.6
200245	HC	7.052	7.045	99.9
200252	HC	13.558	13.442	99.1
200253	HC	7.98	7.968	99.9
200254	HC	11.048	11.026	99.8
259081	HC	14.168	14.119	99.7

259093	HC	28.822	28.806	99.9
259102	HC	14.296	14.204	99.4
259108	HC	7.568	7.56	99.9
259109	HC	15.694	15.683	99.9
259110	HC	15.783	15.777	100
259113	HC	21.227	21.206	99.9
259114	HC	10.594	10.588	99.9
259115	HC	16.899	16.886	99.9
259116	HC	14.963	14.946	99.9
259117	HC	13.133	13.02	99.1
259118	HC	20.25	20.238	99.9
200165	RRMS	10.9	10.858	99.6
200166	RRMS	14.272	13.889	97.3
200173	RRMS	14.551	14.541	99.9
200174	RRMS	21.892	21.875	99.9
200177	RRMS	11.37	11.332	99.7
200180	RRMS	15.145	14.551	96.1
200183	RRMS	14.68	14.668	99.9
200188	RRMS	17.683	17.631	99.7
200189	RRMS	10.386	10.372	99.9
200190	RRMS	16.559	16.524	99.8
200194	RRMS	19.227	19.216	99.9
200195	RRMS	11.95	11.928	99.8
200200	RRMS	15.698	15.686	99.9
200201	RRMS	7.541	7.526	99.8
200202	RRMS	18.22	18.082	99.2
200203	RRMS	13.209	13.198	99.9
200204	RRMS	10.636	10.617	99.8
200206	RRMS	18.223	18.212	99.9
200210	RRMS	9.47	9.466	100

200211	RRMS	16.471	16.452	99.9
200213	RRMS	11.289	11.24	99.6
200214	RRMS	17.401	17.391	99.9
200215	RRMS	10.726	10.72	99.9
200216	RRMS	21.358	21.35	100
200220	RRMS	10.67	10.653	99.8
200237	RRMS	8.297	8.229	99.2
200239	RRMS	11.81	11.722	99.3
200248	RRMS	10.943	10.927	99.9
200249	RRMS	9.197	9.186	99.9
200167	SPMS	16.638	16.537	99.4
200168	SPMS	11.172	11.161	99.9
200169	SPMS	12.136	12.116	99.8
200170	SPMS	15.62	15.609	99.9
200171	SPMS	10.353	10.33	99.8
200172	SPMS	19.479	19.334	99.3
200176	SPMS	22.311	22.295	99.9
200178	SPMS	8.847	8.296	93.8
200179	SPMS	10.1	10.082	99.8
200181	SPMS	18.294	18.274	99.9
200182	SPMS	11.441	11.394	99.6
200185	SPMS	9.06	9.023	99.6
200187	SPMS	9.268	9.242	99.7
200191	SPMS	13.417	13.374	99.7
200196	SPMS	12.822	12.805	99.9
200197	SPMS	10.775	10.691	99.2
200205	SPMS	10.905	10.861	99.6
200207	SPMS	13.861	13.842	99.9
200209	SPMS	19.049	19.016	99.8
200212	SPMS	12.496	12.473	99.8

200217	SPMS	16.789	16.739	99.7
200219	SPMS	11.766	11.761	100
200226	SPMS	15.331	15.156	98.9
200231	SPMS	11.11	11.072	99.7
200235	SPMS	16.515	16.202	98.1
200238	SPMS	11.824	11.808	99.9
200241	SPMS	13.024	12.994	99.8
200243	SPMS	10.592	10.571	99.8
200244	SPMS	17.779	17.719	99.7
200247	SPMS	11.963	11.842	99

Read pairs after host removal and QC counted in the host-depleted, quality-filtered FASTQ files. HC, healthy controls; RRMS, relapsing-remitting MS; SPMS, secondary progressive MS.

Table S3b. Raw read pairs from the CHAMP (CosmosID) group report

Group	n	Raw read pairs, millions, median (IQR)	Read pairs after host removal and QC, millions, median (IQR)
HC	30	14.2 (11.0–17.7)	14.2 (10.7–17.5)
RRMS	29	13.2 (10.7–16.6)	13.2 (10.7–16.5)
SPMS	30	12.3 (11.0–16.3)	12.3 (10.9–16.1)
All	89	13.2 (10.9–16.6)	13.2 (10.7–16.5)
Kruskal–Wallis p		0.69	0.73

Figure S1. Alpha diversity across study population.

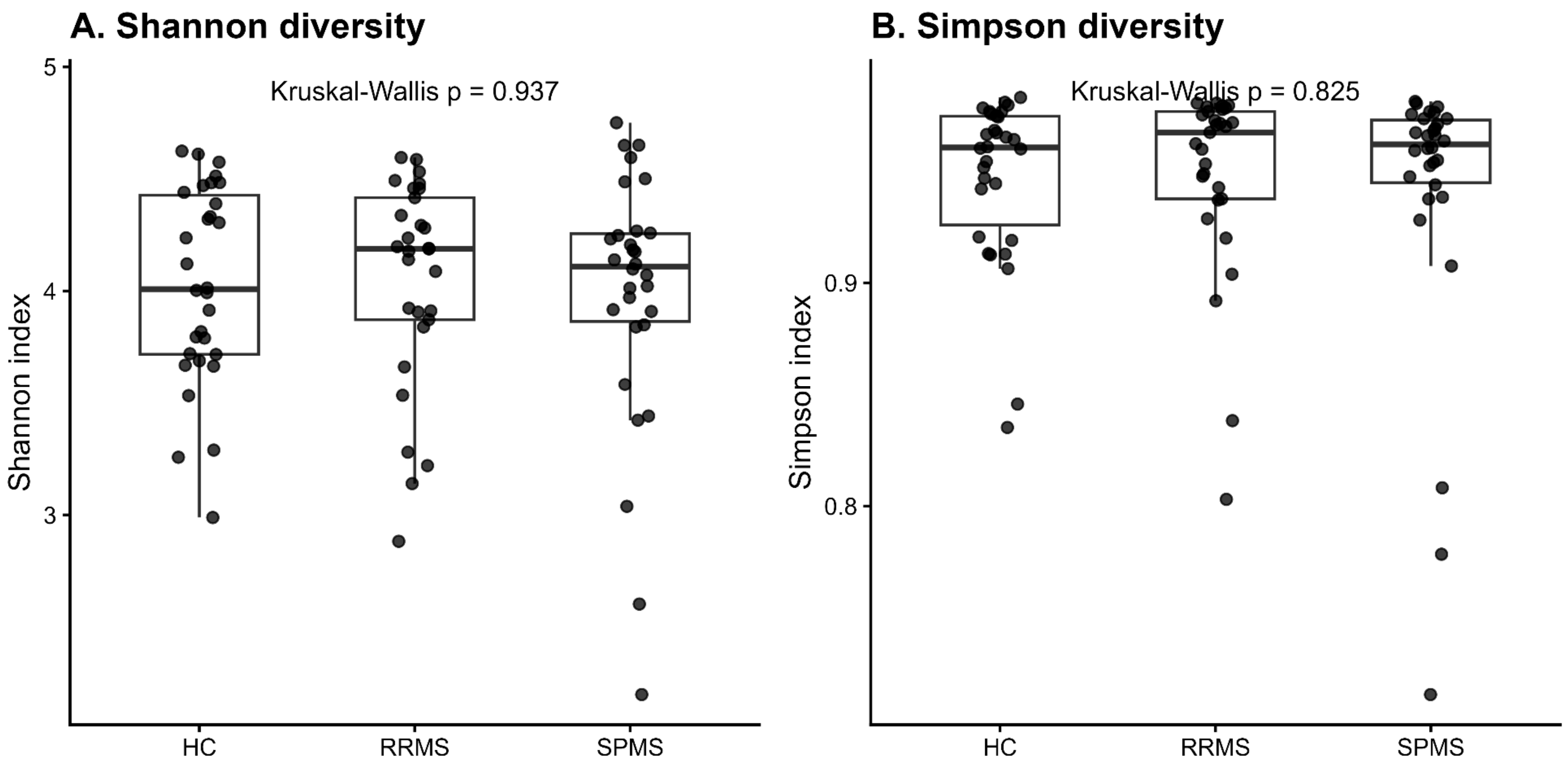

Species-level alpha diversity was assessed using the Shannon (A) and Simpson (B) indices. Individual points represent participants, and boxplots show the median and interquartile range. No differences were observed among HC, RRMS, and SPMS for either Shannon diversity (Kruskal–Wallis p=0.937) or Simpson diversity (p=0.825).

Figure S2. Principal coordinates analysis of gut microbial beta diversity across study population.

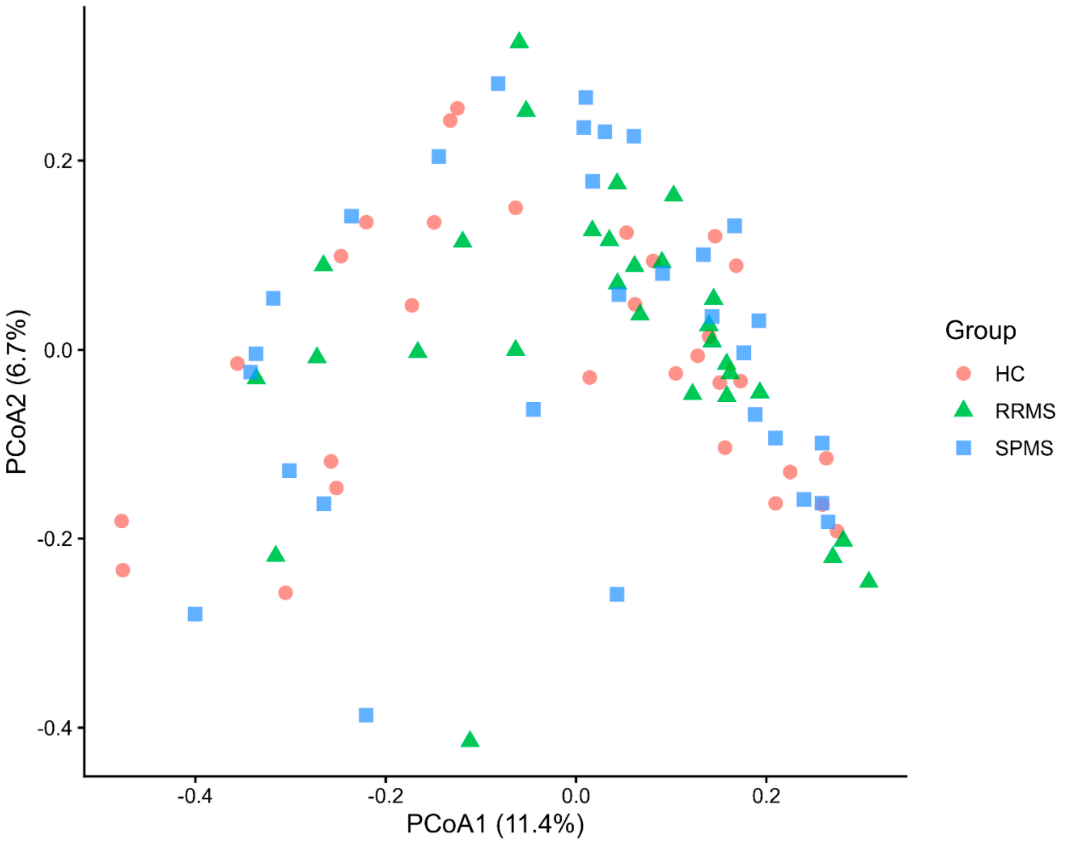

Principal coordinates analysis (PCoA) was performed using species-level Bray–Curtis dissimilarities. Each point represents one participant. No significant separation in overall microbial community composition was observed among HC, RRMS, and SPMS (PERMANOVA R^2=0.025, p=0.208); the group effect remained non-significant after adjustment for age and sex (R^2=0.021, p=0.635)

Table S4. Associations of alpha diversity with neurological disability and circulating biomarkers among participants with MS

Diversity index	Outcome	N	Spearman rho	Spearman p	Spearman q	Adjusted effect	Estimate	95% CI	p	q
Shannon	EDSS	59	0.144	0.278	0.780	Proportional-odds OR per 1-SD diversity	1.683	0.976 to 2.905	0.061	0.257
Shannon	T25FW	59	0.030	0.821	0.886	Completion-time ratio per 1-SD diversity	1.032	0.929 to 1.146	0.554	0.862
Shannon	9HPT-D	59	0.182	0.169	0.780	Completion-time ratio per 1-SD diversity	1.075	0.905 to 1.277	0.410	0.718
Shannon	9HPT-ND	58	0.165	0.215	0.780	Completion-time ratio per 1-SD diversity	1.135	1.017 to 1.267	0.024	0.257
Shannon	GFAP	59	0.030	0.822	0.886	Mean difference per 1-SD diversity	2.537	-19.159 to 24.232	0.819	0.882
Shannon	NfL	59	0.085	0.523	0.886	Ratio per 1-SD diversity	1.068	0.953 to 1.196	0.258	0.602
Shannon	S100B detection	59	-0.082	0.538	0.886	OR for detection per 1-SD diversity	0.906	0.529 to 1.552	0.719	0.882
Simpson	EDSS	59	0.143	0.278	0.780	Proportional-odds OR per 1-SD diversity	1.899	1.018 to 3.544	0.044	0.257
Simpson	T25FW	59	0.056	0.673	0.886	Completion-time ratio per 1-SD diversity	1.080	0.979 to 1.191	0.125	0.351
Simpson	9HPT-D	59	0.177	0.179	0.780	Completion-time ratio per 1-SD diversity	1.058	0.834 to 1.343	0.642	0.882
Simpson	9HPT-ND	58	0.120	0.368	0.859	Completion-time ratio per 1-SD diversity	1.136	0.988 to 1.305	0.074	0.257
Simpson	GFAP	59	-0.039	0.769	0.886	Mean difference per 1-SD diversity	-0.273	-26.453 to 25.908	0.984	0.984
Simpson	NfL	59	0.011	0.936	0.936	Ratio per 1-SD diversity	1.044	0.945 to 1.155	0.396	0.718
Simpson	S100B detection	59	-0.066	0.621	0.886	OR for detection per 1-SD diversity	0.922	0.535 to 1.590	0.770	0.882

Analyses were restricted to participants with MS. Shannon and Simpson indices were standardized, and adjusted effect estimates correspond to a 1-SD increase in the diversity index. Models were adjusted for phenotype, age, and sex. EDSS was analyzed using proportional-odds regression; T25FW and 9HPT completion times and NfL were natural-log transformed; GFAP was analyzed on the original scale; and S100B was analyzed as detectable versus non-detectable. HC3 heteroscedasticity-consistent standard errors were used for continuous-outcome models. Spearman correlations are presented as descriptive unadjusted analyses. Benjamini-Hochberg FDR correction was applied across the 14 adjusted diversity-outcome associations; descriptive Spearman correlations were corrected separately across the same 14 comparisons.

Table S5. Age- and sex-adjusted pairwise comparisons of predefined taxonomic features

Taxonomic level	Taxon	Contrast	Effect type	Estimate	95% CI	p	q	Pseudo-count sensitivity
Phylum	Bacillota	RRMS vs HC	LFC	-0.081	-0.459 to 0.298	0.676	0.788	Passed
Phylum	Bacteroidota	RRMS vs HC	LFC	-0.230	-0.681 to 0.221	0.320	0.788	Passed
Family	Lachnospiraceae	RRMS vs HC	LFC	-0.169	-0.639 to 0.300	0.482	0.788	Passed
Family	Enterobacteriaceae	RRMS vs HC	LFC	-0.216	-1.216 to 0.784	0.674	0.788	Passed
Genus	*Bifidobacterium*	RRMS vs HC	LFC	-0.393	-1.293 to 0.507	0.395	0.788	Passed
Genus	*Roseburia*	RRMS vs HC	LFC	-0.000	-0.741 to 0.741	0.999	0.999	Passed
Genus	*Coprococcus*	RRMS vs HC	LFC	-0.137	-0.971 to 0.697	0.748	0.795	Passed
Genus	*Butyricicoccus*	RRMS vs HC	OR for detection	0.266	0.081 to 0.873	0.029	0.494	Not applicable (detection model)
Genus	*Lachnospira*	RRMS vs HC	LFC	-0.154	-0.745 to 0.437	0.612	0.788	Passed
Genus	*Dorea*	RRMS vs HC	LFC	-0.300	-0.817 to 0.218	0.260	0.788	Passed

Taxonomic level	Taxon	Contrast	Effect type	Estimate	95% CI	p	q	Pseudo-count sensitivity
Genus	*Faecalibacterium*	RRMS vs HC	LFC	-0.127	-0.671 to 0.417	0.649	0.788	Passed
Genus	*Prevotella*	RRMS vs HC	LFC	-0.371	-2.218 to 1.476	0.695	0.788	Passed
Genus	*Akkermansia*	RRMS vs HC	LFC	-0.609	-1.445 to 0.226	0.161	0.788	Passed
Genus	*Blautia*	RRMS vs HC	LFC	-0.260	-0.774 to 0.254	0.325	0.788	Passed
Genus	*Ruminococcus*	RRMS vs HC	LFC	-0.156	-0.896 to 0.584	0.680	0.788	Passed
Genus	*Agathobaculum*	RRMS vs HC	LFC	-0.236	-0.915 to 0.443	0.497	0.788	Passed
Genus	*Alistipes*	RRMS vs HC	LFC	0.220	-0.447 to 0.887	0.520	0.788	Passed
Phylum	Bacillota	SPMS vs HC	LFC	-0.265	-0.745 to 0.215	0.282	0.441	Passed
Phylum	Bacteroidota	SPMS vs HC	LFC	-0.224	-0.705 to 0.257	0.363	0.441	Passed
Family	Lachnospiraceae	SPMS vs HC	LFC	-0.366	-0.916 to 0.184	0.196	0.441	Passed
Family	Enterobacteriaceae	SPMS vs HC	LFC	0.163	-1.091 to 1.417	0.800	0.850	Passed
Genus	*Bifidobacterium*	SPMS vs HC	LFC	-0.992	-2.020 to 0.037	0.062	0.441	Passed
Genus	*Roseburia*	SPMS vs HC	LFC	-0.267	-1.205 to 0.672	0.579	0.657	Passed
Genus	*Coprococcus*	SPMS vs HC	LFC	-0.651	-1.725 to 0.424	0.239	0.441	Passed
Genus	*Butyricicoccus*	SPMS vs HC	OR for detection	0.455	0.135 to 1.537	0.205	0.441	Not applicable (detection model)
Genus	*Lachnospira*	SPMS vs HC	LFC	-0.485	-1.336 to 0.366	0.267	0.441	Passed
Genus	*Dorea*	SPMS vs HC	LFC	-0.048	-0.737 to 0.642	0.893	0.893	Passed
Genus	*Faecalibacterium*	SPMS vs HC	LFC	-0.826	-1.533 to -0.119	0.025	0.418	Passed
Genus	*Prevotella*	SPMS vs HC	LFC	-1.698	-3.723 to 0.326	0.104	0.441	Passed
Genus	*Akkermansia*	SPMS vs HC	LFC	-0.468	-1.464 to 0.528	0.363	0.441	Passed
Genus	*Blautia*	SPMS vs HC	LFC	-0.306	-0.942 to 0.331	0.349	0.441	Passed
Genus	*Ruminococcus*	SPMS vs HC	LFC	-0.458	-1.329 to 0.413	0.306	0.441	Passed
Genus	*Agathobaculum*	SPMS vs HC	LFC	-0.532	-1.470 to 0.406	0.270	0.441	Passed
Genus	*Alistipes*	SPMS vs HC	LFC	0.422	-0.368 to 1.213	0.298	0.441	Passed
Phylum	Bacillota	SPMS vs RRMS	LFC	-0.079	-0.582 to 0.424	0.759	0.807	Passed
Phylum	Bacteroidota	SPMS vs RRMS	LFC	0.109	-0.441 to 0.660	0.698	0.791	Passed
Family	Lachnospiraceae	SPMS vs RRMS	LFC	-0.148	-0.780 to 0.484	0.648	0.791	Passed
Family	Enterobacteriaceae	SPMS vs RRMS	LFC	0.568	-0.599 to 1.736	0.345	0.791	Passed
Genus	*Bifidobacterium*	SPMS vs RRMS	LFC	-0.372	-1.416 to 0.673	0.489	0.791	Passed

Taxonomic level	Taxon	Contrast	Effect type	Estimate	95% CI	p	q	Pseudo-count sensitivity
Genus	*Roseburia*	SPMS vs RRMS	LFC	-0.231	-1.107 to 0.645	0.607	0.791	Passed
Genus	*Coprococcus*	SPMS vs RRMS	LFC	-0.738	-1.686 to 0.209	0.133	0.791	Passed
Genus	*Butyricicoccus*	SPMS vs RRMS	OR for detection	2.149	0.501 to 9.223	0.303	0.791	Not applicable (detection model)
Genus	*Lachnospira*	SPMS vs RRMS	LFC	-0.293	-1.168 to 0.582	0.515	0.791	Passed
Genus	*Dorea*	SPMS vs RRMS	LFC	0.145	-0.551 to 0.842	0.684	0.791	Passed
Genus	*Faecalibacterium*	SPMS vs RRMS	LFC	-0.540	-1.317 to 0.237	0.179	0.791	Passed
Genus	*Prevotella*	SPMS vs RRMS	LFC	-1.375	-3.678 to 0.927	0.247	0.791	Passed
Genus	*Akkermansia*	SPMS vs RRMS	LFC	0.398	-0.833 to 1.628	0.532	0.791	Passed
Genus	*Blautia*	SPMS vs RRMS	LFC	-0.002	-0.716 to 0.712	0.996	0.996	Passed
Genus	*Ruminococcus*	SPMS vs RRMS	LFC	-0.207	-1.145 to 0.731	0.667	0.791	Passed
Genus	*Agathobaculum*	SPMS vs RRMS	LFC	-0.241	-0.973 to 0.491	0.521	0.791	Passed
Genus	*Alistipes*	SPMS vs RRMS	LFC	0.396	-0.379 to 1.172	0.321	0.791	Passed

**Abundance-based taxa were analysed using age- and sex-adjusted ANCOM-BC2 models. Butyricicoccus was analysed as a detection outcome using logistic regression because of low prevalence. HC was the reference group for the RRMS vs HC and SPMS vs HC contrasts; RRMS was the reference group for the SPMS vs RRMS contrast. q-values were obtained by Benjamini-Hochberg correction separately across the 17 predefined taxonomic comparisons within each contrast. Pseudo-count sensitivity applies to ANCOM-BC2 abundance models. LFC, log fold change; OR, odds ratio.*

Figure S3a. Exploratory species-level LEfSe (Linear Discriminant Analysis Effect Size) analysis comparing RRMS and HC

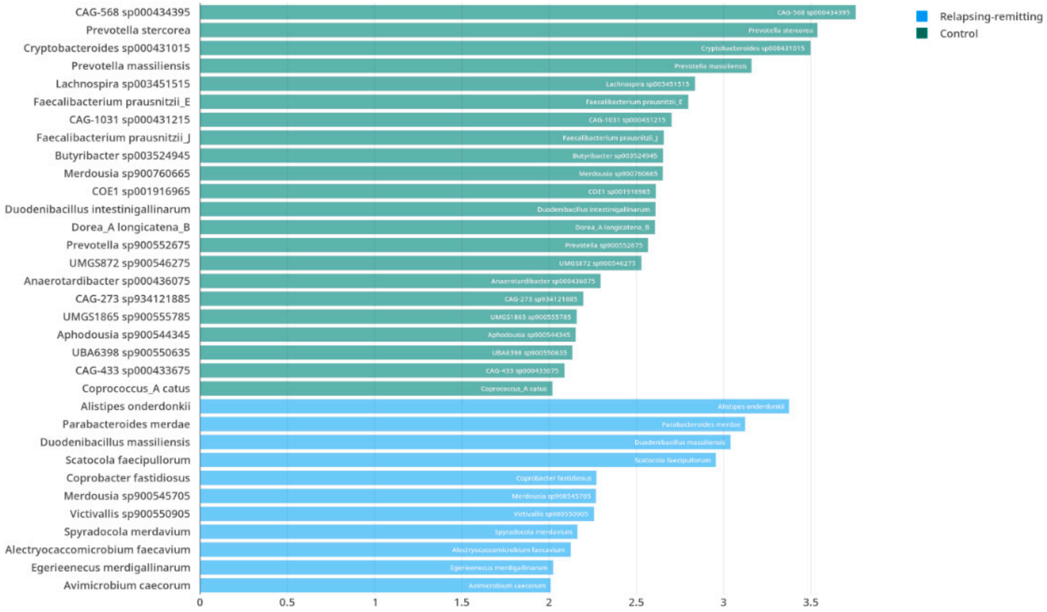

**Species meeting the prespecified exploratory criteria of nominal p<0.05 and absolute LDA score ≥2.0 are shown. Blue bars indicate features enriched in RRMS and green bars features enriched in HC.*

Figure S3b. Exploratory species-level LEfSe analysis comparing SPMS and HC

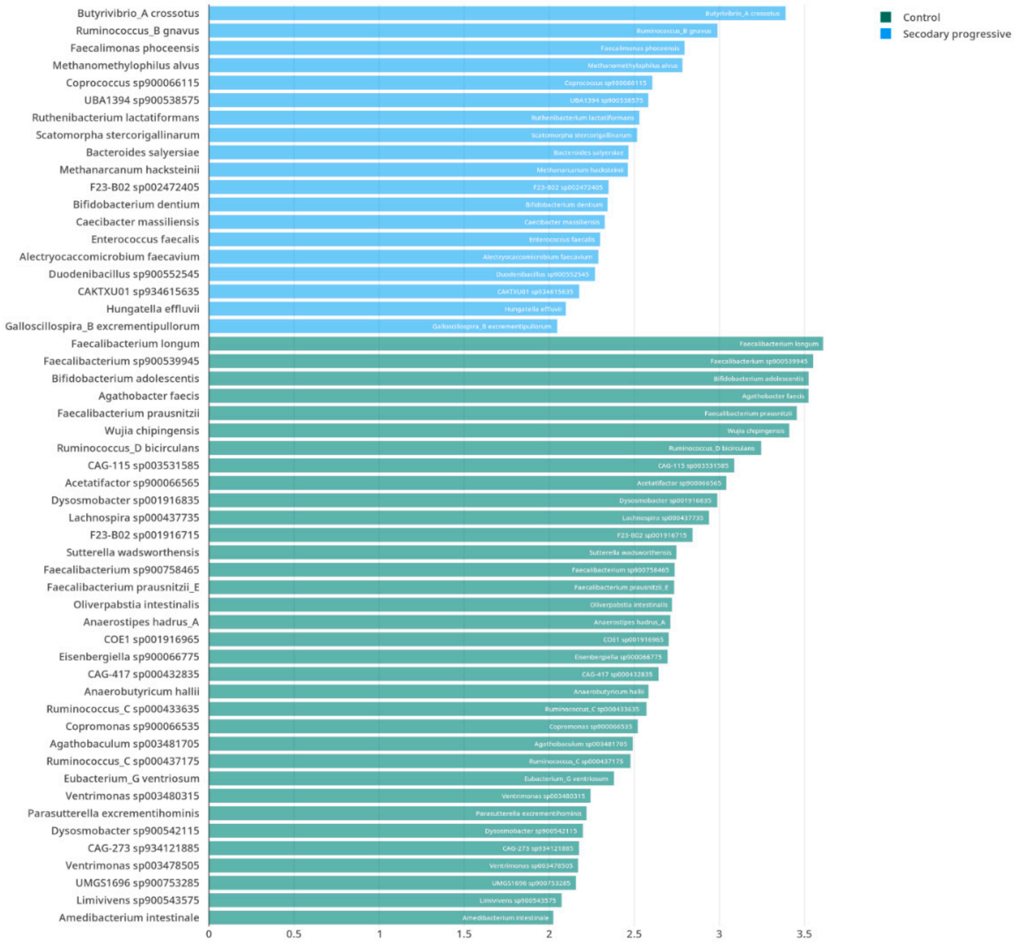

**Species meeting the prespecified exploratory criteria of nominal p<0.05 and absolute LDA score ≥2.0 are shown. Blue bars indicate features enriched in SPMS and green bars features enriched in HC.*

Table S6a. Associations of predefined taxonomic features with neurological disability

Taxon	Disability measure	N	Effect type	Estimate	95% CI lower	95% CI upper	p	q	Pseudo-count sensitivity
Bifidobacterium	EDSS	59	LFC	-0.066	-0.729	0.596	0.845	0.957	Passed
Ruminococcus	EDSS	59	LFC	0.304	-0.351	0.959	0.367	0.735	Passed
Blautia	EDSS	59	LFC	-0.362	-0.652	-0.072	0.018	0.200	Passed
Alistipes	EDSS	59	LFC	0.029	-0.471	0.529	0.910	0.959	Passed
Lachnospira	EDSS	59	LFC	-0.561	-1.161	0.038	0.072	0.313	Passed
**Roseburia**	EDSS	59	LFC	-0.900	-1.403	-0.397	**<0.001**	**0.064**	**Failed**
Faecalibacterium	EDSS	59	LFC	-0.386	-0.838	0.067	0.100	0.355	Passed
Agathobaculum	EDSS	59	LFC	-0.599	-0.999	-0.199	0.005	0.097	Failed
Akkermansia	EDSS	59	LFC	0.116	-0.759	0.992	0.796	0.941	Passed
Coprococcus	EDSS	59	LFC	-0.192	-0.729	0.345	0.486	0.768	Passed
Dorea	EDSS	59	LFC	-0.273	-0.556	0.009	0.064	0.313	Passed
Prevotella	EDSS	59	LFC	-0.526	-1.885	0.834	0.452	0.754	Passed
Lachnospiraceae	EDSS	59	LFC	-0.330	-0.680	0.019	0.069	0.313	Passed
Enterobacteriaceae	EDSS	59	LFC	0.146	-0.951	1.243	0.795	0.941	Passed
Bacillota	EDSS	59	LFC	-0.241	-0.575	0.093	0.164	0.428	Passed
Bacteroidota	EDSS	59	LFC	-0.357	-0.670	-0.044	0.029	0.250	Passed
Bifidobacterium	T25FW	59	LFC	-0.288	-0.871	0.294	0.336	0.715	Passed
Ruminococcus	T25FW	59	LFC	-0.031	-0.478	0.415	0.892	0.959	Passed
Blautia	T25FW	59	LFC	-0.085	-0.403	0.233	0.603	0.854	Passed
Alistipes	T25FW	59	LFC	-0.050	-0.449	0.350	0.809	0.941	Passed
Lachnospira	T25FW	59	LFC	-0.574	-1.046	-0.101	0.021	0.204	Passed
Roseburia	T25FW	59	LFC	-0.302	-0.720	0.116	0.163	0.428	Passed
Faecalibacterium	T25FW	59	LFC	-0.135	-0.528	0.257	0.501	0.768	Passed
Agathobaculum	T25FW	59	LFC	-0.311	-0.697	0.075	0.120	0.355	Passed
Akkermansia	T25FW	59	LFC	-0.407	-0.957	0.144	0.159	0.428	Passed
Coprococcus	T25FW	59	LFC	-0.146	-0.577	0.284	0.508	0.768	Passed
Dorea	T25FW	59	LFC	-0.127	-0.414	0.160	0.389	0.735	Passed
Prevotella	T25FW	59	LFC	0.074	-1.209	1.357	0.910	0.959	Passed
Lachnospiraceae	T25FW	59	LFC	-0.038	-0.354	0.279	0.817	0.941	Passed
Enterobacteriaceae	T25FW	59	LFC	0.175	-0.630	0.980	0.672	0.933	Passed
Bacillota	T25FW	59	LFC	-0.102	-0.431	0.226	0.544	0.805	Passed

Bacteroidota	T25FW	59	LFC	-0.046	-0.327	0.236	0.751	0.941	Passed
Bifidobacterium	9HPT, dominant hand	59	LFC	-0.015	-0.444	0.414	0.946	0.966	Passed
Ruminococcus	9HPT, dominant hand	59	LFC	0.449	-0.006	0.905	0.058	0.313	Passed
Blautia	9HPT, dominant hand	59	LFC	-0.089	-0.272	0.095	0.347	0.715	Passed
Alistipes	9HPT, dominant hand	59	LFC	0.131	-0.194	0.456	0.432	0.754	Passed
Lachnospira	9HPT, dominant hand	59	LFC	-0.139	-0.497	0.219	0.449	0.754	Passed
Roseburia	9HPT, dominant hand	59	LFC	-0.468	-0.836	-0.099	0.016	0.200	Failed
Faecalibacterium	9HPT, dominant hand	59	LFC	-0.249	-0.489	-0.009	0.047	0.313	Passed
Agathobaculum	9HPT, dominant hand	59	LFC	-0.265	-0.590	0.060	0.116	0.355	Passed
Akkermansia	9HPT, dominant hand	59	LFC	0.077	-0.428	0.582	0.768	0.941	Passed
Coprococcus	9HPT, dominant hand	59	LFC	0.022	-0.347	0.391	0.908	0.959	Passed
Dorea	9HPT, dominant hand	59	LFC	-0.042	-0.260	0.176	0.709	0.941	Passed
Prevotella	9HPT, dominant hand	59	LFC	-0.029	-1.167	1.109	0.961	0.966	Passed
Lachnospiraceae	9HPT, dominant hand	59	LFC	-0.194	-0.382	-0.006	0.048	0.313	Passed
Enterobacteriaceae	9HPT, dominant hand	59	LFC	0.439	-0.097	0.974	0.115	0.355	Passed
Bacillota	9HPT, dominant hand	59	LFC	-0.036	-0.264	0.192	0.757	0.941	Passed
Bacteroidota	9HPT, dominant hand	59	LFC	-0.197	-0.403	0.010	0.067	0.313	Passed
Bifidobacterium	9HPT, non-dominant hand	58	LFC	-0.295	-0.740	0.151	0.200	0.487	Passed
Ruminococcus	9HPT, non-dominant hand	58	LFC	0.198	-0.244	0.641	0.383	0.735	Passed
Blautia	9HPT, non-dominant hand	58	LFC	-0.122	-0.332	0.087	0.259	0.606	Passed
Alistipes	9HPT, non-dominant hand	58	LFC	0.197	-0.165	0.558	0.291	0.659	Passed
Lachnospira	9HPT, non-dominant hand	58	LFC	-0.130	-0.455	0.195	0.436	0.754	Passed
Roseburia	9HPT, non-dominant hand	58	LFC	-0.611	-1.005	-0.217	0.004	0.097	Failed
Faecalibacterium	9HPT, non-dominant hand	58	LFC	-0.281	-0.584	0.022	0.075	0.313	Passed
Agathobaculum	9HPT, non-dominant hand	58	LFC	-0.119	-0.457	0.219	0.493	0.768	Passed
Akkermansia	9HPT, non-dominant hand	58	LFC	0.463	-0.097	1.023	0.117	0.355	Passed
Coprococcus	9HPT, non-dominant hand	58	LFC	-0.129	-0.569	0.312	0.569	0.824	Passed
Dorea	9HPT, non-dominant hand	58	LFC	-0.141	-0.407	0.125	0.304	0.667	Passed
Prevotella	9HPT, non-dominant hand	58	LFC	-0.487	-1.755	0.781	0.455	0.754	Passed
Lachnospiraceae	9HPT, non-dominant hand	58	LFC	-0.146	-0.357	0.065	0.180	0.454	Passed
Enterobacteriaceae	9HPT, non-dominant hand	58	LFC	0.125	-0.487	0.738	0.690	0.939	Passed
Bacillota	9HPT, non-dominant hand	58	LFC	0.010	-0.183	0.204	0.917	0.959	Passed
Bacteroidota	9HPT, non-dominant hand	58	LFC	-0.229	-0.480	0.021	0.078	0.313	Passed
Butyricicoccus	EDSS	59	OR for detection	0.329	0.091	1.183	0.089	0.335	Not applicable (detection model)
Butyricicoccus	T25FW	59	OR for detection	0.149	0.039	0.575	0.006	0.097	Not applicable (detection model)

Butyricicoccus	9HPT, dominant hand	59	OR for detection	1.017	0.474	2.179	0.966	0.966	Not applicable (detection model)
Butyricicoccus	9HPT, non-dominant hand	58	OR for detection	1.152	0.515	2.575	0.731	0.941	Not applicable (detection model)

**Abundance-based taxonomic associations were estimated using ANCOM-BC2 among participants with MS, adjusted for phenotype, age, and sex. Butyricicoccus was analysed as a detection outcome by logistic regression. q-values were obtained by Benjamini-Hochberg correction across the 68 predefined taxon-disability associations. Pseudo-count sensitivity applies to ANCOM-BC2 abundance models. LFC, log fold change.*

Table S6b. Taxon-by-phenotype interactions for neurological disability

Taxon	Disability measure	N	Effect type	Estimate	95% CI lower	95% CI upper	p interaction	q interaction	Pseudo-count sensitivity
Bifidobacterium	EDSS	59	Interaction LFC	1.272	-0.053	2.597	0.066	0.833	Not performed (not FDR-significant)
Ruminococcus	EDSS	59	Interaction LFC	-1.259	-2.759	0.241	0.106	0.833	Not performed (not FDR-significant)
Blautia	EDSS	59	Interaction LFC	0.004	-0.571	0.579	0.989	0.994	Not performed (not FDR-significant)
Alistipes	EDSS	59	Interaction LFC	-0.604	-1.650	0.443	0.264	0.906	Not performed (not FDR-significant)
Lachnospira	EDSS	59	Interaction LFC	-0.513	-1.686	0.661	0.396	0.906	Not performed (not FDR-significant)
Roseburia	EDSS	59	Interaction LFC	-0.117	-1.205	0.971	0.833	0.972	Not performed (not FDR-significant)
Faecalibacterium	EDSS	59	Interaction LFC	-0.148	-1.070	0.775	0.755	0.964	Not performed (not FDR-significant)
Agathobaculum	EDSS	59	Interaction LFC	-0.285	-1.152	0.582	0.522	0.906	Not performed (not FDR-significant)
**Akkermansia**	EDSS	59	Interaction LFC	0.716	-1.309	2.741	**0.494**	**0.906**	Not performed (not FDR-significant)
Coprococcus	EDSS	59	Interaction LFC	-0.361	-1.573	0.851	0.562	0.906	Not performed (not FDR-significant)
Dorea	EDSS	59	Interaction LFC	-0.388	-0.962	0.186	0.191	0.906	Not performed (not FDR-significant)
Prevotella	EDSS	59	Interaction LFC	1.620	-1.244	4.485	0.273	0.906	Not performed (not FDR-significant)
Lachnospiraceae	EDSS	59	Interaction LFC	-0.502	-1.200	0.195	0.164	0.856	Not performed (not FDR-significant)
Enterobacteriaceae	EDSS	59	Interaction LFC	1.341	-0.822	3.504	0.231	0.906	Not performed (not FDR-significant)
Bacillota	EDSS	59	Interaction LFC	-0.559	-1.174	0.055	0.080	0.833	Not performed (not FDR-significant)
Bacteroidota	EDSS	59	Interaction LFC	0.111	-0.610	0.832	0.763	0.964	Not performed (not FDR-significant)
Bifidobacterium	T25FW	59	Interaction LFC	1.146	-0.337	2.629	0.137	0.844	Not performed (not FDR-significant)
Ruminococcus	T25FW	59	Interaction LFC	-0.530	-1.797	0.737	0.416	0.906	Not performed (not FDR-significant)
Blautia	T25FW	59	Interaction LFC	0.282	-0.449	1.013	0.453	0.906	Not performed (not FDR-significant)
Alistipes	T25FW	59	Interaction LFC	-0.130	-1.187	0.928	0.811	0.972	Not performed (not FDR-significant)
Lachnospira	T25FW	59	Interaction LFC	0.004	-0.958	0.966	0.994	0.994	Not performed (not FDR-significant)
Roseburia	T25FW	59	Interaction LFC	0.248	-0.871	1.366	0.666	0.906	Not performed (not FDR-significant)
Faecalibacterium	T25FW	59	Interaction LFC	0.089	-0.810	0.989	0.846	0.972	Not performed (not FDR-significant)
Agathobaculum	T25FW	59	Interaction LFC	-0.097	-1.174	0.981	0.861	0.972	Not performed (not FDR-significant)
Akkermansia	T25FW	59	Interaction LFC	-0.751	-2.081	0.580	0.279	0.906	Not performed (not FDR-significant)
Coprococcus	T25FW	59	Interaction LFC	-0.351	-1.540	0.837	0.565	0.906	Not performed (not FDR-significant)

Dorea	T25FW	59	Interaction LFC	0.179	-0.558	0.916	0.635	0.906	Not performed (not FDR-significant)
Prevotella	T25FW	59	Interaction LFC	-1.167	-4.306	1.972	0.470	0.906	Not performed (not FDR-significant)
Lachnospiraceae	T25FW	59	Interaction LFC	-0.038	-0.742	0.666	0.916	0.974	Not performed (not FDR-significant)
Enterobacteriaceae	T25FW	59	Interaction LFC	0.543	-1.302	2.387	0.567	0.906	Not performed (not FDR-significant)
Bacillota	T25FW	59	Interaction LFC	-0.158	-0.845	0.529	0.654	0.906	Not performed (not FDR-significant)
Bacteroidota	T25FW	59	Interaction LFC	0.236	-0.466	0.938	0.513	0.906	Not performed (not FDR-significant)
Bifidobacterium	9HPT, dominant hand	59	Interaction LFC	0.425	-0.352	1.202	0.289	0.906	Not performed (not FDR-significant)
Ruminococcus	9HPT, dominant hand	59	Interaction LFC	0.129	-0.715	0.973	0.766	0.964	Not performed (not FDR-significant)
Blautia	9HPT, dominant hand	59	Interaction LFC	0.013	-0.450	0.476	0.957	0.986	Not performed (not FDR-significant)
Alistipes	9HPT, dominant hand	59	Interaction LFC	0.129	-0.396	0.654	0.632	0.906	Not performed (not FDR-significant)
Lachnospira	9HPT, dominant hand	59	Interaction LFC	0.220	-0.341	0.782	0.445	0.906	Not performed (not FDR-significant)
Roseburia	9HPT, dominant hand	59	Interaction LFC	-0.366	-1.143	0.412	0.361	0.906	Not performed (not FDR-significant)
Faecalibacterium	9HPT, dominant hand	59	Interaction LFC	-0.115	-0.656	0.427	0.680	0.906	Not performed (not FDR-significant)
Agathobaculum	9HPT, dominant hand	59	Interaction LFC	-0.172	-0.850	0.505	0.620	0.906	Not performed (not FDR-significant)
Akkermansia	9HPT, dominant hand	59	Interaction LFC	2.762	1.874	3.650	<0.001	<0.001	Failed
Coprococcus	9HPT, dominant hand	59	Interaction LFC	0.062	-0.892	1.016	0.899	0.972	Not performed (not FDR-significant)
Dorea	9HPT, dominant hand	59	Interaction LFC	-0.137	-0.467	0.193	0.418	0.906	Not performed (not FDR-significant)
Prevotella	9HPT, dominant hand	59	Interaction LFC	-1.836	-4.125	0.454	0.122	0.833	Not performed (not FDR-significant)
Lachnospiraceae	9HPT, dominant hand	59	Interaction LFC	-0.292	-0.629	0.045	0.095	0.833	Not performed (not FDR-significant)
Enterobacteriaceae	9HPT, dominant hand	59	Interaction LFC	0.363	-0.827	1.553	0.553	0.906	Not performed (not FDR-significant)
Bacillota	9HPT, dominant hand	59	Interaction LFC	-0.112	-0.523	0.299	0.595	0.906	Not performed (not FDR-significant)
Bacteroidota	9HPT, dominant hand	59	Interaction LFC	0.031	-0.446	0.508	0.900	0.972	Not performed (not FDR-significant)

Bifidobacterium	9HPT, non-dominant hand	58	Interaction LFC	0.765	-0.051	1.581	0.072	0.833	Not performed (not FDR-significant)
Ruminococcus	9HPT, non-dominant hand	58	Interaction LFC	-0.047	-0.787	0.692	0.900	0.972	Not performed (not FDR-significant)
Blautia	9HPT, non-dominant hand	58	Interaction LFC	-0.138	-0.671	0.396	0.615	0.906	Not performed (not FDR-significant)
Alistipes	9HPT, non-dominant hand	58	Interaction LFC	0.638	0.031	1.244	0.044	0.833	Not performed (not FDR-significant)
Lachnospira	9HPT, non-dominant hand	58	Interaction LFC	-0.053	-0.548	0.441	0.833	0.972	Not performed (not FDR-significant)
Roseburia	9HPT, non-dominant hand	58	Interaction LFC	-0.560	-1.258	0.137	0.122	0.833	Not performed (not FDR-significant)
Faecalibacterium	9HPT, non-dominant hand	58	Interaction LFC	-0.025	-0.598	0.548	0.932	0.975	Not performed (not FDR-significant)
Agathobaculum	9HPT, non-dominant hand	58	Interaction LFC	0.147	-0.454	0.749	0.633	0.906	Not performed (not FDR-significant)
Akkermansia	9HPT, non-dominant hand	58	Interaction LFC	2.227	1.272	3.182	<0.001	0.004	Failed
Coprococcus	9HPT, non-dominant hand	58	Interaction LFC	0.345	-0.446	1.136	0.397	0.906	Not performed (not FDR-significant)
Dorea	9HPT, non-dominant hand	58	Interaction LFC	-0.221	-0.590	0.147	0.245	0.906	Not performed (not FDR-significant)
Prevotella	9HPT, non-dominant hand	58	Interaction LFC	-0.709	-2.870	1.452	0.523	0.906	Not performed (not FDR-significant)
Lachnospiraceae	9HPT, non-dominant hand	58	Interaction LFC	-0.247	-0.580	0.085	0.151	0.855	Not performed (not FDR-significant)
Enterobacteriaceae	9HPT, non-dominant hand	58	Interaction LFC	0.145	-0.875	1.166	0.781	0.966	Not performed (not FDR-significant)
Bacillota	9HPT, non-dominant hand	58	Interaction LFC	0.079	-0.237	0.395	0.626	0.906	Not performed (not FDR-significant)
Bacteroidota	9HPT, non-dominant hand	58	Interaction LFC	0.131	-0.338	0.600	0.586	0.906	Not performed (not FDR-significant)
Butyricicoccus	EDSS	59	Interaction OR	6.553	0.180	238.443	0.305	0.906	Not applicable (detection model)
Butyricicoccus	T25FW	59	Interaction OR	0.388	0.020	7.401	0.529	0.906	Not applicable (detection model)
Butyricicoccus	9HPT, dominant hand	59	Interaction OR	2.162	0.262	17.801	0.474	0.906	Not applicable (detection model)
Butyricicoccus	9HPT, non-dominant hand	58	Interaction OR	0.675	0.120	3.800	0.655	0.906	Not applicable (detection model)

Taxon-by-phenotype interactions were evaluated with adjustment for age and sex. FDR-significant ANCOM-BC2 interactions were considered robust only if they also passed pseudo-count sensitivity analysis. Butyricicoccus detection used logistic regression. q-values were obtained across the 68 predefined interaction tests. LFC, log fold change.

Table S7a. Associations of predefined taxonomic features with circulating biomarkers

Taxon	Biomarker	N	Effect type	Estimate	95% CI lower	95% CI upper	p	q	Pseudo-count sensitivity
Bifidobacterium	GFAP	59	LFC	-0.251	-0.706	0.205	0.286	0.957	Not performed (no FDR-significant association)
Ruminococcus	GFAP	59	LFC	-0.224	-0.623	0.174	0.275	0.957	Not performed (no FDR-significant association)
Blautia	GFAP	59	LFC	-0.113	-0.344	0.119	0.343	0.957	Not performed (no FDR-significant association)
Alistipes	GFAP	59	LFC	0.057	-0.194	0.309	0.657	0.957	Not performed (no FDR-significant association)
Lachnospira	GFAP	59	LFC	-0.016	-0.272	0.240	0.903	0.957	Not performed (no FDR-significant association)
Roseburia	GFAP	59	LFC	0.067	-0.281	0.414	0.709	0.957	Not performed (no FDR-significant association)
Faecalibacterium	GFAP	59	LFC	0.190	-0.063	0.443	0.147	0.680	Not performed (no FDR-significant association)
Agathobaculum	GFAP	59	LFC	0.065	-0.179	0.309	0.605	0.957	Not performed (no FDR-significant association)
Akkermansia	GFAP	59	LFC	-0.363	-0.832	0.105	0.140	0.680	Not performed (no FDR-significant association)
Coprococcus	GFAP	59	LFC	0.253	-0.080	0.586	0.143	0.680	Not performed (no FDR-significant association)
Dorea	GFAP	59	LFC	-0.053	-0.225	0.118	0.545	0.957	Not performed (no FDR-significant association)
Prevotella	GFAP	59	LFC	0.342	-0.645	1.330	0.500	0.957	Not performed (no FDR-significant association)
Lachnospiraceae	GFAP	59	LFC	0.049	-0.128	0.225	0.592	0.957	Not performed (no FDR-significant association)
Enterobacteriaceae	GFAP	59	LFC	0.254	-0.341	0.849	0.407	0.957	Not performed (no FDR-significant association)
Bacillota	GFAP	59	LFC	0.051	-0.153	0.255	0.628	0.957	Not performed (no FDR-significant association)
Bacteroidota	GFAP	59	LFC	0.108	-0.083	0.299	0.274	0.957	Not performed (no FDR-significant association)
Bifidobacterium	NfL	59	LFC	0.244	-0.061	0.548	0.123	0.680	Not performed (no FDR-significant association)
Ruminococcus	NfL	59	LFC	-0.417	-0.898	0.064	0.095	0.680	Not performed (no FDR-significant association)
Blautia	NfL	59	LFC	-0.115	-0.427	0.197	0.474	0.957	Not performed (no FDR-significant association)
Alistipes	NfL	59	LFC	-0.019	-0.226	0.187	0.855	0.957	Not performed (no FDR-significant association)
Lachnospira	NfL	59	LFC	-0.049	-0.365	0.267	0.763	0.957	Not performed (no FDR-significant association)
Roseburia	NfL	59	LFC	0.088	-0.259	0.435	0.620	0.957	Not performed (no FDR-significant association)
Faecalibacterium	NfL	59	LFC	0.218	0.064	0.372	0.007	0.370	Not performed (no FDR-significant association)
Agathobaculum	NfL	59	LFC	-0.013	-0.280	0.253	0.924	0.957	Not performed (no FDR-significant association)
Akkermansia	NfL	59	LFC	-0.024	-0.549	0.500	0.928	0.957	Not performed (no FDR-significant association)
Coprococcus	NfL	59	LFC	0.060	-0.172	0.292	0.615	0.957	Not performed (no FDR-significant association)
Dorea	NfL	59	LFC	-0.035	-0.211	0.142	0.701	0.957	Not performed (no FDR-significant association)
Prevotella	NfL	59	LFC	0.719	0.099	1.338	0.027	0.465	Not performed (no FDR-significant association)
Lachnospiraceae	NfL	59	LFC	0.020	-0.182	0.222	0.844	0.957	Not performed (no FDR-significant association)
Enterobacteriaceae	NfL	59	LFC	0.773	0.176	1.370	0.015	0.370	Not performed (no FDR-significant association)
Bacillota	NfL	59	LFC	0.107	-0.026	0.239	0.121	0.680	Not performed (no FDR-significant association)

Bacteroidota	NfL	59	LFC	0.083	-0.088	0.253	0.346	0.957	Not performed (no FDR-significant association)
Bifidobacterium	S100B detection	59	LFC	0.048	-0.886	0.982	0.920	0.957	Not performed (no FDR-significant association)
Ruminococcus	S100B detection	59	LFC	0.345	-0.375	1.065	0.352	0.957	Not performed (no FDR-significant association)
Blautia	S100B detection	59	LFC	-0.016	-0.412	0.381	0.938	0.957	Not performed (no FDR-significant association)
Alistipes	S100B detection	59	LFC	0.199	-0.320	0.717	0.456	0.957	Not performed (no FDR-significant association)
Lachnospira	S100B detection	59	LFC	0.219	-0.324	0.762	0.433	0.957	Not performed (no FDR-significant association)
Roseburia	S100B detection	59	LFC	0.231	-0.454	0.916	0.512	0.957	Not performed (no FDR-significant association)
Faecalibacterium	S100B detection	59	LFC	0.074	-0.411	0.558	0.767	0.957	Not performed (no FDR-significant association)
Agathobaculum	S100B detection	59	LFC	0.241	-0.331	0.814	0.412	0.957	Not performed (no FDR-significant association)
Akkermansia	S100B detection	59	LFC	0.789	0.014	1.565	0.056	0.680	Not performed (no FDR-significant association)
Coprococcus	S100B detection	59	LFC	-0.079	-0.765	0.608	0.823	0.957	Not performed (no FDR-significant association)
Dorea	S100B detection	59	LFC	-0.058	-0.393	0.278	0.737	0.957	Not performed (no FDR-significant association)
Prevotella	S100B detection	59	LFC	0.349	-1.386	2.084	0.695	0.957	Not performed (no FDR-significant association)
Lachnospiraceae	S100B detection	59	LFC	0.118	-0.228	0.463	0.507	0.957	Not performed (no FDR-significant association)
Enterobacteriaceae	S100B detection	59	LFC	0.517	-0.525	1.559	0.336	0.957	Not performed (no FDR-significant association)
Bacillota	S100B detection	59	LFC	-0.051	-0.466	0.365	0.812	0.957	Not performed (no FDR-significant association)
Bacteroidota	S100B detection	59	LFC	0.032	-0.369	0.433	0.877	0.957	Not performed (no FDR-significant association)
Butyricicoccus	GFAP	59	OR for detection	0.527	0.243	1.140	0.104	0.680	Not applicable (detection model)
Butyricicoccus	NfL	59	OR for detection	1.179	0.617	2.254	0.618	0.957	Not applicable (detection model)
Butyricicoccus	S100B detection	59	OR for detection	1.008	0.269	3.777	0.990	0.990	Not applicable (detection model)

**Taxonomic associations with biomarkers were evaluated among participants with MS with adjustment for phenotype, age, and sex. Butyricicoccus was analysed as a detection outcome by logistic regression. q-values were obtained by Benjamini-Hochberg correction across the 51 predefined taxon-biomarker associations. LFC, log fold change.*

Table S7b. Taxon-by-phenotype interactions for circulating biomarkers

Taxon	Biomarker	N	Effect type	Estimate	95% CI lower	95% CI upper	p interaction	q interaction	Pseudo-count sensitivity
Bifidobacterium	GFAP	59	Interaction LFC	-1.054	-1.955	-0.153	0.026	0.192	Not performed (no FDR-significant interaction)
Ruminococcus	GFAP	59	Interaction LFC	0.106	-0.663	0.875	0.789	0.913	Not performed (no FDR-significant interaction)
Blautia	GFAP	59	Interaction LFC	0.185	-0.256	0.625	0.415	0.826	Not performed (no FDR-significant interaction)
Alistipes	GFAP	59	Interaction LFC	0.550	0.096	1.003	0.021	0.192	Not performed (no FDR-significant interaction)
Lachnospira	GFAP	59	Interaction LFC	0.064	-0.465	0.593	0.814	0.913	Not performed (no FDR-significant interaction)
Roseburia	GFAP	59	Interaction LFC	0.080	-0.618	0.778	0.824	0.913	Not performed (no FDR-significant interaction)
Faecalibacterium	GFAP	59	Interaction LFC	0.268	-0.232	0.769	0.298	0.801	Not performed (no FDR-significant interaction)
Agathobaculum	GFAP	59	Interaction LFC	0.331	-0.196	0.857	0.224	0.713	Not performed (no FDR-significant interaction)
Akkermansia	GFAP	59	Interaction LFC	-0.299	-1.254	0.655	0.544	0.852	Not performed (no FDR-significant interaction)
Coprococcus	GFAP	59	Interaction LFC	-0.989	-1.598	-0.380	0.003	0.130	Not performed (no FDR-significant interaction)
Dorea	GFAP	59	Interaction LFC	-0.264	-0.598	0.071	0.128	0.595	Not performed (no FDR-significant interaction)
Prevotella	GFAP	59	Interaction LFC	-0.831	-2.879	1.217	0.430	0.826	Not performed (no FDR-significant interaction)
Lachnospiraceae	GFAP	59	Interaction LFC	0.066	-0.273	0.406	0.704	0.897	Not performed (no FDR-significant interaction)
Enterobacteriaceae	GFAP	59	Interaction LFC	0.149	-1.029	1.326	0.806	0.913	Not performed (no FDR-significant interaction)
Bacillota	GFAP	59	Interaction LFC	-0.156	-0.548	0.235	0.438	0.826	Not performed (no FDR-significant interaction)
Bacteroidota	GFAP	59	Interaction LFC	0.047	-0.337	0.431	0.810	0.913	Not performed (no FDR-significant interaction)
Bifidobacterium	NfL	59	Interaction LFC	0.725	0.126	1.324	0.022	0.192	Not performed (no FDR-significant interaction)
Ruminococcus	NfL	59	Interaction LFC	0.926	0.197	1.655	0.016	0.192	Not performed (no FDR-significant interaction)
Blautia	NfL	59	Interaction LFC	0.554	0.092	1.016	0.023	0.192	Not performed (no FDR-significant interaction)
Alistipes	NfL	59	Interaction LFC	0.295	-0.124	0.714	0.173	0.647	Not performed (no FDR-significant interaction)
Lachnospira	NfL	59	Interaction LFC	-0.296	-0.834	0.242	0.285	0.801	Not performed (no FDR-significant interaction)
Roseburia	NfL	59	Interaction LFC	-0.511	-0.992	-0.031	0.042	0.268	Not performed (no FDR-significant interaction)
Faecalibacterium	NfL	59	Interaction LFC	-0.096	-0.487	0.295	0.633	0.870	Not performed (no FDR-significant interaction)
Agathobaculum	NfL	59	Interaction LFC	-0.085	-0.465	0.296	0.665	0.870	Not performed (no FDR-significant interaction)
Akkermansia	NfL	59	Interaction LFC	1.186	0.343	2.028	0.011	0.192	Not performed (no FDR-significant interaction)
Coprococcus	NfL	59	Interaction LFC	0.322	-0.155	0.799	0.192	0.652	Not performed (no FDR-significant interaction)
Dorea	NfL	59	Interaction LFC	0.280	-0.027	0.587	0.080	0.443	Not performed (no FDR-significant interaction)
Prevotella	NfL	59	Interaction LFC	-0.075	-1.363	1.213	0.910	0.934	Not performed (no FDR-significant interaction)
Lachnospiraceae	NfL	59	Interaction LFC	0.132	-0.167	0.432	0.390	0.826	Not performed (no FDR-significant interaction)

Enterobacteriaceae	NfL	59	Interaction LFC	-0.699	-1.627	0.230	0.147	0.625	Not performed (no FDR-significant interaction)
Bacillota	NfL	59	Interaction LFC	0.186	-0.081	0.454	0.178	0.647	Not performed (no FDR-significant interaction)
Bacteroidota	NfL	59	Interaction LFC	-0.127	-0.436	0.183	0.425	0.826	Not performed (no FDR-significant interaction)
Bifidobacterium	S100B detection	59	Interaction LFC	-0.482	-2.622	1.657	0.661	0.870	Not performed (no FDR-significant interaction)
Ruminococcus	S100B detection	59	Interaction LFC	-0.722	-2.294	0.850	0.372	0.826	Not performed (no FDR-significant interaction)
Blautia	S100B detection	59	Interaction LFC	0.077	-0.904	1.058	0.878	0.934	Not performed (no FDR-significant interaction)
Alistipes	S100B detection	59	Interaction LFC	-0.724	-1.931	0.483	0.245	0.736	Not performed (no FDR-significant interaction)
Lachnospira	S100B detection	59	Interaction LFC	0.397	-0.895	1.689	0.549	0.852	Not performed (no FDR-significant interaction)
Roseburia	S100B detection	59	Interaction LFC	0.424	-1.089	1.937	0.585	0.852	Not performed (no FDR-significant interaction)
Faecalibacterium	S100B detection	59	Interaction LFC	0.355	-0.743	1.454	0.529	0.852	Not performed (no FDR-significant interaction)
Agathobaculum	S100B detection	59	Interaction LFC	0.573	-0.862	2.007	0.437	0.826	Not performed (no FDR-significant interaction)
Akkermansia	S100B detection	59	Interaction LFC	-0.541	-2.384	1.301	0.570	0.852	Not performed (no FDR-significant interaction)
Coprococcus	S100B detection	59	Interaction LFC	-0.702	-2.216	0.813	0.368	0.826	Not performed (no FDR-significant interaction)
Dorea	S100B detection	59	Interaction LFC	-0.302	-1.216	0.611	0.519	0.852	Not performed (no FDR-significant interaction)
Prevotella	S100B detection	59	Interaction LFC	0.525	-3.054	4.103	0.775	0.913	Not performed (no FDR-significant interaction)
Lachnospiraceae	S100B detection	59	Interaction LFC	0.270	-0.614	1.154	0.552	0.852	Not performed (no FDR-significant interaction)
Enterobacteriaceae	S100B detection	59	Interaction LFC	1.934	-0.232	4.100	0.087	0.443	Not performed (no FDR-significant interaction)
Bacillota	S100B detection	59	Interaction LFC	0.037	-0.844	0.919	0.934	0.934	Not performed (no FDR-significant interaction)
Bacteroidota	S100B detection	59	Interaction LFC	0.206	-0.683	1.096	0.651	0.870	Not performed (no FDR-significant interaction)
Butyricicoccus	GFAP	59	Interaction OR	1.597	0.309	8.241	0.576	0.852	Not applicable (detection model)
Butyricicoccus	NfL	59	Interaction OR	0.924	0.213	4.021	0.917	0.934	Not applicable (detection model)

Butyricicoccus	S100B detection	59	Interaction OR	0.864	0.064	11.726	0.912	0.934	Not applicable (detection model)

**Taxon-by-phenotype interactions for biomarkers were evaluated with adjustment for age and sex. Butyricicoccus detection used logistic regression. q-values were obtained by Benjamini-Hochberg correction across the 51 predefined interaction tests. LFC, log fold change*

Table S8. Age- and sex-adjusted RRMS–SPMS comparisons for predefined SCFA-related pathways

**Pathway**	**Adjusted effect SPMS vs RRMS**	**95% CI**	**p**	**q**
Butyrate production I	Ratio 1.23	0.87–1.75	0.235	0.470
Butyrate production II	Ratio 1.23	0.72–2.10	0.437	0.596
Propionate production II	Ratio 1.29	0.90–1.86	0.167	0.470
Propionate production I, detected	OR 1.56	0.43–5.58	0.496	0.596
Propionate production III, detected	OR 2.98	0.82–10.86	0.097	0.470
Succinate conversion to propionate, detected	OR 0.86	0.23–3.20	0.818	0.818

** Continuous pathways were analysed using multivariable linear regression of natural-log-transformed pathway abundance, whereas sparsely detected pathways were analysed using logistic regression for pathway detection. All models were adjusted for age and sex, with RRMS as the reference group. Exponentiated coefficients are reported as SPMS/RRMS ratios for continuous pathways and odds ratios for detection for sparse pathways. q-values were obtained using the Benjamini–Hochberg procedure across the six predefined comparisons. HC3 heteroscedasticity-consistent standard errors were used as sensitivity analyses for the continuous models. These models constituted the principal disease-phenotype comparison.*

Table S9a. Exploratory whole-profile analysis of gut metabolic modules (GMMs)

**Module**	**Prevalence (%)**	**Analysis**	**Contrast**	**Effect type**	**Estimate**	**95% CI lower**	**95% CI upper**	**p**	**q**
MF0001:arabinoxylan degradation	100.0	Abundance	RRMS vs HC	Adjusted beta, log1p scale	-0.070	-0.179	0.039	0.210	0.863
MF0001:arabinoxylan degradation	100.0	Abundance	SPMS vs HC	Adjusted beta, log1p scale	-0.106	-0.247	0.035	0.142	0.487
MF0001:arabinoxylan degradation	100.0	Abundance	SPMS vs RRMS	Adjusted beta, log1p scale	-0.036	-0.168	0.096	0.593	0.815
MF0002:fructan degradation	100.0	Abundance	RRMS vs HC	Adjusted beta, log1p scale	-0.067	-0.220	0.087	0.395	0.904
MF0002:fructan degradation	100.0	Abundance	SPMS vs HC	Adjusted beta, log1p scale	-0.095	-0.278	0.088	0.310	0.571
MF0002:fructan degradation	100.0	Abundance	SPMS vs RRMS	Adjusted beta, log1p scale	-0.028	-0.204	0.148	0.754	0.850
MF0003:pectin degradation I	98.9	Abundance	RRMS vs HC	Adjusted beta, log1p scale	-0.072	-0.433	0.290	0.698	0.942

Module	Prevalence (%)	Analysis	Contrast	Effect type	Estimate	95% CI lower	95% CI upper	p	q
MF0003:pectin degradation I	98.9	Abundance	SPMS vs HC	Adjusted beta, log1p scale	-0.165	-0.509	0.179	0.347	0.571
MF0003:pectin degradation I	98.9	Abundance	SPMS vs RRMS	Adjusted beta, log1p scale	-0.093	-0.514	0.327	0.663	0.817
MF0004:pectine degradation II	100.0	Abundance	RRMS vs HC	Adjusted beta, log1p scale	-0.008	-0.410	0.395	0.971	0.981
MF0004:pectine degradation II	100.0	Abundance	SPMS vs HC	Adjusted beta, log1p scale	-0.212	-0.608	0.185	0.296	0.571
MF0004:pectine degradation II	100.0	Abundance	SPMS vs RRMS	Adjusted beta, log1p scale	-0.204	-0.625	0.217	0.342	0.764
MF0005:starch degradation	100.0	Abundance	RRMS vs HC	Adjusted beta, log1p scale	-0.097	-0.208	0.014	0.087	0.863
MF0005:starch degradation	100.0	Abundance	SPMS vs HC	Adjusted beta, log1p scale	-0.052	-0.181	0.076	0.425	0.583
MF0005:starch degradation	100.0	Abundance	SPMS vs RRMS	Adjusted beta, log1p scale	0.045	-0.088	0.177	0.508	0.815
MF0006:lactose degradation	100.0	Abundance	RRMS vs HC	Adjusted beta, log1p scale	-0.043	-0.119	0.033	0.268	0.863
MF0006:lactose degradation	100.0	Abundance	SPMS vs HC	Adjusted beta, log1p scale	-0.067	-0.171	0.037	0.210	0.518
MF0006:lactose degradation	100.0	Abundance	SPMS vs RRMS	Adjusted beta, log1p scale	-0.024	-0.117	0.070	0.622	0.815
MF0007:lactose and galactose degradation	76.4	Detection	RRMS vs HC	OR for detection	0.918	0.298	2.829	0.882	0.942
MF0007:lactose and galactose degradation	76.4	Detection	SPMS vs HC	OR for detection	3.375	0.693	16.438	0.132	0.487
MF0007:lactose and galactose degradation	76.4	Detection	SPMS vs RRMS	OR for detection	3.676	0.749	18.035	0.109	0.724
MF0008:maltose degradation	100.0	Abundance	RRMS vs HC	Adjusted beta, log1p scale	-0.017	-0.243	0.210	0.886	0.942
MF0008:maltose degradation	100.0	Abundance	SPMS vs HC	Adjusted beta, log1p scale	0.087	-0.167	0.340	0.503	0.628
MF0008:maltose degradation	100.0	Abundance	SPMS vs RRMS	Adjusted beta, log1p scale	0.103	-0.154	0.360	0.432	0.815
MF0009:melibiose degradation	100.0	Abundance	RRMS vs HC	Adjusted beta, log1p scale	-0.064	-0.143	0.016	0.117	0.863
MF0009:melibiose degradation	100.0	Abundance	SPMS vs HC	Adjusted beta, log1p scale	-0.063	-0.174	0.048	0.266	0.556
MF0009:melibiose degradation	100.0	Abundance	SPMS vs RRMS	Adjusted beta, log1p scale	0.001	-0.112	0.113	0.991	1.000
MF0010:sucrose degradation I	100.0	Abundance	RRMS vs HC	Adjusted beta, log1p scale	-0.115	-0.315	0.085	0.260	0.863
MF0010:sucrose degradation I	100.0	Abundance	SPMS vs HC	Adjusted beta, log1p scale	-0.251	-0.469	-0.034	0.024	0.266
MF0010:sucrose degradation I	100.0	Abundance	SPMS vs RRMS	Adjusted beta, log1p scale	-0.136	-0.379	0.107	0.272	0.764
MF0011:sucrose degradation II	100.0	Abundance	RRMS vs HC	Adjusted beta, log1p scale	-0.049	-0.280	0.183	0.680	0.942
MF0011:sucrose degradation II	100.0	Abundance	SPMS vs HC	Adjusted beta, log1p scale	-0.062	-0.352	0.227	0.673	0.782
MF0011:sucrose degradation II	100.0	Abundance	SPMS vs RRMS	Adjusted beta, log1p scale	-0.014	-0.311	0.283	0.928	0.956
MF0012:trehalose degradation	100.0	Abundance	RRMS vs HC	Adjusted beta, log1p scale	-0.063	-0.294	0.167	0.589	0.942
MF0012:trehalose degradation	100.0	Abundance	SPMS vs HC	Adjusted beta, log1p scale	0.138	-0.145	0.422	0.339	0.571
MF0012:trehalose degradation	100.0	Abundance	SPMS vs RRMS	Adjusted beta, log1p scale	0.202	-0.088	0.492	0.172	0.764
MF0013:allose degradation	95.5	Abundance	RRMS vs HC	Adjusted beta, log1p scale	-0.003	-0.164	0.157	0.969	0.981
MF0013:allose degradation	95.5	Abundance	SPMS vs HC	Adjusted beta, log1p scale	0.150	-0.186	0.486	0.383	0.571
MF0013:allose degradation	95.5	Abundance	SPMS vs RRMS	Adjusted beta, log1p scale	0.153	-0.153	0.459	0.327	0.764
MF0014:arabinose degradation	100.0	Abundance	RRMS vs HC	Adjusted beta, log1p scale	0.352	0.072	0.632	0.014	0.703
MF0014:arabinose degradation	100.0	Abundance	SPMS vs HC	Adjusted beta, log1p scale	0.463	0.145	0.782	0.004	0.167
MF0014:arabinose degradation	100.0	Abundance	SPMS vs RRMS	Adjusted beta, log1p scale	0.111	-0.261	0.483	0.558	0.815
MF0015:fructose degradation	100.0	Abundance	RRMS vs HC	Adjusted beta, log1p scale	0.020	-0.127	0.166	0.794	0.942
MF0015:fructose degradation	100.0	Abundance	SPMS vs HC	Adjusted beta, log1p scale	-0.086	-0.268	0.096	0.357	0.571
MF0015:fructose degradation	100.0	Abundance	SPMS vs RRMS	Adjusted beta, log1p scale	-0.105	-0.270	0.059	0.210	0.764
MF0016:fucose degradation	100.0	Abundance	RRMS vs HC	Adjusted beta, log1p scale	-0.036	-0.277	0.204	0.767	0.942

Module	Prevalence (%)	Analysis	Contrast	Effect type	Estimate	95% CI lower	95% CI upper	p	q
MF0016:fucose degradation	100.0	Abundance	SPMS vs HC	Adjusted beta, log1p scale	0.045	-0.250	0.339	0.767	0.852
MF0016:fucose degradation	100.0	Abundance	SPMS vs RRMS	Adjusted beta, log1p scale	0.081	-0.200	0.361	0.572	0.815
MF0017:galactose degradation	100.0	Abundance	RRMS vs HC	Adjusted beta, log1p scale	0.075	-0.072	0.221	0.317	0.904
MF0017:galactose degradation	100.0	Abundance	SPMS vs HC	Adjusted beta, log1p scale	-0.089	-0.278	0.100	0.357	0.571
MF0017:galactose degradation	100.0	Abundance	SPMS vs RRMS	Adjusted beta, log1p scale	-0.164	-0.336	0.009	0.063	0.720
MF0018:mannose degradation	100.0	Abundance	RRMS vs HC	Adjusted beta, log1p scale	-0.071	-0.200	0.058	0.283	0.884
MF0018:mannose degradation	100.0	Abundance	SPMS vs HC	Adjusted beta, log1p scale	0.063	-0.088	0.214	0.414	0.575
MF0018:mannose degradation	100.0	Abundance	SPMS vs RRMS	Adjusted beta, log1p scale	0.134	-0.000	0.267	0.050	0.720
MF0019:rhamnose degradation	100.0	Abundance	RRMS vs HC	Adjusted beta, log1p scale	0.028	-0.172	0.227	0.786	0.942
MF0019:rhamnose degradation	100.0	Abundance	SPMS vs HC	Adjusted beta, log1p scale	0.008	-0.245	0.262	0.948	0.958
MF0019:rhamnose degradation	100.0	Abundance	SPMS vs RRMS	Adjusted beta, log1p scale	-0.019	-0.262	0.223	0.876	0.942
MF0020:ribose degradation	100.0	Abundance	RRMS vs HC	Adjusted beta, log1p scale	0.159	-0.077	0.396	0.187	0.863
MF0020:ribose degradation	100.0	Abundance	SPMS vs HC	Adjusted beta, log1p scale	0.313	-0.037	0.663	0.080	0.463
MF0020:ribose degradation	100.0	Abundance	SPMS vs RRMS	Adjusted beta, log1p scale	0.154	-0.194	0.501	0.387	0.780
MF0021:xylose degradation	100.0	Abundance	RRMS vs HC	Adjusted beta, log1p scale	-0.001	-0.275	0.273	0.993	0.993
MF0021:xylose degradation	100.0	Abundance	SPMS vs HC	Adjusted beta, log1p scale	0.148	-0.156	0.451	0.340	0.571
MF0021:xylose degradation	100.0	Abundance	SPMS vs RRMS	Adjusted beta, log1p scale	0.149	-0.147	0.445	0.325	0.764
MF0022:galacturonate degradation I	100.0	Abundance	RRMS vs HC	Adjusted beta, log1p scale	-0.091	-0.290	0.108	0.371	0.904
MF0022:galacturonate degradation I	100.0	Abundance	SPMS vs HC	Adjusted beta, log1p scale	-0.079	-0.290	0.132	0.466	0.613
MF0022:galacturonate degradation I	100.0	Abundance	SPMS vs RRMS	Adjusted beta, log1p scale	0.012	-0.206	0.231	0.911	0.956
MF0025:tryptophan degradation	100.0	Abundance	RRMS vs HC	Adjusted beta, log1p scale	0.188	-0.097	0.473	0.195	0.863
MF0025:tryptophan degradation	100.0	Abundance	SPMS vs HC	Adjusted beta, log1p scale	0.429	0.101	0.757	0.010	0.208
MF0025:tryptophan degradation	100.0	Abundance	SPMS vs RRMS	Adjusted beta, log1p scale	0.241	-0.078	0.560	0.139	0.764
MF0026:tyrosine degradation I	100.0	Abundance	RRMS vs HC	Adjusted beta, log1p scale	-0.015	-0.081	0.051	0.657	0.942
MF0026:tyrosine degradation I	100.0	Abundance	SPMS vs HC	Adjusted beta, log1p scale	0.124	0.017	0.232	0.023	0.266
MF0026:tyrosine degradation I	100.0	Abundance	SPMS vs RRMS	Adjusted beta, log1p scale	0.140	0.035	0.244	0.009	0.434
MF0027:tyrosine degradation II	100.0	Abundance	RRMS vs HC	Adjusted beta, log1p scale	0.032	-0.199	0.263	0.786	0.942
MF0027:tyrosine degradation II	100.0	Abundance	SPMS vs HC	Adjusted beta, log1p scale	0.206	-0.085	0.498	0.166	0.502
MF0027:tyrosine degradation II	100.0	Abundance	SPMS vs RRMS	Adjusted beta, log1p scale	0.174	-0.116	0.465	0.240	0.764
MF0028:aspartate degradation I	100.0	Abundance	RRMS vs HC	Adjusted beta, log1p scale	0.039	-0.008	0.086	0.105	0.863
MF0028:aspartate degradation I	100.0	Abundance	SPMS vs HC	Adjusted beta, log1p scale	0.024	-0.045	0.093	0.492	0.622
MF0028:aspartate degradation I	100.0	Abundance	SPMS vs RRMS	Adjusted beta, log1p scale	-0.015	-0.078	0.048	0.643	0.817
MF0029:aspartate degradation II	100.0	Abundance	RRMS vs HC	Adjusted beta, log1p scale	0.050	-0.146	0.246	0.619	0.942
MF0029:aspartate degradation II	100.0	Abundance	SPMS vs HC	Adjusted beta, log1p scale	0.005	-0.224	0.233	0.969	0.969
MF0029:aspartate degradation II	100.0	Abundance	SPMS vs RRMS	Adjusted beta, log1p scale	-0.045	-0.251	0.160	0.666	0.817
MF0030:glutamate degradation I	100.0	Abundance	RRMS vs HC	Adjusted beta, log1p scale	0.116	-0.031	0.264	0.122	0.863
MF0030:glutamate degradation I	100.0	Abundance	SPMS vs HC	Adjusted beta, log1p scale	0.119	-0.079	0.318	0.240	0.540
MF0030:glutamate degradation I	100.0	Abundance	SPMS vs RRMS	Adjusted beta, log1p scale	0.003	-0.205	0.211	0.981	1.000
MF0031:glutamate degradation II	100.0	Abundance	RRMS vs HC	Adjusted beta, log1p scale	0.161	-0.207	0.529	0.391	0.904

Module	Prevalence (%)	Analysis	Contrast	Effect type	Estimate	95% CI lower	95% CI upper	p	q
MF0031:glutamate degradation II	100.0	Abundance	SPMS vs HC	Adjusted beta, log1p scale	0.323	-0.072	0.717	0.109	0.487
MF0031:glutamate degradation II	100.0	Abundance	SPMS vs RRMS	Adjusted beta, log1p scale	0.162	-0.207	0.530	0.390	0.780
MF0032:glutamate degradation III	100.0	Abundance	RRMS vs HC	Adjusted beta, log1p scale	0.200	-0.118	0.517	0.218	0.863
MF0032:glutamate degradation III	100.0	Abundance	SPMS vs HC	Adjusted beta, log1p scale	0.274	-0.128	0.676	0.182	0.503
MF0032:glutamate degradation III	100.0	Abundance	SPMS vs RRMS	Adjusted beta, log1p scale	0.074	-0.348	0.497	0.730	0.850
MF0033:alanine degradation I	100.0	Abundance	RRMS vs HC	Adjusted beta, log1p scale	-0.061	-0.254	0.131	0.530	0.942
MF0033:alanine degradation I	100.0	Abundance	SPMS vs HC	Adjusted beta, log1p scale	0.263	-0.107	0.633	0.164	0.502
MF0033:alanine degradation I	100.0	Abundance	SPMS vs RRMS	Adjusted beta, log1p scale	0.324	-0.024	0.673	0.068	0.720
MF0034:alanine degradation II	100.0	Abundance	RRMS vs HC	Adjusted beta, log1p scale	0.095	-0.135	0.326	0.419	0.910
MF0034:alanine degradation II	100.0	Abundance	SPMS vs HC	Adjusted beta, log1p scale	0.196	-0.069	0.460	0.148	0.487
MF0034:alanine degradation II	100.0	Abundance	SPMS vs RRMS	Adjusted beta, log1p scale	0.100	-0.192	0.393	0.501	0.815
MF0035:glycine degradation	100.0	Abundance	RRMS vs HC	Adjusted beta, log1p scale	0.030	-0.158	0.217	0.757	0.942
MF0035:glycine degradation	100.0	Abundance	SPMS vs HC	Adjusted beta, log1p scale	0.203	-0.026	0.431	0.082	0.463
MF0035:glycine degradation	100.0	Abundance	SPMS vs RRMS	Adjusted beta, log1p scale	0.173	-0.022	0.368	0.082	0.720
MF0036:isoleucine degradation	100.0	Abundance	RRMS vs HC	Adjusted beta, log1p scale	0.017	-0.152	0.186	0.847	0.942
MF0036:isoleucine degradation	100.0	Abundance	SPMS vs HC	Adjusted beta, log1p scale	0.145	-0.172	0.462	0.370	0.571
MF0036:isoleucine degradation	100.0	Abundance	SPMS vs RRMS	Adjusted beta, log1p scale	0.128	-0.162	0.419	0.386	0.780
MF0038:methionine degradation I	100.0	Abundance	RRMS vs HC	Adjusted beta, log1p scale	0.298	0.023	0.574	0.034	0.863
MF0038:methionine degradation I	100.0	Abundance	SPMS vs HC	Adjusted beta, log1p scale	0.382	0.081	0.683	0.013	0.214
MF0038:methionine degradation I	100.0	Abundance	SPMS vs RRMS	Adjusted beta, log1p scale	0.083	-0.236	0.403	0.609	0.815
MF0039:methionine degradation II	100.0	Abundance	RRMS vs HC	Adjusted beta, log1p scale	0.137	-0.094	0.369	0.245	0.863
MF0039:methionine degradation II	100.0	Abundance	SPMS vs HC	Adjusted beta, log1p scale	0.158	-0.073	0.389	0.181	0.503
MF0039:methionine degradation II	100.0	Abundance	SPMS vs RRMS	Adjusted beta, log1p scale	0.020	-0.217	0.258	0.866	0.942
MF0040:proline degradation	100.0	Abundance	RRMS vs HC	Adjusted beta, log1p scale	0.135	-0.165	0.435	0.379	0.904
MF0040:proline degradation	100.0	Abundance	SPMS vs HC	Adjusted beta, log1p scale	0.348	-0.017	0.713	0.062	0.463
MF0040:proline degradation	100.0	Abundance	SPMS vs RRMS	Adjusted beta, log1p scale	0.213	-0.149	0.575	0.250	0.764
MF0041:valine degradation I	75.3	Detection	RRMS vs HC	OR for detection	2.718	0.725	10.192	0.138	0.863
MF0041:valine degradation I	75.3	Detection	SPMS vs HC	OR for detection	0.717	0.196	2.619	0.614	0.731
MF0041:valine degradation I	75.3	Detection	SPMS vs RRMS	OR for detection	0.264	0.059	1.173	0.080	0.720
MF0042:asparagine degradation	100.0	Abundance	RRMS vs HC	Adjusted beta, log1p scale	-0.050	-0.266	0.167	0.652	0.942
MF0042:asparagine degradation	100.0	Abundance	SPMS vs HC	Adjusted beta, log1p scale	0.020	-0.322	0.363	0.908	0.927
MF0042:asparagine degradation	100.0	Abundance	SPMS vs RRMS	Adjusted beta, log1p scale	0.070	-0.244	0.384	0.662	0.817
MF0043:cysteine biosynthesis/homocysteine degradation	62.9	Detection	RRMS vs HC	OR for detection	0.861	0.292	2.537	0.786	0.942
MF0043:cysteine biosynthesis/homocysteine degradation	62.9	Detection	SPMS vs HC	OR for detection	0.861	0.255	2.910	0.810	0.870
MF0043:cysteine biosynthesis/homocysteine degradation	62.9	Detection	SPMS vs RRMS	OR for detection	1.000	0.297	3.371	1.000	1.000
MF0044:cysteine degradation I	100.0	Abundance	RRMS vs HC	Adjusted beta, log1p scale	-0.020	-0.086	0.047	0.562	0.942
MF0044:cysteine degradation I	100.0	Abundance	SPMS vs HC	Adjusted beta, log1p scale	-0.038	-0.111	0.035	0.305	0.571

Module	Prevalence (%)	Analysis	Contrast	Effect type	Estimate	95% CI lower	95% CI upper	p	q
MF0044:cysteine degradation I	100.0	Abundance	SPMS vs RRMS	Adjusted beta, log1p scale	-0.018	-0.093	0.056	0.628	0.815
MF0045:cysteine degradation II	100.0	Abundance	RRMS vs HC	Adjusted beta, log1p scale	-0.034	-0.257	0.189	0.765	0.942
MF0045:cysteine degradation II	100.0	Abundance	SPMS vs HC	Adjusted beta, log1p scale	0.033	-0.298	0.365	0.845	0.899
MF0045:cysteine degradation II	100.0	Abundance	SPMS vs RRMS	Adjusted beta, log1p scale	0.067	-0.241	0.375	0.670	0.817
MF0046:glutamine degradation I	100.0	Abundance	RRMS vs HC	Adjusted beta, log1p scale	0.171	-0.093	0.434	0.204	0.863
MF0046:glutamine degradation I	100.0	Abundance	SPMS vs HC	Adjusted beta, log1p scale	0.362	0.048	0.676	0.024	0.266
MF0046:glutamine degradation I	100.0	Abundance	SPMS vs RRMS	Adjusted beta, log1p scale	0.191	-0.070	0.452	0.151	0.764
MF0047:glutamine degradation II	100.0	Abundance	RRMS vs HC	Adjusted beta, log1p scale	0.047	-0.050	0.145	0.342	0.904
MF0047:glutamine degradation II	100.0	Abundance	SPMS vs HC	Adjusted beta, log1p scale	-0.011	-0.133	0.111	0.860	0.899
MF0047:glutamine degradation II	100.0	Abundance	SPMS vs RRMS	Adjusted beta, log1p scale	-0.058	-0.179	0.062	0.341	0.764
MF0048:serine degradation	100.0	Abundance	RRMS vs HC	Adjusted beta, log1p scale	-0.021	-0.079	0.037	0.476	0.937
MF0048:serine degradation	100.0	Abundance	SPMS vs HC	Adjusted beta, log1p scale	-0.032	-0.087	0.023	0.250	0.543
MF0048:serine degradation	100.0	Abundance	SPMS vs RRMS	Adjusted beta, log1p scale	-0.011	-0.079	0.057	0.746	0.850
MF0049:threonine degradation I	100.0	Abundance	RRMS vs HC	Adjusted beta, log1p scale	0.011	-0.138	0.160	0.885	0.942
MF0049:threonine degradation I	100.0	Abundance	SPMS vs HC	Adjusted beta, log1p scale	-0.147	-0.320	0.027	0.099	0.469
MF0049:threonine degradation I	100.0	Abundance	SPMS vs RRMS	Adjusted beta, log1p scale	-0.157	-0.338	0.023	0.086	0.720
MF0050:threonine degradation II	100.0	Abundance	RRMS vs HC	Adjusted beta, log1p scale	0.171	-0.036	0.378	0.106	0.863
MF0050:threonine degradation II	100.0	Abundance	SPMS vs HC	Adjusted beta, log1p scale	0.155	-0.125	0.435	0.278	0.566
MF0050:threonine degradation II	100.0	Abundance	SPMS vs RRMS	Adjusted beta, log1p scale	-0.016	-0.274	0.242	0.904	0.956
MF0051:arginine degradation I	100.0	Abundance	RRMS vs HC	Adjusted beta, log1p scale	-0.010	-0.122	0.102	0.856	0.942
MF0051:arginine degradation I	100.0	Abundance	SPMS vs HC	Adjusted beta, log1p scale	-0.048	-0.197	0.100	0.525	0.649
MF0051:arginine degradation I	100.0	Abundance	SPMS vs RRMS	Adjusted beta, log1p scale	-0.038	-0.185	0.109	0.614	0.815
MF0052:arginine degradation II	91.0	Abundance	RRMS vs HC	Adjusted beta, log1p scale	-0.035	-0.200	0.130	0.674	0.942
MF0052:arginine degradation II	91.0	Abundance	SPMS vs HC	Adjusted beta, log1p scale	0.134	-0.205	0.472	0.439	0.593
MF0052:arginine degradation II	91.0	Abundance	SPMS vs RRMS	Adjusted beta, log1p scale	0.169	-0.137	0.475	0.278	0.764
MF0054:arginine degradation IV	100.0	Abundance	RRMS vs HC	Adjusted beta, log1p scale	0.271	0.055	0.488	0.014	0.703
MF0054:arginine degradation IV	100.0	Abundance	SPMS vs HC	Adjusted beta, log1p scale	0.399	0.113	0.685	0.006	0.167
MF0054:arginine degradation IV	100.0	Abundance	SPMS vs RRMS	Adjusted beta, log1p scale	0.128	-0.148	0.403	0.364	0.779
MF0055:arginine degradation V	100.0	Abundance	RRMS vs HC	Adjusted beta, log1p scale	0.167	-0.102	0.435	0.225	0.863
MF0055:arginine degradation V	100.0	Abundance	SPMS vs HC	Adjusted beta, log1p scale	0.289	-0.048	0.626	0.093	0.463
MF0055:arginine degradation V	100.0	Abundance	SPMS vs RRMS	Adjusted beta, log1p scale	0.123	-0.229	0.474	0.494	0.815
MF0056:histidine degradation	100.0	Abundance	RRMS vs HC	Adjusted beta, log1p scale	0.182	-0.059	0.424	0.139	0.863
MF0056:histidine degradation	100.0	Abundance	SPMS vs HC	Adjusted beta, log1p scale	0.388	0.108	0.669	0.007	0.167
MF0056:histidine degradation	100.0	Abundance	SPMS vs RRMS	Adjusted beta, log1p scale	0.206	-0.043	0.455	0.105	0.724
MF0057:lysine degradation I	100.0	Abundance	RRMS vs HC	Adjusted beta, log1p scale	0.043	-0.250	0.336	0.773	0.942
MF0057:lysine degradation I	100.0	Abundance	SPMS vs HC	Adjusted beta, log1p scale	0.276	-0.101	0.653	0.151	0.487
MF0057:lysine degradation I	100.0	Abundance	SPMS vs RRMS	Adjusted beta, log1p scale	0.233	-0.132	0.598	0.211	0.764
MF0058:lysine degradation II	100.0	Abundance	RRMS vs HC	Adjusted beta, log1p scale	0.037	-0.136	0.209	0.676	0.942
MF0058:lysine degradation II	100.0	Abundance	SPMS vs HC	Adjusted beta, log1p scale	-0.162	-0.405	0.081	0.191	0.503

Module	Prevalence (%)	Analysis	Contrast	Effect type	Estimate	95% CI lower	95% CI upper	p	q
MF0058:lysine degradation II	100.0	Abundance	SPMS vs RRMS	Adjusted beta, log1p scale	-0.199	-0.432	0.035	0.095	0.724
MF0059:anaerobic fatty acid beta-oxidation	91.0	Abundance	RRMS vs HC	Adjusted beta, log1p scale	-0.033	-0.198	0.132	0.697	0.942
MF0059:anaerobic fatty acid beta-oxidation	91.0	Abundance	SPMS vs HC	Adjusted beta, log1p scale	0.146	-0.192	0.484	0.396	0.574
MF0059:anaerobic fatty acid beta-oxidation	91.0	Abundance	SPMS vs RRMS	Adjusted beta, log1p scale	0.179	-0.127	0.485	0.251	0.764
MF0060:glycerol degradation I	100.0	Abundance	RRMS vs HC	Adjusted beta, log1p scale	-0.045	-0.355	0.265	0.778	0.942
MF0060:glycerol degradation I	100.0	Abundance	SPMS vs HC	Adjusted beta, log1p scale	-0.286	-0.605	0.032	0.078	0.463
MF0060:glycerol degradation I	100.0	Abundance	SPMS vs RRMS	Adjusted beta, log1p scale	-0.242	-0.605	0.121	0.191	0.764
MF0061:glycerol degradation II	100.0	Abundance	RRMS vs HC	Adjusted beta, log1p scale	-0.182	-0.460	0.096	0.199	0.863
MF0061:glycerol degradation II	100.0	Abundance	SPMS vs HC	Adjusted beta, log1p scale	0.328	-0.052	0.708	0.091	0.463
MF0061:glycerol degradation II	100.0	Abundance	SPMS vs RRMS	Adjusted beta, log1p scale	0.510	0.180	0.840	0.002	0.245
MF0063:glyoxylate bypass	93.3	Abundance	RRMS vs HC	Adjusted beta, log1p scale	-0.030	-0.194	0.134	0.720	0.942
MF0063:glyoxylate bypass	93.3	Abundance	SPMS vs HC	Adjusted beta, log1p scale	0.148	-0.189	0.485	0.389	0.572
MF0063:glyoxylate bypass	93.3	Abundance	SPMS vs RRMS	Adjusted beta, log1p scale	0.178	-0.127	0.484	0.253	0.764
MF0064:triacylglycerol degradation	36.0	Detection	RRMS vs HC	OR for detection	0.816	0.286	2.327	0.704	0.942
MF0064:triacylglycerol degradation	36.0	Detection	SPMS vs HC	OR for detection	0.460	0.136	1.558	0.212	0.518
MF0064:triacylglycerol degradation	36.0	Detection	SPMS vs RRMS	OR for detection	0.563	0.162	1.956	0.366	0.779
MF0065:Bifidobacterium shunt	100.0	Abundance	RRMS vs HC	Adjusted beta, log1p scale	0.055	-0.103	0.213	0.497	0.937
MF0065:Bifidobacterium shunt	100.0	Abundance	SPMS vs HC	Adjusted beta, log1p scale	0.147	-0.051	0.346	0.146	0.487
MF0065:Bifidobacterium shunt	100.0	Abundance	SPMS vs RRMS	Adjusted beta, log1p scale	0.093	-0.094	0.279	0.331	0.764
MF0066:Entner-Doudoroff pathway	100.0	Abundance	RRMS vs HC	Adjusted beta, log1p scale	0.178	-0.252	0.608	0.417	0.910
MF0066:Entner-Doudoroff pathway	100.0	Abundance	SPMS vs HC	Adjusted beta, log1p scale	0.331	-0.152	0.814	0.179	0.503
MF0066:Entner-Doudoroff pathway	100.0	Abundance	SPMS vs RRMS	Adjusted beta, log1p scale	0.153	-0.266	0.572	0.475	0.815
MF0067:glycolysis (preparatory phase)	100.0	Abundance	RRMS vs HC	Adjusted beta, log1p scale	0.001	-0.033	0.036	0.942	0.971
MF0067:glycolysis (preparatory phase)	100.0	Abundance	SPMS vs HC	Adjusted beta, log1p scale	0.025	-0.017	0.068	0.243	0.540
MF0067:glycolysis (preparatory phase)	100.0	Abundance	SPMS vs RRMS	Adjusted beta, log1p scale	0.024	-0.017	0.065	0.254	0.764
MF0068:glycolysis (pay-off phase)	100.0	Abundance	RRMS vs HC	Adjusted beta, log1p scale	-0.007	-0.108	0.095	0.897	0.945
MF0068:glycolysis (pay-off phase)	100.0	Abundance	SPMS vs HC	Adjusted beta, log1p scale	-0.043	-0.165	0.078	0.484	0.620
MF0068:glycolysis (pay-off phase)	100.0	Abundance	SPMS vs RRMS	Adjusted beta, log1p scale	-0.037	-0.151	0.078	0.531	0.815
MF0069:NADH:ferredoxin oxidoreductase	64.0	Detection	RRMS vs HC	OR for detection	0.921	0.307	2.764	0.883	0.942
MF0069:NADH:ferredoxin oxidoreductase	64.0	Detection	SPMS vs HC	OR for detection	1.168	0.356	3.831	0.798	0.870
MF0069:NADH:ferredoxin oxidoreductase	64.0	Detection	SPMS vs RRMS	OR for detection	1.269	0.381	4.221	0.698	0.841
MF0070:pentose phosphate pathway (oxidative phase)	100.0	Abundance	RRMS vs HC	Adjusted beta, log1p scale	0.160	-0.242	0.562	0.436	0.928
MF0070:pentose phosphate pathway (oxidative phase)	100.0	Abundance	SPMS vs HC	Adjusted beta, log1p scale	0.352	-0.100	0.804	0.127	0.487
MF0070:pentose phosphate pathway (oxidative phase)	100.0	Abundance	SPMS vs RRMS	Adjusted beta, log1p scale	0.192	-0.201	0.585	0.338	0.764
MF0071:pentose phosphate pathway (non-oxidative branch)	100.0	Abundance	RRMS vs HC	Adjusted beta, log1p scale	0.033	-0.044	0.110	0.398	0.904
MF0071:pentose phosphate pathway (non-oxidative branch)	100.0	Abundance	SPMS vs HC	Adjusted beta, log1p scale	0.008	-0.085	0.102	0.863	0.899
MF0071:pentose phosphate pathway (non-oxidative branch)	100.0	Abundance	SPMS vs RRMS	Adjusted beta, log1p scale	-0.025	-0.110	0.060	0.565	0.815

Module	Prevalence (%)	Analysis	Contrast	Effect type	Estimate	95% CI lower	95% CI upper	p	q
MF0072:pyruvate dehydrogenase complex	55.1	Detection	RRMS vs HC	OR for detection	1.080	0.386	3.019	0.883	0.942
MF0072:pyruvate dehydrogenase complex	55.1	Detection	SPMS vs HC	OR for detection	2.126	0.662	6.833	0.205	0.518
MF0072:pyruvate dehydrogenase complex	55.1	Detection	SPMS vs RRMS	OR for detection	1.968	0.606	6.397	0.260	0.764
MF0073:pyruvate:ferredoxin oxidoreductase	100.0	Abundance	RRMS vs HC	Adjusted beta, log1p scale	0.114	-0.023	0.251	0.102	0.863
MF0073:pyruvate:ferredoxin oxidoreductase	100.0	Abundance	SPMS vs HC	Adjusted beta, log1p scale	0.174	0.007	0.340	0.041	0.414
MF0073:pyruvate:ferredoxin oxidoreductase	100.0	Abundance	SPMS vs RRMS	Adjusted beta, log1p scale	0.059	-0.064	0.182	0.344	0.764
MF0074:pyruvate:formate lyase	100.0	Abundance	RRMS vs HC	Adjusted beta, log1p scale	0.015	-0.100	0.129	0.802	0.942
MF0074:pyruvate:formate lyase	100.0	Abundance	SPMS vs HC	Adjusted beta, log1p scale	-0.050	-0.186	0.086	0.474	0.615
MF0074:pyruvate:formate lyase	100.0	Abundance	SPMS vs RRMS	Adjusted beta, log1p scale	-0.064	-0.193	0.065	0.329	0.764
MF0075:acetate to acetyl-CoA	100.0	Abundance	RRMS vs HC	Adjusted beta, log1p scale	-0.071	-0.228	0.086	0.373	0.904
MF0075:acetate to acetyl-CoA	100.0	Abundance	SPMS vs HC	Adjusted beta, log1p scale	-0.025	-0.221	0.171	0.802	0.870
MF0075:acetate to acetyl-CoA	100.0	Abundance	SPMS vs RRMS	Adjusted beta, log1p scale	0.046	-0.137	0.230	0.620	0.815
MF0076:4-aminobutyrate degradation	100.0	Abundance	RRMS vs HC	Adjusted beta, log1p scale	-0.024	-0.182	0.135	0.768	0.942
MF0076:4-aminobutyrate degradation	100.0	Abundance	SPMS vs HC	Adjusted beta, log1p scale	0.152	-0.174	0.478	0.361	0.571
MF0076:4-aminobutyrate degradation	100.0	Abundance	SPMS vs RRMS	Adjusted beta, log1p scale	0.176	-0.118	0.470	0.241	0.764
MF0077:formate conversion	78.7	Detection	RRMS vs HC	OR for detection	2.233	0.588	8.476	0.238	0.863
MF0077:formate conversion	78.7	Detection	SPMS vs HC	OR for detection	1.349	0.355	5.123	0.660	0.777
MF0077:formate conversion	78.7	Detection	SPMS vs RRMS	OR for detection	0.604	0.135	2.711	0.511	0.815
MF0078:lactaldehyde degradation	100.0	Abundance	RRMS vs HC	Adjusted beta, log1p scale	0.017	-0.165	0.199	0.853	0.942
MF0078:lactaldehyde degradation	100.0	Abundance	SPMS vs HC	Adjusted beta, log1p scale	-0.044	-0.258	0.169	0.684	0.787
MF0078:lactaldehyde degradation	100.0	Abundance	SPMS vs RRMS	Adjusted beta, log1p scale	-0.061	-0.245	0.122	0.512	0.815
MF0079:lactate consumption I	100.0	Abundance	RRMS vs HC	Adjusted beta, log1p scale	0.120	-0.108	0.348	0.303	0.904
MF0079:lactate consumption I	100.0	Abundance	SPMS vs HC	Adjusted beta, log1p scale	0.155	-0.186	0.496	0.373	0.571
MF0079:lactate consumption I	100.0	Abundance	SPMS vs RRMS	Adjusted beta, log1p scale	0.035	-0.274	0.345	0.824	0.915
MF0080:lactate consumption II	94.4	Abundance	RRMS vs HC	Adjusted beta, log1p scale	-0.016	-0.190	0.158	0.857	0.942
MF0080:lactate consumption II	94.4	Abundance	SPMS vs HC	Adjusted beta, log1p scale	0.139	-0.193	0.471	0.411	0.575
MF0080:lactate consumption II	94.4	Abundance	SPMS vs RRMS	Adjusted beta, log1p scale	0.155	-0.149	0.459	0.317	0.764
MF0081:methanol conversion	56.2	Detection	RRMS vs HC	OR for detection	0.523	0.183	1.494	0.226	0.863
MF0081:methanol conversion	56.2	Detection	SPMS vs HC	OR for detection	0.737	0.229	2.379	0.610	0.731
MF0081:methanol conversion	56.2	Detection	SPMS vs RRMS	OR for detection	1.409	0.439	4.524	0.564	0.815
MF0082:putrescine degradation	89.9	Abundance	RRMS vs HC	Adjusted beta, log1p scale	-0.023	-0.188	0.142	0.786	0.942
MF0082:putrescine degradation	89.9	Abundance	SPMS vs HC	Adjusted beta, log1p scale	0.144	-0.194	0.482	0.405	0.575
MF0082:putrescine degradation	89.9	Abundance	SPMS vs RRMS	Adjusted beta, log1p scale	0.166	-0.139	0.472	0.285	0.764
MF0083:succinate consumption	100.0	Abundance	RRMS vs HC	Adjusted beta, log1p scale	0.098	-0.013	0.210	0.085	0.863
MF0083:succinate consumption	100.0	Abundance	SPMS vs HC	Adjusted beta, log1p scale	0.134	-0.018	0.286	0.084	0.463
MF0083:succinate consumption	100.0	Abundance	SPMS vs RRMS	Adjusted beta, log1p scale	0.036	-0.091	0.163	0.582	0.815
MF0085:urea degradation	100.0	Abundance	RRMS vs HC	Adjusted beta, log1p scale	-0.058	-0.271	0.155	0.594	0.942
MF0085:urea degradation	100.0	Abundance	SPMS vs HC	Adjusted beta, log1p scale	0.179	-0.119	0.478	0.239	0.540
MF0085:urea degradation	100.0	Abundance	SPMS vs RRMS	Adjusted beta, log1p scale	0.237	-0.020	0.495	0.071	0.720

Module	Prevalence (%)	Analysis	Contrast	Effect type	Estimate	95% CI lower	95% CI upper	p	q
MF0086:acetyl-CoA to acetate	100.0	Abundance	RRMS vs HC	Adjusted beta, log1p scale	-0.036	-0.111	0.039	0.345	0.904
MF0086:acetyl-CoA to acetate	100.0	Abundance	SPMS vs HC	Adjusted beta, log1p scale	-0.043	-0.134	0.048	0.352	0.571
MF0086:acetyl-CoA to acetate	100.0	Abundance	SPMS vs RRMS	Adjusted beta, log1p scale	-0.007	-0.094	0.080	0.870	0.942
MF0087:acetyl-CoA to crotonyl-CoA	98.9	Abundance	RRMS vs HC	Adjusted beta, log1p scale	-0.024	-0.196	0.148	0.785	0.942
MF0087:acetyl-CoA to crotonyl-CoA	98.9	Abundance	SPMS vs HC	Adjusted beta, log1p scale	0.170	-0.166	0.505	0.321	0.571
MF0087:acetyl-CoA to crotonyl-CoA	98.9	Abundance	SPMS vs RRMS	Adjusted beta, log1p scale	0.194	-0.113	0.501	0.216	0.764
MF0088:butyrate production I	100.0	Abundance	RRMS vs HC	Adjusted beta, log1p scale	0.017	-0.200	0.235	0.876	0.942
MF0088:butyrate production I	100.0	Abundance	SPMS vs HC	Adjusted beta, log1p scale	0.225	-0.054	0.504	0.114	0.487
MF0088:butyrate production I	100.0	Abundance	SPMS vs RRMS	Adjusted beta, log1p scale	0.208	-0.058	0.473	0.125	0.764
MF0089:butyrate production II	100.0	Abundance	RRMS vs HC	Adjusted beta, log1p scale	0.064	-0.218	0.346	0.656	0.942
MF0089:butyrate production II	100.0	Abundance	SPMS vs HC	Adjusted beta, log1p scale	0.269	-0.095	0.632	0.147	0.487
MF0089:butyrate production II	100.0	Abundance	SPMS vs RRMS	Adjusted beta, log1p scale	0.204	-0.147	0.556	0.254	0.764
MF0090:ethanol production I	100.0	Abundance	RRMS vs HC	Adjusted beta, log1p scale	0.064	-0.114	0.242	0.481	0.937
MF0090:ethanol production I	100.0	Abundance	SPMS vs HC	Adjusted beta, log1p scale	0.054	-0.152	0.261	0.605	0.731
MF0090:ethanol production I	100.0	Abundance	SPMS vs RRMS	Adjusted beta, log1p scale	-0.010	-0.193	0.174	0.918	0.956
MF0091:ethanol production II	69.7	Detection	RRMS vs HC	OR for detection	1.164	0.378	3.580	0.792	0.942
MF0091:ethanol production II	69.7	Detection	SPMS vs HC	OR for detection	1.753	0.501	6.136	0.380	0.571
MF0091:ethanol production II	69.7	Detection	SPMS vs RRMS	OR for detection	1.507	0.421	5.394	0.528	0.815
MF0092:lactate production	100.0	Abundance	RRMS vs HC	Adjusted beta, log1p scale	0.086	-0.066	0.238	0.267	0.863
MF0092:lactate production	100.0	Abundance	SPMS vs HC	Adjusted beta, log1p scale	0.127	-0.045	0.298	0.148	0.487
MF0092:lactate production	100.0	Abundance	SPMS vs RRMS	Adjusted beta, log1p scale	0.041	-0.115	0.196	0.610	0.815
MF0094:propionate production II	100.0	Abundance	RRMS vs HC	Adjusted beta, log1p scale	0.108	-0.057	0.272	0.199	0.863
MF0094:propionate production II	100.0	Abundance	SPMS vs HC	Adjusted beta, log1p scale	0.266	-0.008	0.541	0.057	0.463
MF0094:propionate production II	100.0	Abundance	SPMS vs RRMS	Adjusted beta, log1p scale	0.159	-0.098	0.415	0.225	0.764
MF0096:succinate production	62.9	Detection	RRMS vs HC	OR for detection	0.715	0.244	2.090	0.539	0.942
MF0096:succinate production	62.9	Detection	SPMS vs HC	OR for detection	1.097	0.335	3.595	0.879	0.906
MF0096:succinate production	62.9	Detection	SPMS vs RRMS	OR for detection	1.534	0.467	5.040	0.480	0.815
MF0097:homoacetogenesis	100.0	Abundance	RRMS vs HC	Adjusted beta, log1p scale	0.087	-0.144	0.319	0.461	0.937
MF0097:homoacetogenesis	100.0	Abundance	SPMS vs HC	Adjusted beta, log1p scale	0.176	-0.135	0.486	0.267	0.556
MF0097:homoacetogenesis	100.0	Abundance	SPMS vs RRMS	Adjusted beta, log1p scale	0.089	-0.202	0.380	0.550	0.815
MF0098:hydrogen metabolism	100.0	Abundance	RRMS vs HC	Adjusted beta, log1p scale	-0.020	-0.173	0.134	0.803	0.942
MF0098:hydrogen metabolism	100.0	Abundance	SPMS vs HC	Adjusted beta, log1p scale	0.109	-0.128	0.346	0.365	0.571
MF0098:hydrogen metabolism	100.0	Abundance	SPMS vs RRMS	Adjusted beta, log1p scale	0.129	-0.114	0.372	0.299	0.764
MF0099:methanogenesis - methyl-coM	57.3	Detection	RRMS vs HC	OR for detection	0.529	0.186	1.505	0.232	0.863
MF0099:methanogenesis - methyl-coM	57.3	Detection	SPMS vs HC	OR for detection	0.830	0.257	2.686	0.756	0.850
MF0099:methanogenesis - methyl-coM	57.3	Detection	SPMS vs RRMS	OR for detection	1.571	0.488	5.054	0.449	0.815
MF0100:methanogenesis from carbon dioxide	55.1	Detection	RRMS vs HC	OR for detection	0.527	0.185	1.502	0.230	0.863
MF0100:methanogenesis from carbon dioxide	55.1	Detection	SPMS vs HC	OR for detection	0.635	0.198	2.038	0.446	0.594
MF0100:methanogenesis from carbon dioxide	55.1	Detection	SPMS vs RRMS	OR for detection	1.206	0.378	3.845	0.752	0.850

Module	Prevalence (%)	Analysis	Contrast	Effect type	Estimate	95% CI lower	95% CI upper	p	q
MF0101:nitrate reduction (dissimilatory)	100.0	Abundance	RRMS vs HC	Adjusted beta, log1p scale	0.157	-0.178	0.492	0.358	0.904
MF0101:nitrate reduction (dissimilatory)	100.0	Abundance	SPMS vs HC	Adjusted beta, log1p scale	0.213	-0.246	0.672	0.364	0.571
MF0101:nitrate reduction (dissimilatory)	100.0	Abundance	SPMS vs RRMS	Adjusted beta, log1p scale	0.056	-0.297	0.408	0.757	0.850
MF0103:mucin degradation	100.0	Abundance	RRMS vs HC	Adjusted beta, log1p scale	-0.151	-0.344	0.042	0.126	0.863
MF0103:mucin degradation	100.0	Abundance	SPMS vs HC	Adjusted beta, log1p scale	0.034	-0.174	0.243	0.747	0.849
MF0103:mucin degradation	100.0	Abundance	SPMS vs RRMS	Adjusted beta, log1p scale	0.185	-0.014	0.384	0.068	0.720
MF0024:phenylalanine degradation	41.6	Detection	RRMS vs HC	OR for detection	0.956	0.314	2.905	0.936	0.971
MF0024:phenylalanine degradation	41.6	Detection	SPMS vs HC	OR for detection	3.100	0.877	10.951	0.079	0.463
MF0024:phenylalanine degradation	41.6	Detection	SPMS vs RRMS	OR for detection	3.243	0.913	11.522	0.069	0.720
MF0037:leucine degradation	39.3	Detection	RRMS vs HC	OR for detection	0.696	0.247	1.963	0.493	0.937
MF0037:leucine degradation	39.3	Detection	SPMS vs HC	OR for detection	0.473	0.142	1.569	0.221	0.526
MF0037:leucine degradation	39.3	Detection	SPMS vs RRMS	OR for detection	0.680	0.200	2.311	0.536	0.815
MF0062:glycerol degradation III	30.3	Detection	RRMS vs HC	OR for detection	1.572	0.464	5.324	0.467	0.937
MF0062:glycerol degradation III	30.3	Detection	SPMS vs HC	OR for detection	2.346	0.661	8.327	0.187	0.503
MF0062:glycerol degradation III	30.3	Detection	SPMS vs RRMS	OR for detection	1.492	0.436	5.110	0.524	0.815
MF0095:propionate production III	44.9	Detection	RRMS vs HC	OR for detection	1.674	0.564	4.967	0.353	0.904
MF0095:propionate production III	44.9	Detection	SPMS vs HC	OR for detection	5.791	1.628	20.596	0.007	0.167
MF0095:propionate production III	44.9	Detection	SPMS vs RRMS	OR for detection	3.460	1.015	11.790	0.047	0.720
MF0084:succinate conversion to propionate	27.0	Detection	RRMS vs HC	OR for detection	2.617	0.765	8.950	0.125	0.863
MF0084:succinate conversion to propionate	27.0	Detection	SPMS vs HC	OR for detection	2.073	0.528	8.137	0.296	0.571
MF0084:succinate conversion to propionate	27.0	Detection	SPMS vs RRMS	OR for detection	0.792	0.227	2.766	0.715	0.850
MF0093:propionate production I	33.7	Detection	RRMS vs HC	OR for detection	2.185	0.667	7.159	0.197	0.863
MF0093:propionate production I	33.7	Detection	SPMS vs HC	OR for detection	3.096	0.864	11.093	0.083	0.463
MF0093:propionate production I	33.7	Detection	SPMS vs RRMS	OR for detection	1.417	0.426	4.717	0.570	0.815

Of 103 available GMMs, 100 met the predefined prevalence criteria for adjusted analysis. Modules detected in ≥80% of participants were analyzed as abundance measures using age- and sex-adjusted linear regression of log1p-transformed values with HC3 heteroscedasticity-consistent standard errors. Modules detected in 20% to <80% of participants were analyzed as detection outcomes using age- and sex-adjusted logistic regression. Three GMMs detected in <20% of participants were excluded from adjusted inference: MF0102 sulfate reduction (dissimilatory), MF0053 arginine degradation III, and MF0023 galacturonate degradation II. Benjamini-Hochberg FDR correction was applied separately across the 100 GMMs within each pairwise contrast.

Table S9b. Exploratory whole-profile analysis of gut-brain modules (GBMs)

Module	Prevalence (%)	Analysis	Contrast	Effect type	Estimate	95% CI lower	95% CI upper	p	q
MGB001:Serotonin synthesis I	100.0	Abundance	RRMS vs HC	Adjusted beta, log1p scale	0.210	-0.202	0.623	0.318	0.773
MGB001:Serotonin synthesis I	100.0	Abundance	SPMS vs HC	Adjusted beta, log1p scale	0.406	-0.043	0.855	0.076	0.386
MGB001:Serotonin synthesis I	100.0	Abundance	SPMS vs RRMS	Adjusted beta, log1p scale	0.196	-0.200	0.592	0.333	0.732
MGB003:Melatonin synthesis	51.7	Detection	RRMS vs HC	OR for detection	0.729	0.258	2.059	0.551	0.826
MGB003:Melatonin synthesis	51.7	Detection	SPMS vs HC	OR for detection	1.215	0.384	3.847	0.741	0.936

Module	Prevalence (%)	Analysis	Contrast	Effect type	Estimate	95% CI lower	95% CI upper	p	q
MGB003:Melatonin synthesis	51.7	Detection	SPMS vs RRMS	OR for detection	1.666	0.517	5.366	0.392	0.732
MGB005:Tryptophan synthesis	100.0	Abundance	RRMS vs HC	Adjusted beta, log1p scale	-0.075	-0.224	0.074	0.325	0.773
MGB005:Tryptophan synthesis	100.0	Abundance	SPMS vs HC	Adjusted beta, log1p scale	-0.030	-0.215	0.155	0.752	0.936
MGB005:Tryptophan synthesis	100.0	Abundance	SPMS vs RRMS	Adjusted beta, log1p scale	0.045	-0.131	0.221	0.616	0.896
MGB006:Glutamate synthesis I	100.0	Abundance	RRMS vs HC	Adjusted beta, log1p scale	0.029	-0.058	0.115	0.513	0.794
MGB006:Glutamate synthesis I	100.0	Abundance	SPMS vs HC	Adjusted beta, log1p scale	0.064	-0.024	0.153	0.154	0.386
MGB006:Glutamate synthesis I	100.0	Abundance	SPMS vs RRMS	Adjusted beta, log1p scale	0.035	-0.069	0.140	0.507	0.869
MGB007:Glutamate synthesis II	100.0	Abundance	RRMS vs HC	Adjusted beta, log1p scale	0.037	-0.058	0.133	0.443	0.773
MGB007:Glutamate synthesis II	100.0	Abundance	SPMS vs HC	Adjusted beta, log1p scale	-0.003	-0.123	0.118	0.967	0.994
MGB007:Glutamate synthesis II	100.0	Abundance	SPMS vs RRMS	Adjusted beta, log1p scale	-0.040	-0.157	0.078	0.506	0.869
MGB008:Acetylcholine synthesis	100.0	Abundance	RRMS vs HC	Adjusted beta, log1p scale	0.138	-0.131	0.408	0.315	0.773
MGB008:Acetylcholine synthesis	100.0	Abundance	SPMS vs HC	Adjusted beta, log1p scale	0.343	-0.042	0.729	0.081	0.386
MGB008:Acetylcholine synthesis	100.0	Abundance	SPMS vs RRMS	Adjusted beta, log1p scale	0.205	-0.183	0.593	0.301	0.732
MGB009:Histamine synthesis	100.0	Abundance	RRMS vs HC	Adjusted beta, log1p scale	0.185	-0.223	0.594	0.374	0.773
MGB009:Histamine synthesis	100.0	Abundance	SPMS vs HC	Adjusted beta, log1p scale	-0.003	-0.478	0.473	0.990	0.994
MGB009:Histamine synthesis	100.0	Abundance	SPMS vs RRMS	Adjusted beta, log1p scale	-0.188	-0.594	0.218	0.364	0.732
MGB010:Histamine degradation	100.0	Abundance	RRMS vs HC	Adjusted beta, log1p scale	0.064	-0.108	0.235	0.467	0.773
MGB010:Histamine degradation	100.0	Abundance	SPMS vs HC	Adjusted beta, log1p scale	0.267	0.024	0.511	0.032	0.379
MGB010:Histamine degradation	100.0	Abundance	SPMS vs RRMS	Adjusted beta, log1p scale	0.204	-0.046	0.454	0.110	0.673
MGB011:Cortisol degradation	100.0	Abundance	RRMS vs HC	Adjusted beta, log1p scale	0.081	-0.279	0.441	0.659	0.860
MGB011:Cortisol degradation	100.0	Abundance	SPMS vs HC	Adjusted beta, log1p scale	-0.315	-0.732	0.102	0.138	0.386
MGB011:Cortisol degradation	100.0	Abundance	SPMS vs RRMS	Adjusted beta, log1p scale	-0.396	-0.839	0.047	0.080	0.673
MGB012:Dopamine synthesis	92.1	Abundance	RRMS vs HC	Adjusted beta, log1p scale	-0.026	-0.088	0.036	0.411	0.773
MGB012:Dopamine synthesis	92.1	Abundance	SPMS vs HC	Adjusted beta, log1p scale	0.012	-0.070	0.093	0.780	0.936
MGB012:Dopamine synthesis	92.1	Abundance	SPMS vs RRMS	Adjusted beta, log1p scale	0.038	-0.034	0.109	0.305	0.732
MGB015:p-Cresol synthesis	100.0	Abundance	RRMS vs HC	Adjusted beta, log1p scale	0.023	-0.129	0.176	0.763	0.887
MGB015:p-Cresol synthesis	100.0	Abundance	SPMS vs HC	Adjusted beta, log1p scale	0.049	-0.149	0.248	0.625	0.882
MGB015:p-Cresol synthesis	100.0	Abundance	SPMS vs RRMS	Adjusted beta, log1p scale	0.026	-0.127	0.179	0.739	0.910
MGB016:p-Cresol degradation	57.3	Detection	RRMS vs HC	OR for detection	0.827	0.284	2.409	0.728	0.877
MGB016:p-Cresol degradation	57.3	Detection	SPMS vs HC	OR for detection	2.321	0.688	7.832	0.175	0.396
MGB016:p-Cresol degradation	57.3	Detection	SPMS vs RRMS	OR for detection	2.806	0.824	9.549	0.099	0.673
MGB019:GABA degradation	100.0	Abundance	RRMS vs HC	Adjusted beta, log1p scale	-0.003	-0.243	0.236	0.977	0.977
MGB019:GABA degradation	100.0	Abundance	SPMS vs HC	Adjusted beta, log1p scale	0.025	-0.262	0.312	0.865	0.966
MGB019:GABA degradation	100.0	Abundance	SPMS vs RRMS	Adjusted beta, log1p scale	0.028	-0.268	0.324	0.851	0.929
MGB021:GABA synthesis II	89.9	Abundance	RRMS vs HC	Adjusted beta, log1p scale	-0.022	-0.187	0.143	0.795	0.887
MGB021:GABA synthesis II	89.9	Abundance	SPMS vs HC	Adjusted beta, log1p scale	0.145	-0.193	0.483	0.399	0.737
MGB021:GABA synthesis II	89.9	Abundance	SPMS vs RRMS	Adjusted beta, log1p scale	0.167	-0.138	0.472	0.283	0.732
MGB022:GABA synthesis III	100.0	Abundance	RRMS vs HC	Adjusted beta, log1p scale	0.161	-0.207	0.529	0.391	0.773
MGB022:GABA synthesis III	100.0	Abundance	SPMS vs HC	Adjusted beta, log1p scale	0.323	-0.072	0.717	0.109	0.386

Module	Prevalence (%)	Analysis	Contrast	Effect type	Estimate	95% CI lower	95% CI upper	p	q
MGB022:GABA synthesis III	100.0	Abundance	SPMS vs RRMS	Adjusted beta, log1p scale	0.162	-0.207	0.530	0.390	0.732
MGB023:Dopamine degradation	95.5	Abundance	RRMS vs HC	Adjusted beta, log1p scale	-0.011	-0.037	0.015	0.396	0.773
MGB023:Dopamine degradation	95.5	Abundance	SPMS vs HC	Adjusted beta, log1p scale	0.042	-0.013	0.097	0.134	0.386
MGB023:Dopamine degradation	95.5	Abundance	SPMS vs RRMS	Adjusted beta, log1p scale	0.053	0.003	0.103	0.036	0.673
MGB024:DOPAC synthesis	73.0	Detection	RRMS vs HC	OR for detection	1.638	0.513	5.237	0.405	0.773
MGB024:DOPAC synthesis	73.0	Detection	SPMS vs HC	OR for detection	2.139	0.595	7.688	0.244	0.488
MGB024:DOPAC synthesis	73.0	Detection	SPMS vs RRMS	OR for detection	1.306	0.343	4.964	0.696	0.910
MGB025:Nitric oxide synthesis I (NO synthase)	100.0	Abundance	RRMS vs HC	Adjusted beta, log1p scale	0.045	-0.205	0.295	0.726	0.877
MGB025:Nitric oxide synthesis I (NO synthase)	100.0	Abundance	SPMS vs HC	Adjusted beta, log1p scale	-0.015	-0.293	0.264	0.917	0.994
MGB025:Nitric oxide synthesis I (NO synthase)	100.0	Abundance	SPMS vs RRMS	Adjusted beta, log1p scale	-0.060	-0.316	0.197	0.648	0.910
MGB026:Nitric oxide synthesis II (nitrite reductase)	100.0	Abundance	RRMS vs HC	Adjusted beta, log1p scale	0.127	-0.182	0.437	0.419	0.773
MGB026:Nitric oxide synthesis II (nitrite reductase)	100.0	Abundance	SPMS vs HC	Adjusted beta, log1p scale	0.135	-0.304	0.575	0.546	0.858
MGB026:Nitric oxide synthesis II (nitrite reductase)	100.0	Abundance	SPMS vs RRMS	Adjusted beta, log1p scale	0.008	-0.325	0.342	0.962	0.981
MGB027:Nitric oxide degradation I (NO dioxygenase)	100.0	Abundance	RRMS vs HC	Adjusted beta, log1p scale	0.061	-0.176	0.299	0.612	0.860
MGB027:Nitric oxide degradation I (NO dioxygenase)	100.0	Abundance	SPMS vs HC	Adjusted beta, log1p scale	0.153	-0.168	0.474	0.349	0.670
MGB027:Nitric oxide degradation I (NO dioxygenase)	100.0	Abundance	SPMS vs RRMS	Adjusted beta, log1p scale	0.092	-0.239	0.423	0.585	0.878
MGB029:ClpB (ATP-dependent chaperone protein)	100.0	Abundance	RRMS vs HC	Adjusted beta, log1p scale	-0.001	-0.005	0.003	0.663	0.860
MGB029:ClpB (ATP-dependent chaperone protein)	100.0	Abundance	SPMS vs HC	Adjusted beta, log1p scale	-0.007	-0.015	0.002	0.120	0.386
MGB029:ClpB (ATP-dependent chaperone protein)	100.0	Abundance	SPMS vs RRMS	Adjusted beta, log1p scale	-0.006	-0.015	0.004	0.234	0.732
MGB031:17-beta-Estradiol degradation	100.0	Abundance	RRMS vs HC	Adjusted beta, log1p scale	-0.022	-0.063	0.019	0.293	0.773
MGB031:17-beta-Estradiol degradation	100.0	Abundance	SPMS vs HC	Adjusted beta, log1p scale	-0.015	-0.064	0.033	0.529	0.858
MGB031:17-beta-Estradiol degradation	100.0	Abundance	SPMS vs RRMS	Adjusted beta, log1p scale	0.006	-0.039	0.052	0.781	0.929
MGB032:Quinolinic acid synthesis	100.0	Abundance	RRMS vs HC	Adjusted beta, log1p scale	-0.067	-0.200	0.067	0.328	0.773
MGB032:Quinolinic acid synthesis	100.0	Abundance	SPMS vs HC	Adjusted beta, log1p scale	0.042	-0.113	0.197	0.595	0.879
MGB032:Quinolinic acid synthesis	100.0	Abundance	SPMS vs RRMS	Adjusted beta, log1p scale	0.109	-0.053	0.271	0.188	0.721
MGB033:Quinolinic acid degradation	100.0	Abundance	RRMS vs HC	Adjusted beta, log1p scale	-0.074	-0.205	0.058	0.271	0.773
MGB033:Quinolinic acid degradation	100.0	Abundance	SPMS vs HC	Adjusted beta, log1p scale	0.049	-0.114	0.213	0.554	0.858
MGB033:Quinolinic acid degradation	100.0	Abundance	SPMS vs RRMS	Adjusted beta, log1p scale	0.123	-0.040	0.286	0.138	0.673
MGB034:Isovaleric acid synthesis I (KADH pathway)	60.7	Detection	RRMS vs HC	OR for detection	1.057	0.377	2.959	0.917	0.950
MGB034:Isovaleric acid synthesis I (KADH pathway)	60.7	Detection	SPMS vs HC	OR for detection	2.378	0.708	7.988	0.161	0.386
MGB034:Isovaleric acid synthesis I (KADH pathway)	60.7	Detection	SPMS vs RRMS	OR for detection	2.251	0.659	7.686	0.195	0.721
MGB035:Isovaleric acid synthesis II (KADC pathway)	73.0	Detection	RRMS vs HC	OR for detection	0.587	0.187	1.848	0.363	0.773
MGB035:Isovaleric acid synthesis II (KADC pathway)	73.0	Detection	SPMS vs HC	OR for detection	1.052	0.280	3.951	0.940	0.994
MGB035:Isovaleric acid synthesis II (KADC pathway)	73.0	Detection	SPMS vs RRMS	OR for detection	1.792	0.496	6.476	0.374	0.732
MGB036:S-Adenosylmethionine (SAM) synthesis	100.0	Abundance	RRMS vs HC	Adjusted beta, log1p scale	-0.008	-0.050	0.035	0.731	0.877
MGB036:S-Adenosylmethionine (SAM) synthesis	100.0	Abundance	SPMS vs HC	Adjusted beta, log1p scale	0.006	-0.049	0.062	0.828	0.965
MGB036:S-Adenosylmethionine (SAM) synthesis	100.0	Abundance	SPMS vs RRMS	Adjusted beta, log1p scale	0.014	-0.029	0.057	0.532	0.878
MGB037:Inositol synthesis	100.0	Abundance	RRMS vs HC	Adjusted beta, log1p scale	0.162	-0.190	0.513	0.367	0.773
MGB037:Inositol synthesis	100.0	Abundance	SPMS vs HC	Adjusted beta, log1p scale	-0.065	-0.420	0.290	0.720	0.936

Module	Prevalence (%)	Analysis	Contrast	Effect type	Estimate	95% CI lower	95% CI upper	p	q
MGB037:Inositol synthesis	100.0	Abundance	SPMS vs RRMS	Adjusted beta, log1p scale	-0.227	-0.528	0.074	0.140	0.673
MGB038:Inositol degradation	100.0	Abundance	RRMS vs HC	Adjusted beta, log1p scale	0.126	-0.064	0.316	0.192	0.773
MGB038:Inositol degradation	100.0	Abundance	SPMS vs HC	Adjusted beta, log1p scale	0.191	-0.044	0.425	0.111	0.386
MGB038:Inositol degradation	100.0	Abundance	SPMS vs RRMS	Adjusted beta, log1p scale	0.065	-0.154	0.283	0.563	0.878
MGB039:g-Hydroxybutyric acid (GHB) degradation	100.0	Abundance	RRMS vs HC	Adjusted beta, log1p scale	0.059	-0.111	0.229	0.494	0.790
MGB039:g-Hydroxybutyric acid (GHB) degradation	100.0	Abundance	SPMS vs HC	Adjusted beta, log1p scale	0.078	-0.133	0.290	0.467	0.800
MGB039:g-Hydroxybutyric acid (GHB) degradation	100.0	Abundance	SPMS vs RRMS	Adjusted beta, log1p scale	0.019	-0.211	0.249	0.871	0.929
MGB040:Menaquinone synthesis (vitamin K2) I	100.0	Abundance	RRMS vs HC	Adjusted beta, log1p scale	-0.152	-0.497	0.192	0.385	0.773
MGB040:Menaquinone synthesis (vitamin K2) I	100.0	Abundance	SPMS vs HC	Adjusted beta, log1p scale	-0.038	-0.413	0.337	0.844	0.965
MGB040:Menaquinone synthesis (vitamin K2) I	100.0	Abundance	SPMS vs RRMS	Adjusted beta, log1p scale	0.115	-0.284	0.513	0.572	0.878
MGB041:Menaquinone synthesis (vitamin K2) II (alternative pathway: futalosine pathway)	100.0	Abundance	RRMS vs HC	Adjusted beta, log1p scale	0.181	-0.140	0.502	0.268	0.773
MGB041:Menaquinone synthesis (vitamin K2) II (alternative pathway: futalosine pathway)	100.0	Abundance	SPMS vs HC	Adjusted beta, log1p scale	0.441	0.068	0.815	0.021	0.331
MGB041:Menaquinone synthesis (vitamin K2) II (alternative pathway: futalosine pathway)	100.0	Abundance	SPMS vs RRMS	Adjusted beta, log1p scale	0.260	-0.109	0.629	0.168	0.721
MGB043:Acetate synthesis I	100.0	Abundance	RRMS vs HC	Adjusted beta, log1p scale	0.035	-0.085	0.155	0.568	0.826
MGB043:Acetate synthesis I	100.0	Abundance	SPMS vs HC	Adjusted beta, log1p scale	0.038	-0.107	0.183	0.604	0.879
MGB043:Acetate synthesis I	100.0	Abundance	SPMS vs RRMS	Adjusted beta, log1p scale	0.003	-0.130	0.136	0.960	0.981
MGB044:Acetate synthesis II	100.0	Abundance	RRMS vs HC	Adjusted beta, log1p scale	0.182	-0.001	0.364	0.051	0.773
MGB044:Acetate synthesis II	100.0	Abundance	SPMS vs HC	Adjusted beta, log1p scale	0.208	-0.038	0.453	0.097	0.386
MGB044:Acetate synthesis II	100.0	Abundance	SPMS vs RRMS	Adjusted beta, log1p scale	0.026	-0.244	0.295	0.852	0.929
MGB045:Acetate synthesis III	100.0	Abundance	RRMS vs HC	Adjusted beta, log1p scale	-0.030	-0.299	0.239	0.826	0.901
MGB045:Acetate synthesis III	100.0	Abundance	SPMS vs HC	Adjusted beta, log1p scale	-0.066	-0.370	0.238	0.670	0.919
MGB045:Acetate synthesis III	100.0	Abundance	SPMS vs RRMS	Adjusted beta, log1p scale	-0.036	-0.314	0.242	0.801	0.929
MGB047:Acetate degradation	100.0	Abundance	RRMS vs HC	Adjusted beta, log1p scale	-0.060	-0.220	0.099	0.457	0.773
MGB047:Acetate degradation	100.0	Abundance	SPMS vs HC	Adjusted beta, log1p scale	-0.028	-0.220	0.164	0.776	0.936
MGB047:Acetate degradation	100.0	Abundance	SPMS vs RRMS	Adjusted beta, log1p scale	0.033	-0.154	0.219	0.732	0.910
MGB049:Tryptophan degradation	100.0	Abundance	RRMS vs HC	Adjusted beta, log1p scale	0.188	-0.097	0.473	0.195	0.773
MGB049:Tryptophan degradation	100.0	Abundance	SPMS vs HC	Adjusted beta, log1p scale	0.429	0.101	0.757	0.010	0.249
MGB049:Tryptophan degradation	100.0	Abundance	SPMS vs RRMS	Adjusted beta, log1p scale	0.241	-0.078	0.560	0.139	0.673
MGB050:Glutamate degradation I	100.0	Abundance	RRMS vs HC	Adjusted beta, log1p scale	0.116	-0.031	0.264	0.122	0.773
MGB050:Glutamate degradation I	100.0	Abundance	SPMS vs HC	Adjusted beta, log1p scale	0.119	-0.079	0.318	0.240	0.488
MGB050:Glutamate degradation I	100.0	Abundance	SPMS vs RRMS	Adjusted beta, log1p scale	0.003	-0.205	0.211	0.981	0.981
MGB051:Glutamate degradation II	100.0	Abundance	RRMS vs HC	Adjusted beta, log1p scale	0.200	-0.118	0.517	0.218	0.773
MGB051:Glutamate degradation II	100.0	Abundance	SPMS vs HC	Adjusted beta, log1p scale	0.274	-0.128	0.676	0.182	0.396
MGB051:Glutamate degradation II	100.0	Abundance	SPMS vs RRMS	Adjusted beta, log1p scale	0.074	-0.348	0.497	0.730	0.910
MGB052:Butyrate synthesis I	100.0	Abundance	RRMS vs HC	Adjusted beta, log1p scale	0.017	-0.200	0.235	0.876	0.935
MGB052:Butyrate synthesis I	100.0	Abundance	SPMS vs HC	Adjusted beta, log1p scale	0.225	-0.054	0.504	0.114	0.386
MGB052:Butyrate synthesis I	100.0	Abundance	SPMS vs RRMS	Adjusted beta, log1p scale	0.208	-0.058	0.473	0.125	0.673

Module	Prevalence (%)	Analysis	Contrast	Effect type	Estimate	95% CI lower	95% CI upper	p	q
MGB053:Butyrate synthesis II	100.0	Abundance	RRMS vs HC	Adjusted beta, log1p scale	0.064	-0.218	0.346	0.656	0.860
MGB053:Butyrate synthesis II	100.0	Abundance	SPMS vs HC	Adjusted beta, log1p scale	0.269	-0.095	0.632	0.147	0.386
MGB053:Butyrate synthesis II	100.0	Abundance	SPMS vs RRMS	Adjusted beta, log1p scale	0.204	-0.147	0.556	0.254	0.732
MGB054:Propionate synthesis II	100.0	Abundance	RRMS vs HC	Adjusted beta, log1p scale	0.108	-0.057	0.272	0.199	0.773
MGB054:Propionate synthesis II	100.0	Abundance	SPMS vs HC	Adjusted beta, log1p scale	0.266	-0.008	0.541	0.057	0.386
MGB054:Propionate synthesis II	100.0	Abundance	SPMS vs RRMS	Adjusted beta, log1p scale	0.159	-0.098	0.415	0.225	0.732
MGB056:Propionate degradation I	89.9	Abundance	RRMS vs HC	Adjusted beta, log1p scale	-0.023	-0.185	0.138	0.778	0.887
MGB056:Propionate degradation I	89.9	Abundance	SPMS vs HC	Adjusted beta, log1p scale	0.134	-0.200	0.468	0.431	0.766
MGB056:Propionate degradation I	89.9	Abundance	SPMS vs RRMS	Adjusted beta, log1p scale	0.157	-0.146	0.460	0.309	0.732
MGB004:Kynurenine synthesis	58.4	Detection	RRMS vs HC	OR for detection	1.666	0.566	4.899	0.354	0.773
MGB004:Kynurenine synthesis	58.4	Detection	SPMS vs HC	OR for detection	0.996	0.315	3.147	0.994	0.994
MGB004:Kynurenine synthesis	58.4	Detection	SPMS vs RRMS	OR for detection	0.598	0.182	1.963	0.396	0.732
MGB020:GABA synthesis I	43.8	Detection	RRMS vs HC	OR for detection	0.953	0.325	2.798	0.930	0.950
MGB020:GABA synthesis I	43.8	Detection	SPMS vs HC	OR for detection	2.393	0.713	8.033	0.158	0.386
MGB020:GABA synthesis I	43.8	Detection	SPMS vs RRMS	OR for detection	2.511	0.741	8.511	0.139	0.673
MGB055:Propionate synthesis III	44.9	Detection	RRMS vs HC	OR for detection	1.674	0.564	4.967	0.353	0.773
MGB055:Propionate synthesis III	44.9	Detection	SPMS vs HC	OR for detection	5.791	1.628	20.596	0.007	0.249
MGB055:Propionate synthesis III	44.9	Detection	SPMS vs RRMS	OR for detection	3.460	1.015	11.790	0.047	0.673
MGB048:Propionate synthesis I	34.8	Detection	RRMS vs HC	OR for detection	2.570	0.789	8.373	0.117	0.773
MGB048:Propionate synthesis I	34.8	Detection	SPMS vs HC	OR for detection	3.223	0.892	11.648	0.074	0.386
MGB048:Propionate synthesis I	34.8	Detection	SPMS vs RRMS	OR for detection	1.254	0.378	4.158	0.711	0.910
MGB018:Kynurenine degradation	20.2	Detection	RRMS vs HC	OR for detection	4.826	0.892	26.106	0.068	0.773
MGB018:Kynurenine degradation	20.2	Detection	SPMS vs HC	OR for detection	4.194	0.731	24.053	0.108	0.386
MGB018:Kynurenine degradation	20.2	Detection	SPMS vs RRMS	OR for detection	0.869	0.222	3.395	0.840	0.929

Of 49 available GBMs, 48 met the predefined prevalence criteria for adjusted analysis. Modules detected in ≥80% of participants were analyzed as abundance measures using age- and sex-adjusted linear regression of log1p-transformed values with HC3 heteroscedasticity-consistent standard errors. Modules detected in 20% to <80% of participants were analyzed as detection outcomes using age- and sex-adjusted logistic regression. MGB028 nitric oxide degradation II (NO reductase), detected in <20% of participants, was excluded from adjusted inference. Benjamini-Hochberg FDR correction was applied separately across the 48 GBMs within each pairwise contrast.

Table S10a. Associations of predefined SCFA-related microbial functions with neurological disability

Pathway	Outcome	N	Effect type	Estimate	95% CI lower	95% CI upper	p	q	HC3 p
Butyrate production I	EDSS	59	Proportional-odds OR	1.181	0.701	1.990	0.532	0.898	—
Butyrate production II	EDSS	59	Proportional-odds OR	1.207	0.729	1.998	0.465	0.898	—
Propionate production II	EDSS	59	Proportional-odds OR	1.119	0.675	1.857	0.663	0.898	—

Propionate production I, detected	EDSS	59	Proportional-odds OR	1.073	0.419	2.751	0.883	0.898	—
Propionate production III, detected	EDSS	59	Proportional-odds OR	1.912	0.738	4.953	0.182	0.601	—
Succinate conversion to propionate, detected	EDSS	59	Proportional-odds OR	1.310	0.485	3.538	0.594	0.898	—
Butyrate production I	T25FW	59	Completion-time ratio	1.015	0.913	1.128	0.781	0.898	0.770
Butyrate production II	T25FW	59	Completion-time ratio	0.992	0.894	1.101	0.882	0.898	0.884
Propionate production II	T25FW	59	Completion-time ratio	0.969	0.873	1.077	0.565	0.898	0.674
Propionate production I, detected	T25FW	59	Completion-time ratio	0.905	0.736	1.113	0.349	0.838	0.364
Propionate production III, detected	T25FW	59	Completion-time ratio	1.177	0.961	1.442	0.122	0.544	0.124
Succinate conversion to propionate, detected	T25FW	59	Completion-time ratio	0.901	0.726	1.117	0.346	0.838	0.380
Butyrate production I	9HPT, dominant hand	59	Completion-time ratio	1.072	0.980	1.172	0.136	0.544	0.388
Butyrate production II	9HPT, dominant hand	59	Completion-time ratio	1.103	1.012	1.202	0.030	0.199	0.133
Propionate production II	9HPT, dominant hand	59	Completion-time ratio	1.013	0.924	1.109	0.790	0.898	0.837
Propionate production I, detected	9HPT, dominant hand	59	Completion-time ratio	1.025	0.856	1.227	0.788	0.898	0.804
Propionate production III, detected	9HPT, dominant hand	59	Completion-time ratio	1.212	1.020	1.439	0.033	0.199	0.041
Succinate conversion to propionate, detected	9HPT, dominant hand	59	Completion-time ratio	1.028	0.852	1.239	0.777	0.898	0.812
Butyrate production I	9HPT, non-dominant hand	58	Completion-time ratio	1.104	1.014	1.202	0.026	0.199	0.111
Butyrate production II	9HPT, non-dominant hand	58	Completion-time ratio	1.105	1.016	1.202	0.023	0.199	0.102
Propionate production II	9HPT, non-dominant hand	58	Completion-time ratio	1.016	0.930	1.110	0.726	0.898	0.764
Propionate production I, detected	9HPT, non-dominant hand	58	Completion-time ratio	0.988	0.828	1.180	0.898	0.898	0.903
Propionate production III, detected	9HPT, non-dominant hand	58	Completion-time ratio	1.121	0.943	1.332	0.200	0.601	0.239
Succinate conversion to propionate, detected	9HPT, non-dominant hand	58	Completion-time ratio	1.013	0.844	1.217	0.887	0.898	0.900

**Models were fitted among participants with MS and adjusted for phenotype, age, and sex. Continuous GMMs were natural-log transformed and standardised; sparse GMMs were analysed as detection variables. EDSS used proportional-odds regression; T25FW and 9HPT outcomes were natural-log transformed. HC3 p-values are shown for continuous-outcome sensitivity analyses where applicable. q-values were obtained by Benjamini-Hochberg correction across the 24 predefined GMM-disability associations.*

Table S10b. SCFA-related microbial function-by-phenotype interactions for neurological disability

Pathway	Outcome	N	Interaction beta	95% CI lower	95% CI upper	p interaction	q interaction
Butyrate production I	EDSS	59	-0.852	-1.882	0.178	0.102	0.813
Butyrate production II	EDSS	59	-0.258	-1.289	0.772	0.622	0.932
Propionate production II	EDSS	59	-0.259	-1.344	0.826	0.641	0.932
Propionate production I, detected	EDSS	59	-1.058	-2.978	0.863	0.279	0.853
Propionate production III, detected	EDSS	59	0.641	-1.286	2.569	0.514	0.932
Succinate conversion to propionate, detected	EDSS	59	-1.739	-3.745	0.267	0.087	0.813
Butyrate production I	T25FW	59	0.003	-0.210	0.216	0.979	0.979
Butyrate production II	T25FW	59	0.086	-0.130	0.303	0.439	0.932
Propionate production II	T25FW	59	-0.110	-0.343	0.122	0.355	0.853
Propionate production I, detected	T25FW	59	-0.060	-0.479	0.360	0.782	0.979
Propionate production III, detected	T25FW	59	-0.099	-0.509	0.310	0.637	0.932
Succinate conversion to propionate, detected	T25FW	59	-0.266	-0.699	0.167	0.234	0.853
Butyrate production I	9HPT, dominant hand	59	-0.041	-0.221	0.140	0.660	0.932
Butyrate production II	9HPT, dominant hand	59	0.046	-0.134	0.226	0.618	0.932
Propionate production II	9HPT, dominant hand	59	-0.021	-0.223	0.182	0.841	0.979
Propionate production I, detected	9HPT, dominant hand	59	-0.027	-0.393	0.339	0.885	0.979
Propionate production III, detected	9HPT, dominant hand	59	0.292	-0.047	0.631	0.097	0.813
Succinate conversion to propionate, detected	9HPT, dominant hand	59	-0.208	-0.586	0.169	0.285	0.853
Butyrate production I	9HPT, non-dominant hand	58	0.028	-0.143	0.199	0.753	0.979
Butyrate production II	9HPT, non-dominant hand	58	0.094	-0.079	0.267	0.291	0.853

Propionate production II	9HPT, non-dominant hand	58	-0.004	-0.202	0.193	0.967	0.979
Propionate production I, detected	9HPT, non-dominant hand	58	-0.171	-0.527	0.184	0.349	0.853
Propionate production III, detected	9HPT, non-dominant hand	58	-0.012	-0.361	0.337	0.947	0.979
Succinate conversion to propionate, detected	9HPT, non-dominant hand	58	-0.226	-0.594	0.141	0.233	0.853

**Interaction terms assessed whether GMM-disability associations differed between RRMS and SPMS, with adjustment for age and sex. q-values were obtained by Benjamini-Hochberg correction across the 24 predefined interaction tests.*

Table S10c. Sensitivity analysis of SCFA-related microbial functions and EDSS treated as a numeric outcome

Pathway	Beta	HC3 SE	HC3 p	q
Butyrate production I	0.101	0.227	0.658	0.927
Butyrate production II	0.143	0.225	0.527	0.927
Propionate production II	0.023	0.253	0.927	0.927
Propionate production I, detected	0.094	0.363	0.797	0.927
Propionate production III, detected	0.430	0.358	0.235	0.927
Succinate conversion to propionate, detected	0.153	0.403	0.706	0.927

**Sensitivity analysis treating EDSS as a numeric outcome in age- and sex-adjusted linear models controlling for phenotype, with HC3 heteroscedasticity-consistent standard errors. q-values were obtained by Benjamini-Hochberg correction across the six predefined GMM-EDSS associations.*

Table S11a. Associations of predefined SCFA-related microbial functions with circulating biomarkers

Pathway	Outcome	N	Effect type	Estimate	95% CI lower	95% CI upper	p	q	HC3 p
Butyrate production I	GFAP	59	mean difference	5.408	-10.482	21.298	0.508	0.990	0.524
Butyrate production II	GFAP	59	mean difference	1.607	-14.105	17.319	0.842	0.990	0.813
Propionate production II	GFAP	59	mean difference	-1.376	-17.293	14.540	0.866	0.990	0.864
Propionate production I, detected	GFAP	59	mean difference	-18.334	-49.365	12.696	0.252	0.990	0.276
Propionate production III, detected	GFAP	59	mean difference	-22.176	-52.965	8.612	0.164	0.983	0.186
Succinate conversion to propionate, detected	GFAP	59	mean difference	8.286	-24.404	40.976	0.621	0.990	0.629
Butyrate production I	NfL	59	ratio	0.959	0.839	1.097	0.547	0.990	0.555
Butyrate production II	NfL	59	ratio	0.955	0.836	1.089	0.493	0.990	0.463
Propionate production II	NfL	59	ratio	1.001	0.875	1.145	0.990	0.990	0.992
Propionate production I, detected	NfL	59	ratio	1.275	0.986	1.648	0.070	0.821	0.152
Propionate production III, detected	NfL	59	ratio	0.982	0.753	1.279	0.892	0.990	0.883
Succinate conversion to propionate, detected	NfL	59	ratio	1.135	0.862	1.493	0.371	0.990	0.377
Butyrate production I	S100B detection	59	OR	1.010	0.581	1.754	0.973	0.990	—
Butyrate production II	S100B detection	59	OR	1.074	0.622	1.853	0.798	0.990	—
Propionate production II	S100B detection	59	OR	1.118	0.641	1.950	0.695	0.990	—
Propionate production I, detected	S100B detection	59	OR	1.095	0.368	3.254	0.870	0.990	—
Propionate production III, detected	S100B detection	59	OR	0.554	0.182	1.686	0.298	0.990	—
Succinate conversion to propionate, detected	S100B detection	59	OR	2.809	0.847	9.316	0.091	0.821	—

**Models were fitted among participants with MS and adjusted for phenotype, age, and sex. GFAP was analysed on the original scale, NfL after natural-log transformation, and S100B as detectable versus non-detectable. HC3 p-values are shown for continuous biomarker models where applicable. q-values were obtained by Benjamini-Hochberg correction across the 18 predefined GMM-biomarker associations.*

Table S11b. SCFA-related microbial function-by-phenotype interactions for circulating biomarkers

Pathway	Outcome	N	Interaction beta	95% CI lower	95% CI upper	p interaction	q interaction
Butyrate production I	GFAP	59	-6.837	-38.838	25.164	0.677	0.846
Butyrate production II	GFAP	59	-6.634	-39.496	26.229	0.694	0.846
Propionate production II	GFAP	59	-2.443	-37.856	32.971	0.893	0.893
Propionate production I, detected	GFAP	59	-8.722	-71.822	54.378	0.788	0.846
Propionate production III, detected	GFAP	59	48.260	-12.595	109.115	0.126	0.392
Succinate conversion to propionate, detected	GFAP	59	-22.813	-89.134	43.508	0.503	0.823
Butyrate production I	NfL	59	0.128	-0.140	0.397	0.354	0.637
Butyrate production II	NfL	59	0.079	-0.197	0.355	0.578	0.846
Propionate production II	NfL	59	-0.253	-0.544	0.038	0.094	0.392
Propionate production I, detected	NfL	59	-0.657	-1.150	-0.165	0.012	0.208
Propionate production III, detected	NfL	59	0.471	-0.049	0.990	0.082	0.392
Succinate conversion to propionate, detected	NfL	59	-0.307	-0.860	0.246	0.282	0.634
Butyrate production I	S100B detection	59	-0.535	-1.673	0.604	0.350	0.637
Butyrate production II	S100B detection	59	-1.045	-2.289	0.199	0.086	0.392
Propionate production II	S100B detection	59	0.696	-0.570	1.961	0.271	0.634
Propionate production I, detected	S100B detection	59	-0.293	-2.483	1.897	0.793	0.846
Propionate production III, detected	S100B detection	59	1.722	-0.538	3.982	0.131	0.392
Succinate conversion to propionate, detected	S100B detection	59	0.317	-2.132	2.767	0.799	0.846

**Interaction terms assessed whether GMM-biomarker associations differed between RRMS and SPMS, with adjustment for age and sex. q-values were obtained by Benjamini-Hochberg correction across the 18 predefined interaction tests.*

Table S12a. Exploratory nitric oxide- and redox-related microbial functions: overall three-group comparisons

Pathway	Analysis	p	q
MGB025 – Nitric oxide synthesis I	Continuous abundance	0.629	0.943
MGB026 – Nitric oxide synthesis II	Continuous abundance	0.477	0.943
MGB027 – Nitric oxide degradation I	Continuous abundance	0.189	0.943
MF0070 – Pentose phosphate pathway, oxidative phase	Continuous abundance	0.375	0.943
MF0069 – NADH:ferredoxin oxidoreductase, detected	Detection	0.888	1.000
MGB028 – Nitric oxide degradation II, detected	Detection	1.000	1.000

Exploratory overall comparisons across HC, RRMS, and SPMS. Continuous functions were compared as abundance measures and sparse functions by detection status. q-values were obtained by Benjamini-Hochberg correction across the six exploratory functions.

Table S12b. Exploratory nitric oxide- and redox-related microbial functions: unadjusted RRMS-SPMS comparisons

Pathway	Effect type	Estimate	95% CI lower	95% CI upper	p	q
MGB025 – Nitric oxide synthesis I	Hodges-Lehmann difference, SPMS vs RRMS	0.229	-0.250	0.776	0.343	0.947
MGB026 – Nitric oxide synthesis II	Hodges-Lehmann difference, SPMS vs RRMS	0.188	-1.072	1.684	0.756	0.947
MGB027 – Nitric oxide degradation I	Hodges-Lehmann difference, SPMS vs RRMS	-0.113	-0.887	0.546	0.779	0.947
MF0070 – Pentose phosphate pathway, oxidative phase	Hodges-Lehmann difference, SPMS vs RRMS	-1.478	-6.884	3.040	0.375	0.947

MF0069 – NADH:ferredoxin oxidoreductase, detected	OR for detection, SPMS vs RRMS	0.793	0.239	2.580	0.789	0.947
MGB028 – Nitric oxide degradation II, detected	OR for detection, SPMS vs RRMS	0.966	0.012	78.416	1.000	1.000

**Exploratory unadjusted RRMS-SPMS comparisons. Continuous functions are reported as Hodges-Lehmann location shifts; sparse functions as odds ratios for detection. q-values were obtained by Benjamini-Hochberg correction across the six exploratory functions.*

Table S12c. Exploratory nitric oxide- and redox-related microbial functions: age- and sex-adjusted RRMS-SPMS comparisons

Pathway	Effect type	Estimate	95% CI lower	95% CI upper	p	q	HC3 p
MGB025 – Nitric oxide synthesis I	SPMS/RRMS ratio	0.865	0.487	1.538	0.624	0.755	0.642
MGB026 – Nitric oxide synthesis II	SPMS/RRMS ratio	1.201	0.845	1.706	0.312	0.624	0.352
MGB027 – Nitric oxide degradation I	SPMS/RRMS ratio	1.325	0.715	2.455	0.374	0.624	0.463
MF0070 – Pentose phosphate pathway, oxidative phase	SPMS/RRMS ratio	1.318	0.800	2.172	0.282	0.624	0.207
MF0069 – NADH:ferredoxin oxidoreductase, detected	OR detection, SPMS vs RRMS	1.226	0.341	4.408	0.755	0.755	—

**Exploratory RRMS-SPMS models adjusted for age and sex. Exponentiated coefficients are reported as SPMS/RRMS ratios for continuous functions and as odds ratios for detection for sparse functions. HC3 p-values are shown for continuous models where applicable. q-values were obtained across the five functions eligible for adjusted modelling.*

Table S13. Exploratory sensitivity analysis according to current disease-modifying treatment status among participants with SPMS

Alpha diversity

Outcome	Untreated SPMS (n = 13)	Treated SPMS (n = 17)	p
Shannon	4.121 (3.422–4.233)	4.098 (3.917–4.259)	0.336
Simpson	0.954 (0.938–0.968)	0.964 (0.955–0.975)	0.167

Beta diversity

Analysis	Result	p
Bray–Curtis PERMANOVA, unadjusted	$R^2 = 0.027$	0.854
Bray–Curtis PERMANOVA, adjusted for age and sex	$R^2 = 0.027$	0.876
PERMDISP	0.555 untreated vs. 0.554 treated	0.978

Predefined SCFA-related functional pathways

Pathway	Untreated SPMS (n = 13)	Treated SPMS (n = 17)	p	q
Butyrate production I	8.684 (3.879–8.813)	9.656 (8.352–12.987)	0.112	0.246
Butyrate production II	6.176 (2.711–6.671)	6.308 (4.096–7.671)	0.477	0.572
Propionate production II	3.364 (2.536–4.878)	3.359 (2.142–4.554)	0.645	0.645
Propionate production I, detected	8/13 (61.5%)	6/17 (35.3%)	0.269	0.403
Propionate production III, detected	11/13 (84.6%)	8/17 (47.1%)	0.057	0.246
Succinate conversion to propionate, detected	6/13 (46.2%)	3/17 (17.6%)	0.123	0.246

Continuous variables are presented as median (IQR) and were compared using Wilcoxon rank-sum tests. Detection outcomes are presented as n/N (%) and were compared using Fisher's exact tests. Benjamini–Hochberg FDR correction was applied across the six predefined SCFA-related functional comparisons. PERMANOVA and PERMDISP analyses were based on Bray–Curtis dissimilarities. These analyses were exploratory and restricted to participants with SPMS.
